# Supplementary material for: Prognostic Impact of Serum Free Light Chain Ratio Normalization in Patients with Multiple Myeloma Treated within the GMMG-MM5 Trial
Source: Cancers (Basel). 2021 Sep 28;13(19):4856. doi: 10.3390/cancers13194856 (PMC8507729; doi:10.3390/cancers13194856)
Supplement: Supplementary file 1 [file cancers-13-04856-s001.zip › cancers-1383719-supplementary.pdf]

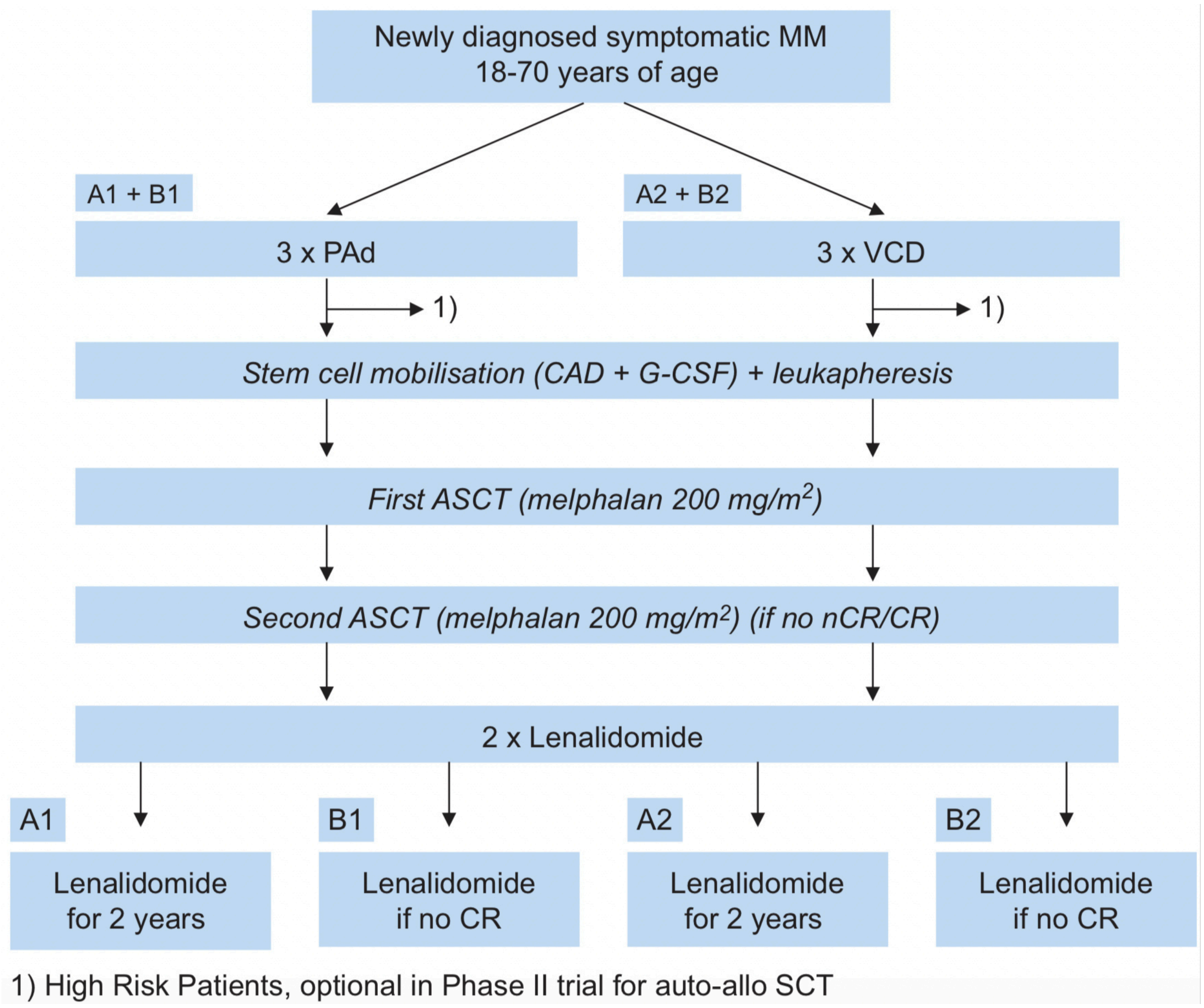

Figure S1. Flow chart diagram of the treatment within the MM5 trial [18].
Abbreviations: ASCT, autologous stem cell transplantation; CAD, cyclophosphamide/doxorubicin/dexamethasone; CR, complete remission; G-CSF, granulocyte-colony stimulating factor; MM, multiple myeloma; nCR, near complete remission; PAd, bortezomib/doxorubicin/dexamethasone; VCD, bortezomib/cyclophosphamide/dexamethasone.

Table S1. Results of time-dependent multivariate Cox regression analysis: Impact of achieved FLCr normalization at the fixed time point end of induction therapy on PFS and OS.

Variable	PFS		OS	
	**HR (95% CI)**	**P-value**	**HR (95% CI)**	**P-value**
Age (per year)	1.00 (0.98 - 1.01)	0.57	1.01 (0.99 - 1.04)	0.16
ISS (II vs. I)	1.46 (1.12 - 1.89)	<0.01	1.79 (1.17 - 2.74)	0.01
ISS (III vs. I)	1.91 (1.43 - 2.55)	<0.01	3.03 (1.97 - 4.65)	<0.01
Adverse cytogenetics (yes vs. no)	2.10 (1.66 - 2.65)	<0.01	3.06 (2.13 - 4.39)	<0.01
Treatment arm (B vs. A)	1.07 (0.85 - 1.34)	0.59	1.58 (1.14 - 2.20)	0.01
Response after IT (CR vs. non-CR)	0.73 (0.46 - 1.15)	0.18	0.31 (0.10 - 1.00)	0.05
FLCr normalization IT (yes vs. no)	0.81 (0.62 - 1.05)	0.11	0.75 (0.50 - 1.12)	0.16

Abbreviations: CR, complete remission; HR, hazard ratio; ISS, International Staging System; IT, induction therapy; OS, overall survival; PFS, progression-free survival; FLCr, free light chain ratio; 95% CI, 95% confidence interval.

Table S2. Results of time-dependent multivariate Cox regression analysis: Impact of achieved FLCr normalization at the fixed time point end of consolidation therapy on PFS and OS.

Variable	PFS		OS	
	**HR (95% CI)**	**P-value**	**HR (95% CI)**	**P-value**
Age (per year)	1.00 (0.98 - 1.01)	0.67	1.02 (1.00 - 1.04)	0.09
ISS (II vs. I)	1.46 (1.13 - 1.90)	<0.01	1.79 (1.17 - 2.75)	0.01
ISS (III vs. I)	1.92 (1.44 - 2.55)	<0.01	3.07 (2.01 - 4.69)	<0.01
Adverse cytogenetics (yes vs. no)	2.07 (1.64 - 2.61)	<0.01	2.95 (2.07 - 4.21)	<0.01
Treatment arm (B vs. A)	1.04 (0.83 - 1.30)	0.74	1.57 (1.13 - 2.19)	0.01
Response after CONS (CR vs. non-CR)	0.98 (0.73 - 1.32)	0.90	0.65 (0.40 - 1.05)	0.08
FLCr normalization CONS (yes vs. no)	0.82 (0.64 - 1.05)	0.12	0.85 (0.60 - 1.19)	0.34

Abbreviations: CONS, consolidation.

Table S3. Results of time-dependent multivariate Cox regression analysis: Impact of achieved immune reconstitution at the fixed time point end of consolidation therapy on PFS and OS.

Variable	PFS		OS	
	**HR (95% CI)**	**P-value**	**HR (95% CI)**	**P-value**
Age (per year)	1.00 (0.98 - 1.01)	0.78	1.02 (1.00 - 1.04)	0.04
ISS (II vs. I)	1.45 (1.12 - 1.88)	0.01	1.76 (1.15 - 2.69)	0.01
ISS (III vs. I)	1.90 (1.43 - 2.53)	<0.01	3.00 (1.97 - 4.58)	<0.01
Adverse cytogenetics (yes vs. no)	2.09 (1.66 - 2.64)	<0.01	3.05 (2.14 - 4.35)	<0.01
Treatment arm (B vs. A)	1.04 (0.83 - 1.31)	0.71	1.59 (1.14 - 2.22)	0.01
Response after CONS (CR vs. non-CR)	0.98 (0.73 - 1.30)	0.87	0.65 (0.40 - 1.03)	0.07
IR CONS (yes vs. no)	0.83 (0.64 - 1.07)	0.15	0.54 (0.36 - 0.83)	<0.01

Abbreviations: IR, immune reconstitution.

Material S1. Clinical trial protocol.

CLINICAL TRIAL PROTOCOL

MM5 Trial

Randomised phase III trial for previously untreated multiple myeloma to evaluate two regimens of bortezomib based induction therapy and lenalidomide consolidation followed by lenalidomide maintenance treatment

Clinical Trial Code: MM5

EudraCT No.: 2010-019173-16

Clinical Phase: Phase III

Version: 3.0 - 06.06.2013

CONFIDENTIAL: This protocol contains confidential information and is intended solely for the guidance of the clinical investigation. This protocol may not be disclosed to parties not associated with the clinical investigation or used for any purpose without the prior written consent of the principal investigator/ coordinating investigator.

CONTACT DATA

PRINCIPAL INVESTIGATOR/ COORDINATING INVESTIGATOR (LEITER DER KLINISCHEN PRÜFUNG*)	SPONSOR
**Prof. Dr. med. H. Goldschmidt**<br>Universitätsklinikum Heidelberg<br>Medizinische Klinik V und<br>Nationales Centrum für Tumorerkrankungen (NCT)<br>Im Neuenheimer Feld 410<br>69120 Heidelberg, Germany<br>Phone: +49-(0)6221-56-8003<br>Fax: +49-(0)6221-56-5647<br>e-mail:<br>hartmut.goldschmidt@med.uni-heidelberg.de<br>** According to § 4 German Drug Law (AMG)*	**Universität Heidelberg represented by the Director of Administration Irmtraut Gürkan**<br>Im Neuenheimer Feld 672<br>69120 Heidelberg, Germany<br>Phone: +49-(0)6221-56-7002<br>Fax: +49-(0)6221-56-4888<br>e-mail:<br>irmtraut.guerkan@med.uni-heidelberg.de

BIOMETRICIAN	TRIAL COORDINATOR
**Dipl.-Stat. T. Hielscher**<br>Abteilung Biostatistik<br>Deutsches Krebsforschungszentrum (dkfz)<br>Im Neuenheimer Feld 581<br>69120 Heidelberg, Germany<br>Phone: +49-(0)6221-42-2386<br>Fax: +49-(0)6221-42-2397<br>e-mail: t.hielscher@dkfz-heidelberg.de	**Dr. med. U. Bertsch**<br>GMMG Studiensekretariat<br>Universitätsklinikum Heidelberg<br>Medizinische Klinik V und<br>Nationales Centrum für Tumorerkrankungen<br>Im Neuenheimer Feld 350<br>69120 Heidelberg, Germany<br>Phone: +49-(0)6221-56-8015 /-8198<br>Fax: +49-(0)6221-56-1957<br>e-mail: uta.bertsch@med.uni-heidelberg.de

SCIENTIFIC COORDINATOR	STUDY OFFICE
**Dr. D. Hose**<br>Labor für Myelomforschung<br>Universitätsklinikum Heidelberg<br>Medizinische Klinik V und<br>Nationales Centrum für Tumorerkrankungen<br>Im Neuenheimer Feld 350<br>69120 Heidelberg, Germany<br>Phone: +49-(0)6221-56-6140<br>Fax: +49-(0)6221-56-6238<br>e-mail: dirk.hose@med.uni-heidelberg.de	**GMMG Studiensekretariat**<br>Universitätsklinikum Heidelberg<br>Medizinische Klinik V und<br>Nationales Centrum für Tumorerkrankungen<br>Im Neuenheimer Feld 350<br>69120 Heidelberg, Germany<br>Phone: +49-(0)6221-56-8198<br>Fax: +49-(0)6221-56-1957<br>e-mail: studiensekretariat.gmmg@med.uni-heidelberg.de

CLINICAL MONITORING	DATA MANAGEMENT
**KKS Heidelberg**<br>Dipl. Biol. A. Gamer (Ltg. Monitoring)<br>Voßstr. 2 / Gebäude 4410<br>69115 Heidelberg, Germany<br>Phone: +49-(0)6221 / 56-39039<br>Fax: +49-(0)6221 / 56-33508<br>e-mail:anna-lena.gamer@med.uni-heidelberg.de	**KKS Heidelberg**<br>Christina Klose (Ltg. DM)<br>Voßstr. 2 / Gebäude 4410<br>69115 Heidelberg, Germany<br>Phone: +49-(0)6221 / 56-36215<br>Fax: +49-(0)6221 / 56-1331<br>e-mail:<br>klose@imbi.uni-heidelberg.de

PHARMACOVIGILANCE / SAE REPORTING

KKS Heidelberg
Dr. med. J. Hajda
Voßstr. 2 / Gebäude 4410
69115 Heidelberg, Germany
Phone: +49-(0)6221 / 56-34507
Fax: +49-(0)6221 / 56-33725
e-mail: kks_sae@med.uni-heidelberg.de

DATA SAFETY MONITORING BOARD (DSMB)

Hematology

Prof. G. Gahrton
Department of Medicine
Karolinska Institute
Karolinska University Hospital Huddinge
SE14186 Stockholm, Sweden
Phone: +46 8 58582469
Fax: +46 8 7748725
e-mail: gosta.gahrton@ki.se

Prof. M. Attal
Hématologie
Hôpital Purpan
Place du Dr. Baylac
31059 Toulouse, France
Phone: +33 5 61777784
Fax: +33 5 61777541
e-mail: attal.m@chu-toulouse.fr

Biostatistics

Dr. rer. nat. Dipl.-Math. L. Edler
Am Schafgarten 27
67373 Dudenhofen, Germany
Phone: +49-(0)6232 94620
e-mail: edler@dkfz.de

ADVISORY BOARD GMMG

Priv.-Doz. Dr. med. I. W. Blau
Prof. Dr. med. A. Pezzutto
Klinik für Hämatologie, Onkologie und Tumorimmunologie, Charité
Campus Benjamin Franklin
Hindenburgdamm 30
12200 **Berlin**, Germany
Phone: +49-(0)30-8445-4430
Fax: +49-(0)30-8445-4468
e-mail: igor.blau@charite.de

Prof. Dr. med. W. Knauf
Gemeinschaftspraxis Onkologie
Im Prüfling 17-19
60389 **Frankfurt/Main**, Germany
Phone: +49-(0)69-45 10 80
Fax: +49-(0)69-45 82 57
e-mail: Onkologie-Frankfurt@telemed.de

Prof. Dr. med. I. Schmidt-Wolf
Prof. Dr. med. P. Brossart
Med. Universitätsklinik und Poliklinik III
Sigmund-Freud-Str. 25
53105 **Bonn**, Germany
Phone: +49-(0)228-287-15489
Fax: +49-(0)228-287-15849
e-mail: Ingo.Schmidt-Wolf@ukb.uni-bonn.de

Dr. med. G. Schließer
Priv.-Doz. Dr. med. A. Käbisch
Praxis für Innere Medizin
Hämatologie/Onkologie
Wingertshecke 6
35392 **Gießen**, Germany
Phone: +49-(0)641-9203-190
Fax: +49-(0)641-9203-192
e-mail: onkologie-giessen@t-online.de

Dr. med. A. Hänel
Priv.-Doz. Dr. med. M. Hänel
Klinikum Chemnitz GmbH
Innere Medizin III
Bürgerstr. 2
09113 **Chemnitz**, Germany
Phone: +49-(0)371-333-43048
Fax: +49-(0)371-333-43047
e-mail: a.haenel@skc.de

Dr. med. H.-J. Salwender
Prof. Dr. med. Christian Meyer zum Büschenfelde
Asklepios Klinik Altona
II. Medizinische Abteilung
Paul-Ehrlich-Str. 1
22763 **Hamburg**, Germany
Phone: +49-(0)40-8822-1211
Fax: +49-(0)40-8822-4950
e-mail: h.salwender@asklepios.com

Dr. med. N. Peter
Prof. Dr. med. H. B. Steinhauer
Carl-Thiem-Klinikum Cottbus
Akad. Lehrkrankenhaus der Charité
II. Medizinische Klinik
Thiemstr. 111
03003 **Cottbus**, Germany
Phone: +49-(0)355-46-2223/2023
Fax: +49-(0)355-46-2611
e-mail: N.Peter@ctk.de

Prof. Dr. med. H. Goldschmidt
Dr. D. Hose
Prof. Dr. med. A. D. Ho
Medizinische Klinik V
Universitätsklinik Heidelberg
Im Neuenheimer Feld 410
69120 **Heidelberg**, Germany
Phone: +49-(0)6221-56-8003
Fax: +49-(0)6221-56-5647
e-mail:
hartmut.goldschmidt@med.uni-heidelberg.de

Priv.-Doz. Dr. med. B. Lathan
Praxis für Hämatologie/Onkologie
Am Oelpfad 12
44263 **Dortmund**, Germany
Phone: +49-(0)231-9140-920
Fax: +49-(0)231-16 46 33
e-mail: b.lathan@t-online.de

Priv.-Doz. Dr. C. Scheid
Prof. Dr. med. M. Hallek
Universität Köln
Klinik I Innere Medizin
Joseph-Stelzmann-Straße 9
50925 **Köln**, Germany
Phone: +49-(0)221-478-4400
Fax: +49-(0)221-478-7170
e-mail: c.scheid@uni-koeln.de

Priv.-Doz. Dr. med. J. Dürig
Prof. Dr. med. U. Dührsen
Westdeutsches Tumorzentrum
Klinik für Hämatologie
Universitätsklinikum Essen
Hufelandstr. 55
45147 Essen, Germany
Phone: +49-(0)201-723-2553
Fax: +49-(0)201-723-5928
e-mail: haema.studien@uk-essen.de

PD Dr. K. Weisel
Prof. Dr. L. Kanz
Medizinische Klinik Universität Tübingen
Abtl. II/Hämatologie, Onkologie, Immunologie und Rheumatologie
Otfried-Müller-Str. 10
72076 **Tübingen**, Germany
Phone: +49-(0)7071-29-82711
Fax : +49-(0)7071-29-3671
e-mail: katja.weisel@med.uni-tuebingen.de

Priv.-Doz. Dr. med. H. Martin
Prof. Dr. med. H. Serve
Medizinische Klinik III
Universitätsklinik Frankfurt
Theodor-Stern-Kai 7
60596 **Frankfurt/Main**, Germany
Phone: +49-(0)69-6301-7376
Fax: +49-(0)69-6301-7326
e-mail: h.martin@em.uni-frankfurt.de

CENTRAL DIAGNOSTICS – Laboratory Assessments

ALL SAMPLES SHOULD BE SENT TO

Medizinische Klinik V
Leitstelle
MM5 Trial
INF 410
69120 Heidelberg, Germany

Plasma Cell Purification Gene Expression Profiling, MRD-FACS	***iFISH***
**Dr. D. Hose**<br>Labor für Myelomforschung<br>Universitätsklinikum Heidelberg<br>Medizinische Klinik V und<br>Nationales Centrum für Tumorerkrankungen (NCT)<br>Im Neuenheimer Feld 350<br>69120 Heidelberg, Germany<br>Phone: +49-(0)6221-56-7772<br>Fax: +49-(0)6221-56-6238<br>e-mail: dirk.hose@med.uni-heidelberg.de	**Prof. Dr. A. Jauch**<br>Molekulare Cytogenetik<br>Institut für Humangenetik<br>Universitätsklinikum Heidelberg<br>Im Neuenheimer Feld 366<br>69120 Heidelberg, Germany<br>Phone: +49-(0)6221-56-5063<br>Fax: +49-(0)6221-56-5091<br>e-mail: anna.jauch@med.uni-heidelberg.de
MRD ASO-PCR	***Freelite and Hevylite Test***
**Dr. N. Weinhold**<br>Molekularbiologisches Labor<br>Universitätsklinikum Heidelberg<br>Medizinische Klinik V<br>Im Neuenheimer Feld 410<br>69120 Heidelberg, Germany<br>Phone: +49-(0)6221-56-36194<br>Fax: +49-(0)6221-56- 5609<br>e-mail: niels.weinhold@med.uni-heidelberg.de	**M. Zorn**<br>Zentrallabor<br>Universitätsklinikum Heidelberg<br>Im Neuenheimer Feld 410<br>69120 Heidelberg, Germany<br>Phone: +49-(0)6221-56-39028<br>Fax: +49-(0)6221-56-4612<br>e-mail: markus.zorn@med.uni-heidelberg.de

CENTRAL DIAGNOSTICS – Imaging

CT (optional 2nd assessment)	*MRT (optional 2nd assessment)*
**Prof. Dr. M. Horger**<br>Abt. für Diagnostische und Interventionelle Radiologie<br>Universitätsklinikum Tübingen<br>Hoppe-Seyler-Straße 3<br>72076 Tübingen, Germany<br>Phone: +49-(0)7071-29-83973<br>Fax : +49-(0)7071-29-4478<br>e-mail: marius.horger@med.uni-tuebingen.de	**Prof. Dr. S. Delorme**<br>Deutsches Krebsforschungszentrum (dkfz)<br>Abteilung Radiologie<br>Im Neuenheimer Feld 280<br>69120 Heidelberg, Germany<br>Phone: +49-(0)6221-42-2563<br>Fax: +49-(0)6221-42-2567<br>e-mail: s.delorme@dkfz-heidelberg.de
and	*and*
**PD Dr. K. Weisel**<br>Medizinische Klinik II / Hämatologie, Onkologie<br>Universitätsklinikum Tübingen<br>Otfried-Müller-Str. 10<br>72076 Tübingen, Germany<br>Phone: +49-(0)7071-29-82711<br>Fax : +49-(0)7071-29-3671<br>e-mail: katja.weisel@med.uni-tuebingen.de	**Dr. J. Hillengaß**<br>Medizinische Klinik V<br>Universitätsklinikum Heidelberg<br>Im Neuenheimer Feld 410<br>69120 Heidelberg, Germany<br>Phone: +49-(0)6221-56-8003<br>Fax: +49-(0)6221-56-5647<br>e-mail:<br>jens.hillengass@med.uni-heidelberg.de

The GMMG study group is member of the Competence Network Malignant Lymphomas

PARTICIPATING SITES

All participating sites are listed in an extra document available at the GMMG study office.

TABLE OF CONTENTS

CONTACT DATA **2**
PARTICIPATING SITES **6**
TABLE OF CONTENTS **7**
PROTOCOL OUTLINE **10**
FLOW CHART **15**
ABBREVIATIONS **16**
1 INTRODUCTION **18**
1.1 CONVENTIONAL THERAPY 18
1.2 INDUCTION TREATMENT 18
1.3 CONSOLIDATION AND MAINTENANCE TREATMENT 20
1.4 BORTEZOMIB 21
1.4.1 Subcutaneous versus Intravenous Administration of Bortezomib *22*
1.5 LENALIDOMIDE 22
1.6 STUDY CONCEPTS WITH BORTEZOMIB AND LENALIDOMIDE 22
1.7 PROGNOSTIC FACTORS 23
1.7.1 Conventional Prognostic Factors *23*
1.7.2 Chromosomal Aberrations *23*
1.7.3 Array Comparative Genomic Hybridization (aCGH) *24*
1.7.4 Gene Expression Based Risk Assessment *24*
1.8 RATIONALE OF THE STUDY 24
1.9 BENEFIT-RISK-ASSESSMENT 25
1.10 REFERENCE COMMITTEES 26
2 TRIAL OBJECTIVES AND ENDPOINTS **27**
2.1 PRIMARY OBJECTIVES AND PRIMARY ENDPOINTS 27
2.2 SECONDARY OBJECTIVES AND SECONDARY ENDPOINTS 27
2.3 ADDITIONAL ANALYSES 28
3 SCIENTIFIC PROGRAMME **28**
3.1 DEPTH OF REMISSION – ON THE WAY TO AN OPERATIONAL CURE? 28
3.2 TOTAL MOLECULAR PROFILING OF MULTIPLE MYELOMA - RISK ASSESSMENT AND PATHOGENETIC MECHANISMS 29
3.2.1 iFISH *29*
3.2.2 Gene Expression Profiling *29*
3.2.3 Multicolour Flow Cytometry *30*
3.2.4 Free Light and Heavy Chain Test *30*
4 TRIAL DESIGN **31**
5 TRIAL DURATION AND SCHEDULE **31**
6 LENALIDOMIDE PREGNANCY PREVENTION PROGRAMME (PPP) **31**
6.1 PREGNANCY WARNING 31
6.2 CRITERIA FOR WOMEN OF NON-CHILDBEARING POTENTIAL 31
6.3 INCLUSION CRITERIA REGARDING CONTRACEPTION 32
6.4 PREGNANCIES REPORTING 33
7 SELECTION OF SUBJECTS **34**
7.1 NUMBER OF SUBJECTS 34
7.2 GENERAL CRITERIA FOR SUBJECTS' SELECTION 34
7.3 INCLUSION CRITERIA 34
7.4 EXCLUSION CRITERIA 35
7.5 RANDOMISATION AND STRATIFICATION 36
7.6 CRITERIA FOR WITHDRAWAL 36
7.7 PRIOR AND CONCOMITANT ILLNESSES 38
7.8 PRIOR AND CONCOMITANT TREATMENT 38

8 INVESTIGATIONAL MEDICINAL PRODUCT 39
8.1 GENERAL INFORMATION ABOUT STUDY MEDICATION BORTEZOMIB FOR INJECTION 39
8.1.1 Study Drug, Formulation, Strength, Quantity 40
8.1.2 Dosage, Administration 40
8.1.3 Labeling 40
8.2 GENERAL INFORMATION ABOUT STUDY MEDICATION LENALIDOMIDE 41
8.2.1 Clinical Pharmacology 41
8.2.2 Supplier(s) 42
8.2.3 Dosage Form 42
8.2.4 Packaging 42
8.2.5 Labeling 42
8.2.6 Receipt of Study Drug 42
8.2.7 Storage 42
8.2.8 Unused Study Drug Supplies 42
8.2.9 Compliance 43
8.3 KNOWN SIDE EFFECTS OF BORTEZOMIB AND LENALIDOMIDE 43
8.4 DOSAGE SCHEDULE OF BORTEZOMIB AND LENALIDOMIDE 43
8.5 TREATMENT ASSIGNMENT 43
9 TRIAL METHODS 43
9.1 TREATMENT SCHEDULE 43
9.1.1 General Aspects for all Patients 43
9.1.2 Induction Treatment 44
9.1.2.1 Induction Treatment with PAd (Arms A1 and B1) 44
9.1.2.2 Induction Treatment with with VCD (Arms A2 and B2) 44
9.1.2.3 Special Management in Conjunction with Bortezomib Therapy (All Patients) 44
9.1.2.4 Continuation after PAd 3 or VCD 3 46
9.1.3 Intensification regimen 46
9.1.4 Lenalidomide Consolidation (R) 47
9.1.4.1 Eligibility Criteria for R Consolidation 47
9.1.4.2 R Consolidation 47
9.1.4.3 Special Management Orders in Conjunction with R 48
9.1.5 Lenalidomide Maintenance Treatment 51
9.1.5.1 Eligibility Criteria for Lenalidomide Maintenance 51
9.1.5.2 Continous Maintenance Treatment with Lenalidomide (Arms A1 or A2) 51
9.1.5.3 Maintenance Treatment with Lenalidomide until CR (Arms B1 or B2) 51
9.1.5.4 Special Management Orders in Conjunction with Lenalidomide Maintenance (all patients) 52
9.1.6 Bisphosphonates 54
9.1.7 Platelet and Red Cell Transfusions 54
9.1.7.1 Guidelines for Platelet Transfusions 54
9.1.7.2 Guidelines for Red Cell Transfusions 55
9.2 METHODS OF DATA COLLECTION 55
9.3 REQUIRED INVESTIGATIONS AT ENTRY, DURING TREATMENT AND DURING FOLLOW UP 56
9.3.1 Clinical Investigations at Entry 56
9.3.2 Clinical Investigations during Treatment 58
9.3.2.1 Clinical Evaluations for Response Assessment 58
9.3.2.2 Additional Investigations during Treatment (Safety Parameter) 60
9.3.3 Central Diagnostic and Scientific Programme 60
9.4 EFFICACY PARAMETERS 62
9.5 SAFETY PARAMETERS 62
10 PLAN FOR TREATMENT OR CARE AFTER THE TRIAL 63
11 ADVERSE EVENTS 63
11.1 DEFINITIONS 63

11.1.1 Adverse Event ... *63*
11.1.2 Serious Adverse Event ... *64*
11.1.3 Adverse Drug Reaction ... *64*
11.1.4 Expectedness ... *64*
11.1.5 Suspected Unexpected Serious Adverse Reaction (SUSAR) ... *65*
11.1.6 Grading of AEs ... *65*
11.1.7 Relationship and Outcome of AEs ... *65*
11.2 PERIOD OF OBSERVATION AND DOCUMENTATION ... 66
11.3 REPORTING OF SERIOUS ADVERSE EVENTS BY INVESTIGATOR ... 68
11.4 EXPEDITED REPORTING ... 69
11.5 EMERGENCY TREATMENT ... 69
12 STATISTICAL PROCEDURES ... 69
12.1 SAMPLE SIZE CALCULATION ... 69
12.2 ANALYSIS VARIABLES ... 70
12.3 DEFINITION OF TRIAL POPULATION TO BE ANALYZED ... 71
12.4 STATISTICAL METHODS ... 71
12.5 INTERIM ANALYSES ... 73
12.6 PROTOCOL AMENDMENT No. 1 (protocol version 2.0) ... 73
12.7 PROTOCOL AMENDMENT No. 2 (protocol version 3.0) ... 74
13 DATA MANAGEMENT ... 74
13.1 DATA COLLECTION ... 74
13.2 DATA HANDLING ... 75
13.3 STORAGE AND ARCHIVING OF DATA ... 75
14 ETHICAL AND LEGAL ASPECTS ... 75
14.1 GOOD CLINICAL PRACTICE ... 75
14.2 SUBJECT INFORMATION AND INFORMED CONSENT ... 75
14.3 CONFIDENTIALITY ... 76
14.4 RESPONSIBILITIES OF INVESTIGATOR ... 76
14.5 APPROVAL OF TRIAL PROTOCOL AND AMENDMENTS ... 76
14.6 CONTINUOUS INFORMATION TO INDEPENDENT ETHICS COMMITTEE ... 77
14.7 NOTIFICATION OF REGULATORY AUTHORITIES ... 77
14.8 REGISTRATION OF THE TRIAL ... 77
14.9 INSURANCE ... 77
15 QUALITY ASSURANCE ... 78
15.1 MONITORING ... 78
15.2 INSPECTIONS/ AUDITS ... 78
16 AGREEMENTS ... 78
16.1 FINANCING OF THE TRIAL ... 78
16.2 FINANCIAL DISCLOSURE ... 78
16.3 REPORTS ... 78
16.4 PUBLICATION ... 78
17 SIGNATURES ... 80
18 DECLARATION OF INVESTIGATOR ... 81
19 APPENDICES ... 82
APPENDIX I: DIAGNOSTIC CRITERIA ... 83
APPENDIX II: RESPONSE CRITERIA ... 84
APPENDIX III: WHO PERFORMANCE STATUS AND NYHA CLASSIFICATION ... 87
APPENDIX IV: REQUIRED INVESTIGATIONS ... 88
APPENDIX V: CTC GRADING OF POLYNEUROPATHY ACCORDING TO CTCAE V4.0 ... 89
APPENDIX VI: MANAGEMENT OF PATIENTS WITH BORTEZOMIB (VELCADE®)-RELATED NEUROPATHIC PAIN AND/OR PERIPHERAL SENSORY NEUROPATHY ... 90
REFERENCE LIST ... 91

PROTOCOL OUTLINE

Title

Randomised phase III trial for previously untreated multiple myeloma to evaluate two regimens of bortezomib based induction therapy and lenalidomide consolidation followed by lenalidomide maintenance treatment (MM5)

Phase

III

Sponsor

University of Heidelberg
represented by the Director of Administration Irmtraut Gürkan
Im Neuenheimer Feld 672
69120 Heidelberg
Germany

Principal Investigator/ Coordinating Investigator (LKP)

Prof. Dr. med. Hartmut Goldschmidt
University of Heidelberg
Medical Clinic V – Department for Hematology, Oncology and Rheumatology
Im Neuenheimer Feld 410
69120 Heidelberg

Financing/ Status of the Sponsor

Non-commercial/ Co-financing and provision of study drug by pharmaceutical industry

Indication

Multiple Myeloma

Trial Population

<u>Inclusion Criteria</u>

Subjects meeting all of the following criteria will be considered for admission to the trial:

- Confirmed diagnosis of multiple myeloma requiring systemic therapy *(diagnostic criteria ("CRAB") see appendix I. For some patients systemic therapy may be required though CRAB criteria are not fulfilled. In this case the GMMG study office has to be consulted prior to inclusion.)*
- Measurable disease, defined as any quantifiable monoclonal protein value, defined by at least one of the following three measurements:[1]
  - Serum M-protein ≥ 10g/l
  - Urine light-chain (M-protein) of ≥ 200 mg/24 hours
  - Serum FLC assay: involved FLC level ≥ 10 mg/dl provided sFLC ratio is abnormal
- Age 18 - 70 years inclusive
- WHO performance status 0-3 (WHO=3 is allowed only when caused by MM and not by co-morbid conditions) *(see appendix IIIA)*
- Negative pregnancy test at inclusion (women of childbearing potential)
- For all men and women of childbearing potential: patients must be willing and capable to use adequate contraception during the complete therapy. Patients must agree on the requirements regarding the lenalidomide pregnancy prevention

programme described in chapter 6.

- All patients must
  - • agree to abstain from donating blood while taking lenalidomide and for one week following discontinuation of lenalidomide therapy
  - • agree not to share study drug lenalidomide with another person and to return all unused study drug to the investigator or pharmacist
- Ability of subject to understand character and individual consequences of clinical trial
- Written informed consent (must be available before enrollment in the trial)

Exclusion Criteria

Subjects presenting with any of the following criteria will not be included in the trial:

- Patient has known hypersensitivity to bortezomib, lenalidomide, dexamethasone, adriamycin and/or cyclophosphamide or to any of the constituent compounds (incl. boron and mannitol).
- Systemic AL amyloidosis (except for AL amyloidosis of the skin or the bone marrow)
- Previous chemotherapy or radiotherapy during the past 5 years except local radiotherapy in case of local myeloma progression. *(Note: patients may have received a cumulative dose of up to 160 mg of dexamethasone or equivalent as emergency therapy within 3 weeks prior to study entry.)*
- Severe cardiac dysfunction (NYHA classification III-IV*, see appendix IIIB*)
- Significant hepatic dysfunction (serum bilirubin ≥ 1,8mg/dl or ASAT and/or ALAT ≥ 2.5 times normal level), unless related to myeloma
- Patients known to be HIV-positive
- Patients with active, uncontrolled infections
- Patients with peripheral neuropathy or neuropathic pain, CTC grade 2 or higher (as defined by the NCI Common Terminology Criteria for Adverse Events (NCI CTCAE) Version 4.0, *see appendix V*)
- Patients with a history of active malignancy during the past 5 years with the exception of basal carcinoma of the skin or stage 0 cervical carcinoma
- Patients with acute diffuse infiltrative pulmonary and pericardial disease
- Autoimmune hemolytic anemia with positive Coombs test or immune thrombocytopenia
- Platelet count < 50 x 10^9/l (transfusion support within 14 days before the test is not allowed), unless related to myeloma
- Haemoglobin ≤ 7.5g/dl, unless related to myeloma
- Absolute neutrophil count (ANC) < 0.75 x 10^9/l (the use of colony stimulating factors within 14 days before the test is not allowed), unless related to myeloma
- Pregnancy and lactation
- Participation in other clinical trials. This does not include long-term follow-up periods without active drug treatment of previous studies during the last 6 months.

No subject will be allowed to enrol in this trial more than once.

Objectives

Primary Objectives

The MM5 trial is designed to address two independent primary objectives:

1.) Demonstration of non-inferiority of VCD induction therapy compared to PAd induction therapy with respect to response rate (very good partial remission or better; response criteria of the International Myeloma Working Group, IMWG).

2.) Determination of the best of four treatment strategies with respect to progression-free survival (PFS). The four treatment strategies are defined by PAd vs. VCD induction treatment, standard intensification therapy, lenalidomide consolidation and maintenance treatment with lenalidomide for 2 years vs. lenalidomide until CR.

Secondary Objectives

The secondary objectives of this trial are to determine and compare treatment arms with respect to

- overall survival rates (OS)
- response rates after lenalidomide consolidation treatment
- best response rates
- toxicity during induction treatment, lenalidomide consolidation and maintenance treatment with respect to adverse events of CTCAE grade $\geq$ 3

Additional Analyses

In additional analyses within this trial, the percentage of patients reaching a stringent (sCR) or molecular complete remission (mCR) and the prognostic value of depth of remission will be assessed (see section 3.1). As part of a "total molecular profiling" of myeloma (see section 3.2), the prognostic impact and interdependence on the respective treatment arm of chromosomal aberrations and gene expression profiles on PFS, OS, and on response to treatment will be evaluated.

Trial Design

Prospective, multicentre, randomised, parallel group, open, phase III clinical trial

Investigational Medicinal Product(s)/Study drugs

1.) Bortezomib (Velcade®)

2.) Lenalidomide (Revlimid®)

Sample Size

To be allocated to trial: n = 504, with the implementation of amendment 2 (protocol version 3.0) a further 100 patients are to be allocated for additional descriptive and explorative analyses

Estimated drop-out rate for PFS: 10%

Estimated rate of high risk patients leaving the trial for an experimental phase II trial: 5%

Statistical Analysis

Description of the Primary Efficacy Analysis and Population:

This trial has two primary efficacy analyses: (1) evaluating non-inferiority of VCD compared to PAd in induction treatment regarding response in a parallel two group design for Intent-to-Treat (ITT) and per-protocol (PP) population and (2) comparing the four treatment strategies A1,A2,B1,B2 for PFS in a parallel four group design for the ITT population

Efficacy Analysis for the Primary Objectives:

(1) Test the non-inferiority (NI) of VCD (A2+B2) compared to PAd (A1+B1) in response rates after induction therapy with a non-inferiority margin of 10% for the difference in response rates. Non-inferiority will be tested by comparing the two-sided confidence interval of difference to the NI margin.

(2) Closed test procedure to identify the most efficacious treatment strategy among A1,A2,B1,B2 with respect to PFS using log-rank tests in a closed multiple testing procedure, starting with the global null hypothesis of no difference between the four treatment arms.

The two objectives will be tested each at the 2-sided significance level 0.025 to achieve a total significance level of 0.05 for this trial.

Interim Analysis:

One interim analysis is planned to rule out lack of efficacy for the induction regimens. Results of the interim analysis will be presented confidentially to an independent data and safety monitoring board (DSMB). The interim analysis of response rates as defined for the first primary endpoint will be conducted after the first 75 patients in each induction regimen (A1+B1, A2+B2) are evaluable. Recruitment will be continued if the VGPR$^+$ rate exceeds 30%, i.e., if at least 23 of the first 75 randomised patients in each arm will reach a VGPR or better. At the same time, an interim analysis of the first primary endpoint will be carried out. The two-sided significance level of the first primary endpoint will be split into α=0.001 for the interim and α=0.024 for the final analysis.

An interim analysis of the second primary endpoint (PFS) will be performed at the same time the final analysis of the first primary endpoint is done which is approximately 42 months after start of recruitment. The two-sided significance level of the second primary endpoint will be split into α=0.001 for the interim and α=0.024 for the final analysis.

The study will be monitored closely based on the reported SAEs. Safety results will be provided to the DSMB on an annual basis as part of an interim safety report. One interim safety report will be provided at the same time as the interim analysis of the first primary endpoint.

As there are data showing a potentially increased risk for secondary primary malignancies (SPM) especially after alkylating agents (like Melphalan 200 or MPR) for patients during and after long term lenalidomide treatment[100,101,102], an additional close safety monitoring for secondary malignancies will be done. Any malignancy newly diagnosed during study treatment, has to be reported as serious adverse event, i.e. within 24h after awareness (see Chapter 11). In addition to the expedited SAE reporting, any newly diagnosed malignancy during study treatment and in the follow up period has to be documented in the eCRF (throughout the entire term of the study). A continuous safety monitoring with regard to SPMs will be described in an addendum to the statistical analysis plan. The monitoring will allow to stop the study early if the SPM rates exceed specific boundaries.

Sample Size:

(1) Assuming response rates after induction treatment (VGPR or better) of 46% for VCD and 42% for PAd, a total of 478 patients are required to demonstrate non-inferiority at a non-inferiority margin of 10% difference with 80% power at a one-sided significance level of α=0.0125. After accounting for 5% patients not being eligible for per-protocol population, 504 patients need to be enrolled. This sample size calculation is based on the method of Farrington and Manning

(2) Assuming 3 years of recruitment, 3 years minimal follow-up time, a total of 10% drop-outs and 5% high risk patients leaving the study prematurely after induction therapy, inclusion of 504 patients allows for rejecting the global null hypothesis of no difference between the four arms at the two-sided significance level of 0.025 with a power of 80%, if the arms achieve PFS rates of 75%, 65%, 65% and 55% after 3 years. This corresponds to hazard ratios relative to the best arm of 1.5, 1.5 and 2.1.

Secondary Endpoints: The secondary endpoints will be evaluated in an exploratory intention and without formal statistical correction for multiple testing.

High risk patients who leave the trial and receive an allogeneic transplantation will be censored for PFS and for OS at the date of transplantation.

Trial Duration and Dates

The duration of the trial for each subject is expected to be 35-38 months (induction and

intensification treatment: 6-9 months, 3 months rest between intensification and start of consolidation, consolidation 2 months, maintenance phase 24 months)

The overall duration of the trial is expected to be approximately 8 years including preparatory phase. Recruitment of subjects started in Q3 2010. The actual overall duration or recruitment may vary.

Total trial duration:	[96 months]
Duration of the clinical phase:	[74 months]
Beginning of the preparatory phase:	[Q4 2008]
FPI (First Patient In):	[Q3 2010]
LPI (Last Patient In):	[Q1 2014]
Analysis and publication of the first primary endpoint	[Q3 2014]
LPO (Last Patient Out):	[Q1 2017]
DBL (Data Base Lock):	[Q3 2017]
Statistical analyses completed:	[Q4 2017]
Trial report completed:	[Q1 2018]

Flow Chart

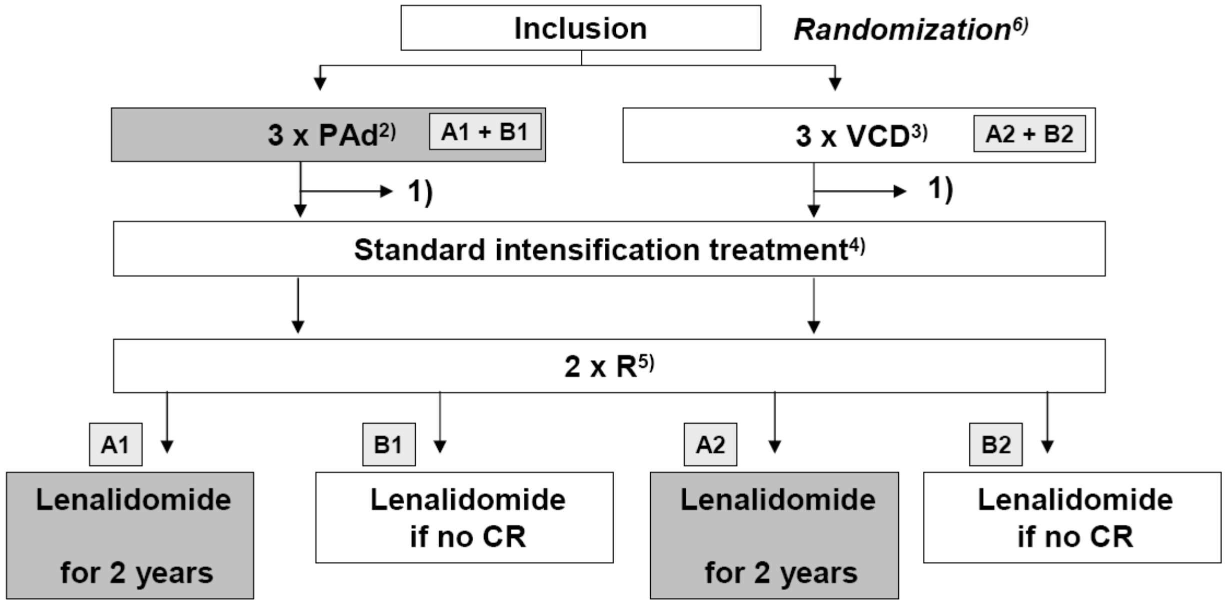

1) Risk assessment within first 4 weeks of therapy; high risk patients may go off protocol with participation in an experimental phase II trial (e.g., allogeneic transplantation)
2) PAd = Bortezomib, Adriamycin, Dexamethasone
3) VCD = Bortezomib, Cyclophosphamide, Dexamethasone
4) Standard intensification treatment according to local protocol
5) R = Lenalidomide
6) randomisation to one of four treatment strategies A1, B1, A2, B2: **A1**= PAd induction, lenalidomide maintenance for 2 years; **B1**= PAd induction, lenalidomide maintenance if no CR; **A2**= VCD induction, lenalidomide maintenance for 2 years; **B2** = VCD induction, lenalidomide maintenance if no CR

Abbreviations

AE.......... Adverse Event
aCGH..... Array Comparative Genomic Hybridization
ADL........ Activities of Daily Living
ADR........Adverse Drug Reaction
ALAT...... Alanine Amino Transferase, also known as SGPT
ANC........Absolute Neutrophil Count
ASAT......Aspartate Amino Transferase, also known as SGOT
ASH........American Society of Hematology
ASO-PCR...... Allele-specific Oligonucleotide Polymerase Chain Reaction
AMG....... German Drug Law (Deutsches Arzneimittelgesetz)
AUC........Area Under the Curve
B2M........Beta-2 Microglobulin
BfArM..... Bundesinstitut für Arzneimittel und Medizinprodukte
bFGF...... Basic Fibroblast Growth Factor
BMA....... Bone Marrow Aspiration
BSA........ Body Surface Area
CAD........Cyclophosphamide, Adriamycin, Dexamethasone
CD……....1. Cluster of Differentiation / Cluster of Designation 2. Compact Disc
CLcr....... Creatinine Clearance
C_{MAX}....... Peak Concentration
CR.......... Complete Response
CRF........ Case Report Form
CRP....... C-Reactive Protein
CT.......... Computed Tomography
CTC........ Common Toxicity Criteria
CTCAE... Common Terminology Criteria for Adverse Events
d............. Day
Dex......... Dexamethasone
DKFZ...... German Cancer Research Center (Deutsches Krebsforschungszentrum)
DSMB..... Data Safety Monitoring Board
DSMM.... German Myeloma Study Group (Deutsche Studiengruppe MM)
EBMT..... European Group for Blood and Bone Marrow Transplantation
EC.......... Ethics Committee
ECG....... Electrocardiogram
ECOG…. Eastern Cooperative Oncology Group
EFS........ Event Free Survival
e.g.......... For example (exempli gratia)
EMEA..... European Medicines Agency
EU.......... European Union
FACS...... Fluorescence Activated Cell Sorting
Fc........... Fragment crystallizable
FLC........ Free Light Chain
FPI.......... First Patient In
GCP....... Good Clinical Practice
GCP-V.... Good Clinical Practice Ordinance (GCP-Verordnung)
G-CSF.... Granulocyte-Colony Stimulating Factor
GEP........ Gene Expressing Profiling
GGT....... Gamma Glutamyl Transpeptidase
GIMEMA Italian Group for Adult Hematologic Diseases (Gruppo Italiano Malattie Ematologiche dell'Adulto)
GMMG.... German-Speaking Myeloma Multicenter Group
GMP....... Good Manufacturing Practice
GPI......... Gene Expression Based Proliferation Index
hCG........ Human Chorionic Gonadotrophin
HIV......... Human Immunodeficiency Virus
HLC........ "Hevylite" (Heavy/Light Chain)
HOVON.. Haemato Oncology Foundation for Adults in the Netherlands (stichting Hemato-Oncologie voor Volwassenen Nederland)
HR.......... High Risk
IB............ Investigator´s Brochure
ICH......... International Conference on Harmonization of Technical Requirements for Registration of Pharmaceuticals for Human Use
i.e. .... That is (id est)
iFISH...... Interphase Fluorescence in situ Hybridization
IFM......... Intergroup Francophone du Myelome
IFN......... Interferon
Ig............ Immunoglobulin
IIT........... Investigator Initiated Trial
IMP......... Investigational Medicinal Product
IMWG..... International Myeloma Working Group
ISF.......... Investigator Site File
ISRCTN.. International Standard Randomised Controlled Trial Number

ISS......... International Staging System
ITT.......... Intention To Treat
IUS......... Intrauterine System
IV............ Intravenous
KKS........ Coordination Center for Clinical Trials (Koordinierungszentrum für Klinische Studien)
LDH........ Lactatdehydrogenase
LKP........ Coordinating Investigator according to AMG (Leiter der Klinischen Prüfung)
LMWH.... Low Molecular Weight Heparin
LPI.......... Last Patient In
LPO........ Last Patient Out
LVEF...... Left Ventricular Ejection Fraction
mCR....... Molecular Complete Response
MDS....... Myelodysplastic Syndrome
miRNA.... Micro Ribonucleic Acid
MM......... Multiple Myeloma
MP.......... Melphalan Prednisone
MR......... Minimal Response
MRC....... Medical Research Council
MRD....... Minimal Residual Disease
MRI........ Magnetic Resonance Imaging
NC.......... No Change
NCI......... National Cancer Institute
nCR........ Near Complete Response
NCT....... National Center for Tumor Diseases
NI........... Non-inferiority
NR.......... No Response
NYHA..... New York Heart Association
ORR....... Overall Response Rate
OS.......... Overall Survival
PAd........ Bortezomib (PS-341, Velcade), Adriamycin, Dexamethasone
PD.......... Progressive Disease
PFS........ Progression-Free Survival
PI............ Principal Investigator
p.o.......... Per Os/ Orally
PP.......... Per Protocol
PPP........ Pregnancy Prevention Programme
PR.......... Partial Response
Q............ Quarter
R............. Revlimid (Lenalidomide)
R(A)D..... Revlimid, (Adriamycin,) Dexamethasone
RP.......... Revlimid, Prednisolon
RR.......... Reponse Rate
SADR..... Serious Adverse Drug Reaction
SAE........ Serious Adverse Event
SAP........ Statistical Analysis Plan
s.c........... Subcutaneous
sCR........ Stringent Complete Response
SD.......... Stable Disease
sFLC....... Serum Free Light Chain
SmPC..... Summary of Product Characteristics
SOP........ Standard Operating Procedure
S(P)EP... Serum (Protein) Electrophoresis
SUSAR... Suspected Unexpected Serious Adverse Reaction
T(A)D...... Thalidomide, (Adriamycin), Dexamethasone
Thal........ Thalidomide
TMF........ Trial Master File
TNF........ Tumor Necrosis Factor
TT........... “Total Therapy”
TTP........ Time To Progression
ULN........ Upper Limit of Normal
US.......... United States of America
Vc........... Velcade (Bortezomib)
VCD........ Bortezomib (Velcade, PS-341), Cyclophosphamide, Dexamethasone
VEGF..... Vascular Endothelial Growth Factor
VGPR..... Very Good Partial Response
VTD........ Velcade, Thalidomide, Dexamethasone
VTE........ Venous Thromboembolism
WHO...... World Health Organisation

1 Introduction

Multiple myeloma (MM) is a malignancy of plasma cells and is the second most common hematological malignancy. The incidence rate in Europe is 4-5/100,000 per year. Approximately 3,500 cases are diagnosed in Germany each year. Myeloma remains almost uniformly fatal. As the disease progresses, morbidity and eventual mortality are caused by impaired immune defences, skeletal destruction, anemia and renal failure. The use of intensified therapy has improved the remission rates in the last two decades. The 5-year survival rate for patients with myeloma treated with conventional chemotherapy was 25%, whereas intensified therapy increases this rate to up to 50 %.[2] The introduction of novel agents alongside conventional chemotherapy as well as their widespread use both upfront and in maintenance therapy after intensified therapy has further improved the prognosis of patients with MM.

1.1 Conventional Therapy

From 1962 until 2004/5, intermittent dosing of the combination of melphalan and prednisone (MP) was the treatment of choice. Despite many trials investigating different combinations of conventional chemotherapeutic agents during this time, none were shown to be associated with a significant improvement in overall survival compared to the original MP regimen. Between 50% and 60% of patients respond to conventional chemotherapy while only a minority (< 10%) of patients achieve a complete response. The median overall survival in these studies was less than 3 years. Superior efficacy with regard to both Progression-Free Survival (PFS) and Overall Survival (OS) has however been demonstrated by the addition of either bortezomib or thalidomide to the original MP regimen. Therefore, MP alone is no longer the standard of care in patients aged 65 years or older or for younger patients with contra-indications to intensified therapy. Phase I-II trial data suggest that adding lenalidomide to MP is also an effective treatment option. The first data from a phase III trial comparing MP versus MP and lenalidomide showed a highly significant prolonged PFS for patients treated with MP and lenalidomide followed by lenalidomide maintenance.[3] Therapies based on the combination of dexamethasone and novel agents have also been shown to prolong EFS and OS in MM patients.

1.2 Induction Treatment

For many years, VAD-like regimens (vincristine, adriamycin, and dexamethasone) were the standard induction chemotherapy for MM patients previous to an intensified treatment regime.[4] Although phase II studies showed overall response rates (ORRs) ranging from 55-60%, few patients achieved complete responses.[5;6] In general, patients induced with VAD required subsequent intensification before achieving CR. Recent efforts have focused on improving response rates, and in particular CR rates, by the use of novel agents in the upfront setting. A number of such studies involving thalidomide, bortezomib or lenalidomide are summarized below.

Initial induction studies confirmed the superiority of thalidomide and dexamethasone to VAD and dexamethasone alone in terms of the ORR. However, few patients achieved CR (7–13%).[7;8]

Thalidomide has also been investigated as a component of three-drug regimens. The HOVON50/GMMG-HD3 study investigated the use of thalidomide in combination with doxorubicin (adriamycin) and dexamethasone (TAD) in a randomised trial.[9] The TAD regimen resulted in significantly higher response rates compared to VAD: CR+VGPR 33% vs. 15% (*P*<0.001), ≥PR 72% vs. 54% (*P*<0.001).[5] In the HOVON-data analysis there was a significant improvement in EFS and PFS in the TAD arm: EFS 33 months vs. 22 months (*P*<0.001) and PFS 33 months vs. 25 months (*P*<0.001) for TAD vs. VAD, respectively. However, there was no

difference in overall survival between the two arms: 59 months for TAD vs. 62 months for VAD (*P*=0.96).[10] Similar results were found in the GMMG-HD3-trial (final study report of 04.12.2008).

The combination of bortezomib and dexamethasone as induction therapy has been examined by the French Myeloma Study Group (IFM) in 482 patients in a randomised phase III study.[11] In the IFM 2005/01 trial, patients were randomised to receive four cycles of VAD or four cycles of bortezomib and dexamethasone followed by intensified therapy. The results of this study demonstrated a significant advantage for bortezomib and dexamethasone compared to the VAD regimen: the ORR was 82% for bortezomib and dexamethasone as opposed to 65% for VAD (*P*<0.0001), with CR/nCR rates of 15% and 7%, respectively (*P*=0.0035) and CR + VGPR rates of 39% and 16%, respectively (P<0.0001). There was a significant difference between the two arms in the 2-year PFS (bortezomib and dexamethasone 69% vs. VAD 60%, *P*=0.0115), while 2-year OS was comparable in the two arms (90% vs. 88%, *P*=0.4689).

The phase III HOVON 65 MM/GMMG-HD4 trial is investigating PAD (bortezomib, adriamycin, dexamethasone) induction therapy in a randomised comparison with VAD followed by either bortezomib or thalidomide maintenance treatment post-intensification.[12] The PAD combination was significantly superior to VAD in terms of VGPR and PR rates. Following the first intensification, PAD was significantly superior to VAD in terms of CR/nCR, ≥VGPR and PR rates. Although the CR/nCR following PAD induction was unexpectedly low at 7%, it was found to increase during the course of treatment to 26% after the first intensification and to 43% as best response during bortezomib maintenance treatment. The combination of PAD was generally well tolerated and more than 80% of patients received the full planned course of PAD. There was no difference in hematological toxicities between VAD and PAD. PAD, however, was associated with a higher incidence of grade 3/4 polyneuropathy compared to VAD (16% vs. 6%).

The combination of bortezomib, cyclophosphamide and dexamethasone (VCD) has been found to be very active in the relapsed/refractory setting[13] and was also found to result in high response rates in a phase II study investigating this combination as induction therapy[14].

An phase II/III trial by the German DSMM study group is investigating VCD as induction regimen in 400 patients.[15] The results of an interim analysis involving 200 patients demonstrated an ORR of 84% and a CR rate of 12.5%. Response to treatment was found to be independent of the presence of ‘high-risk’ cytogenetic abnormalities. The combination was found to be well tolerated with a low overall incidence of neuropathy (12.5%) and a particularly low rate of severe neuropathy (grade 3 0.5%). Furthermore, a low mortality rate (1%) and a low risk of hospitalisation due to infection were detected.

A large phase III ECOG trial was investigating the use of lenalidomide in combination with two different doses of dexamethasone in the upfront setting. Patients are randomised to receive lenalidomide at 25 mg on days 1-21 and high-dose dexamethasone (40 mg days 1–4, 9–12, and 17–20 every 28 days [RD]) or low-dose dexamethasone (40 mg days 1, 8, 15, and 22 every 28 days [Rd]).[16] The primary aim of the study was to compare response to the two regimens after four cycles. The analysis revealed that the RD regimen was associated with a superior ORR and ≥VGPR-rate compared to Rd (ORR 79% vs. 68%, *P*=0.008; ≥VGPR 42% vs.24%, *P*<0.008). Best responses, including ORR (81% vs.70%; *P*=0.009) and ≥VGPR (51% vs.40%; *P*=0.04), were also significantly higher on the high-dose dexamethasone arm. Despite this difference in response to treatment the OS after a median follow up of 12.5 month was superior for patients treated with Rd. As a result of this observation the study was stopped by the independent data monitoring committee. The 1-year overall survival was 96% (95%, CI 94-99) in the Rd arm compared to 87% (95%, CI 82-92) in the RD arm.[17]. The 3-year OS was 75% in

both arms. Among patients who underwent intensified therapy after four cycles of primary treatment, 3-year OS was 92% compared with <60% in those patients who did not undergo intensified therapy.

The reduction of the dexamethasone dose in induction treatment in upcoming trials should be envisioned to decrease toxicity, especially infections, compared to induction regimens with high dose dexamethasone. Within the induction treatment in the GMMG-HD4-trial (German part of the GMMG-HD4/HOVON65 trial) there have been serious infections (fulfilling the criteria of a serious adverse event) in approx. 20 % of patients both in PAD and VAD. Fatal infections occurred in 3 of 4 deceased patients in PAD and 5 of 7 deceased patients in VAD.

1.3 Consolidation and Maintenance Treatment

Currently no guidelines exist concerning post-intensification therapy.[18;19] Thalidomide maintenance post-intensification has been investigated in a number of randomised trials which have led to different results. In two studies, thalidomide maintenance treatment was associated with a statistically significant improvement in PFS and OS.[20;21] For example, in the "Spencer-study" comparing prednisolone and thalidomide as opposed to prednisolone alone administered for 12 months following a single cycle of intensified treatment, the thalidomide-containing maintenance regimen resulted in significantly superior 3-year PFS (42% vs. 23%, P<0.001) and 3-year OS (86% vs. 75%, P=0.004) compared to prednisolone [21]. In addition, there was no significant difference in OS 12 months after disease progression (79% vs. 77%; P=0.237), indicating that thalidomide treatment did not result in a larger proportion of patients with resistant disease.

The addition of thalidomide in Total Therapy 2, which consisted of double intensification therapy with thalidomide given additionally from diagnosis until disease progression, did not prolong OS at a median follow up of 42 months.[22] Furthermore, in patients who received thalidomide, survival after relapse was significantly reduced compared to those who had not received thalidomide. However, with longer follow-up (median 72 months), survival in the thalidomide arm was found to be superior to that in the control arm. In patients with cytogenetic abnormalities, the difference was statistically significant.[23] An analysis of the effect of maintenance therapy in the MRC IX study revealed that thalidomide treatment was associated with a prolongation of PFS in patients who achieved less than VGPR post-induction indicating a consolidating rather than a maintenance effect.[24;25] No benefit in OS was observed due to early progress after relapse in those who had received thalidomide. It is also notable that thalidomide maintenance appeared to have a negative impact on survival in patients with del17p in one recent trial.[25;26]

A trial conducted by Ludwig et al. evaluated thalidomide and interferon (Thal-IFN) compared to interferon (IFN) alone in elderly patients who had previously undergone a first randomisation step to either Thal/Dex or MP induction therapy.[27;28] Of 289 patients who had been through the initial randomisation step, 135 achieved at least stable disease and were therefore eligible to undergo subsequent randomisation between the two maintenance arms. PFS was significantly longer with Thal-IFN maintenance treatment compared to IFN alone (24 vs. 12.6 months, P<0.024), but OS was similar in the two arms (52.6 vs. 52.2 months, P=0.68). Neurotoxicity, constipation and skin toxicity were significantly more frequent in the Thal-IFN group.[28]

In the HOVON50 and GMMG HD3 trial thalidomide 50 mg per day was given as maintenance after intensified therapy. In both trials PFS was prolonged and OS was not different in thalidomide containing induction and maintenance treatment arm.

In the HOVON-analysis[10] prognosis of patients developing relapses in the thalidomide maintenance-arm is inferior to patients treated with IFN. In summary, thalidomide after intensification treatment improves response and PFS. Results in terms of the impact of thalidomide maintenance on OS are different. The optimal duration and dosage of thalidomide after intensified chemotherapy is not known. Trials to compare thalidomide as consolidation versus thalidomide maintenance are necessary.

The role of bortezomib in the setting of maintenance and consolidation has been investigated in two small studies.[29;30] Preliminary data suggest that consolidation with VTD may induce molecular remission in some patients.[29] Ongoing randomised trials by several European study groups are further investigating the use of bortezomib as consolidation and maintenance therapy. For example, the German DSMM group is investigating the use of bortezomib as consolidation treatment following intensified therapy. The phase III GIMEMA trial also includes a randomised consolidation. Following induction treatment with VTD or TD and two cycles of intensification, patients are randomised to receive VTD or TD consolidation therapy. In the HOVON 65 MM/GMMG-HD4 trial, there is a comparison of bortezomib versus thalidomide maintenance therapy following the initial randomisation between PAD or VAD induction.

At the ASH Meeting 2009 Attal et al. presented data of the IFM 2005-02 trial.[31] In this randomised phase III study the consolidation with 25mg lenalidomide significantly improved the sCR/CR rate. The side effect profile of consolidation treatment with lenalidomide was low. 80% of the patients could receive the planned 2 cycles of consolidation.

1.4 Bortezomib

Bortezomib (VELCADE®, PS-341, JNJ-26866138) is a small molecule proteasome inhibitor which has been developed jointly by Millennium Pharmaceuticals, Inc. and Johnson & Johnson Pharmaceutical Research & Development. Bortezomib is a potent, reversible, and specific inhibitor of proteasomes and represents a first-in-class anti-neoplastic cytotoxic agent which shows a favorable side-effect profile when compared to conventional cytotoxic agents.

Non-clinical experience

Bortezomib has been studied extensively for its effect on various cellular functions requiring the ubiquitin-proteasome pathway in both in vitro and in vivo systems. A complete review of the non-clinical data is provided in the treating physician’s brochure.

Clinical experience

Bortezomib has been extensively studied in phase I and phase II trials, and efficacy in multiple myeloma has been demonstrated in randomised, open-label phase III studies.

Indication and usage in multiple myeloma

Currently bortezomib has got the EMEA approval as monotherapy for the treatment of progressive MM in patients who have received at least 1 prior therapy and who have already undergone or are unsuitable for intensified therapy. Additionally bortezomib is approved in combination with melphalan and prednisone (MPV) for the treatment of patients with previously untreated MM who are not eligible for intensified therapy. Meanwhile bortezomib was accepted as standard therapy for the first line treatment of MM by the Medical Services of the German Statutory Sickness Insurance (“Medizinischer Dienst der Krankenversicherungen”, MDK).

1.4.1 Subcutaneous versus Intravenous Administration of Bortezomib

A randomized phase III study to compare subcutaneous (sc) versus intravenous (iv) administration of bortezomib in patients with relapsed multiple myeloma was conducted by Moreau at al.[98] The results showed that subcutaneous bortezomib was non-inferior in terms of efficacy compared with intravenous administration (primary endpoint). The overall systemic availability and pharmacodynamic activity (area under the curve) were comparable with intravenous and subcutaneous administration. Importantly, the results of this study indicate that subcutaneous administration has an improved systemic safety profile compared with intravenous administration, notably resulting in significantly lower rates of peripheral neuropathy.

1.5 Lenalidomide

Lenalidomide (Revlimid®) belongs to a proprietary class of Celgene compounds called IMiDs® of which thalidomide is the parent compound. These drugs have both immunomodulatory and anti-angiogenic properties which could confer antitumor and antimetastatic effects.

Non-clinical experience

Lenalidomide has been demonstrated to possess anti-angiogenic activity through inhibition of bFGF, VEGF and TNF-alpha-induced endothelial cell migration, at least in part due to inhibition of Akt phosphorylation in response to bFGF. In addition, lenalidomide has a variety of immunomodulatory effects. Although the exact antitumor mechanism of action of lenalidomide is unknown, a number of pathways are postulated to be responsible for lenalidomide’s activity against multiple myeloma.

Clinical experience

Lenalidomide has been extensively studied in phase I and phase II trials, and the efficacy in multiple myeloma has been demonstrated in randomised, open-label phase III studies.

Indication and usage in multiple myeloma

In the US, the EU and in Switzerland lenalidomide is approved for the treatment of patients with multiple myeloma who have received at least one prior line of therapy.

1.6 Study Concepts with Bortezomib and Lenalidomide

Concerning the MM5 trial, first evidence for an increase of remission rates and depth of remission by a sequential use of PAD induction treatment, intensified therapy and lenalidomide consolidation/maintenance is given by data presented from Palumbo et al. at the ASH meeting 2008.[32] In this phase II trial the CR rate was increased to 73% during maintenance therapy (figure 1), being much higher compared to the result obtained in our HD4 trial by bortezomib-maintenance [CR/nCR rate of 41% during maintenance (intention to treat)][12].

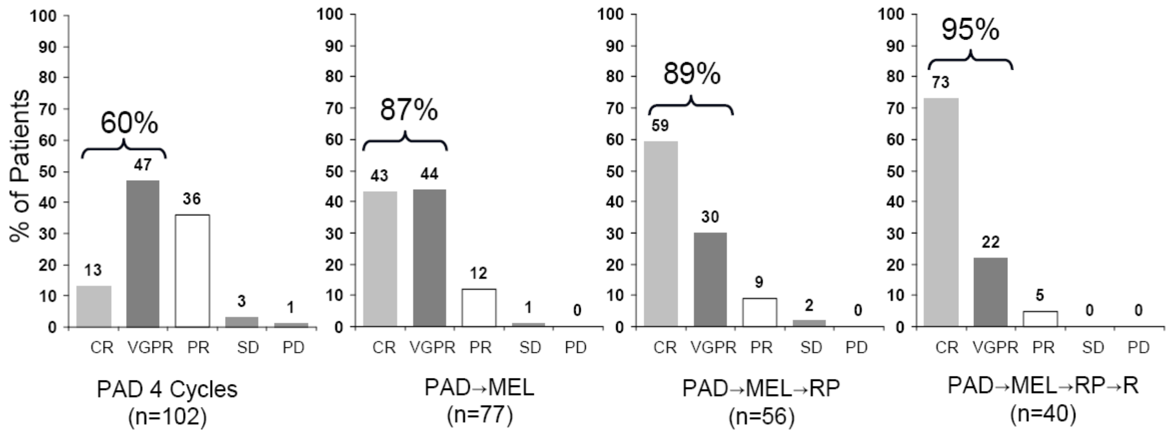

***Figure 1**: Trial: Bortezomib-Doxorubicin-Dexamethasone as Induction Prior to Reduced Intensification Followed by Lenalidomide as Consolidation/Maintenance in Elderly Untreated Myeloma Patients: Response rates (per protocol), adapted from Palumbo et al.* [32]

In the MM5 trial this treatment approach will be optimized. In the trial the VGPR+ (at least very good partial response) rate between the two induction regimens in a parallel two-arm design will be compared with a non-inferiority hypothesis. The second primary objective is to determine the best of four treatment arms (see flow chart on page 14) with regard to progression-free survival (PFS).

1.7 Prognostic Factors

1.7.1 Conventional Prognostic Factors

Several clinical adverse prognostic factors have been identified in multiple myeloma at diagnosis and before initiation of treatment.[33-37] The most significant include the international staging system (ISS) based on serum albumin[38] and the serum beta-2 microglobulin (B2M)[39-41]. B2M with different cut-off values remains a prognostic factor independent of addition of bortezomib (comparison of TT2 vs. TT3[42], see below) or lenalidomide to respective treatment schedules (RAD-trial, see below)[43].

1.7.2 Chromosomal Aberrations

Already the presence of an abnormal karyotype in metaphase cytogenetics or the detection of abnormal metaphases are associated with a shorter overall survival[44]. Karyotype cytogenetics analyses have limitations because results only are available if cells are proliferating.

FISH

iFISH allows the assessment of pre-selected chromosomal regions of known or assumed prognostic values and the assessment of the presence clonal or subclonal aberrations. Several chromosomal aberrations determined by iFISH show a prognostic relevance in univariate analysis. Of aberrations thought to be associated with pathogenesis of myeloma, (here: IgH-translocations) especially the translocation t(4;14) has shown to be of adverse prognostic value independent of conventional or intensified treatment with conventional agents, in either mutivariate or univariate analyses[44-49]. Several aberrations associated with disease progression, i.e., deletions of 17p13[44;45], gains of 1q21[45;50] or deletions of 13q14 are associated with adverse

prognosis in univariate analysis[44;45]. Different data are published regarding independence of these latter aberrations within multivariate analyses[45;51;52]; especially the prognostic relevance of deletions of 13q14.3 seem to not to be independent of t(4;14) or del17p[45;52].

Chromosomal aberrations as detected by iFISH (especially t(4;14) and del(17p) as well as +1q21 (>3 copies))[45;52,99] in combination with Beta2M[45] or the ISS score[99] give a strong characterisation of high-risk patients (see risk stratification, section 9.1.2.4).

Increasing evidence suggests that the impact of prognostic factors, notably t(4;14) and del17p are dependent on the treatment schedule, especially in terms of addition of bortezomib and lenalidomide. Barlogie et al. showed in a comparison of prognostic factors between total therapy 2 and 3, with one main difference between the treatment schedules being the addition of bortezomib in TT3, t(4;14) and del17p to be of adverse prognostic impact in TT2 only[42]. Additional support is given for an independence of bortezomib activity from these chromosomal aberrations by data obtained from the Vista-trial (Velcade Melphalan Prednisone), in which the TTP and OS did not show a significant difference in patients with presence or absence of either t(4;14), t(14;16) or del17p[53]. In terms of lenalidomide-treatment, del17p remains of adverse prognostic value within the MM-016 trial[54] or RAD (lenalidomide, adriamycin, dexamethasone)-treatment (in relapsed or refractory patients) in terms of PFS and OS[43].

As above stated data suggest an independence of EFS and OS from the presence of t(4;14) and del17p at least for bortezomib-treatment, but this has not been shown for schedule applied in this trial, patients with respective high risk signature can be optionally treated within a experimental phase II protocol outside this trial.

1.7.3 Array Comparative Genomic Hybridization (aCGH)

Array based comparative genomic hybridization (aCGH) is a complementary analysis to iFISH as it allows the assessment of copy number changes at 2.7 M chromosomal sites (Affymetrix 2.7M arrays). aCGH allows delineation of different pathogenetic[55] and prognostic groups[55;56]. aCGH, however, does not allow the determination of subclonal aberrations or balanced translocations. Subclones could be identified by iFISH.

1.7.4 Gene Expression Based Risk Assessment

Gene expression profiling (GEP) allowed to determine prognostic signatures that delineate high risk patients in current and experimental treatment protocols, including total therapy 2 and 3[57;58]. Proliferation of malignant plasma cells, as determined by several methods, has been shown to be a strong adverse prognostic factor[59-63], independent of clinical prognostic factors, e.g., B2M.[62] Gene expression based proliferation indices delineate patients with adverse survival[57;64;65]. In this trial, gene expression based assessment of proliferation will be performed.

1.8 Rationale of the Study

An intensification of myeloma treatment by including novel compounds in therapy concepts aims at higher rates of VGPR or CR[66-68] and thereby prolonging PFS[69]. Nevertheless, despite these better remission rates, the vast majority of patients still relapse. In order to further improve response to achieve long-term remission more than one novel compound with different mechanisms of action should be evaluated. As at least the majority of patients relapses after intensified treatment protocols, it is adamant to foresee the possibility of effective relapse treatment. The latter necessitates not having patients relapsing *under* continuous exposure of most highly effective compounds. One way to achieve this is the use of a sequential treatment schedule, i.e., subsequent application of not cross resistant treatment blocks.

The treatment concept within the MM5 trial is based on the use of non-cross resistant treatment blocks, i.e., i) bortezomib based induction treatment, ii) intensified therapy, and iii) lenalidomide based consolidation and maintenance treatment.

Combinations of bortezomib with either adriamycin or cyclophosphamide as induction regimen have been shown to reach a higher rate of remissions compared to standard VAD induction treatment (i.e., within the GMMG-HD4/HOVON 65 trial: PAD vs. VAD RR 83% vs. 59%, CR/nCR-rate 5% vs. 1%[12]). VCD, as tested in the DSMM XIa-trial, was shown to achieve comparable response rates to PAD (VCD RR 84% [15]). The reduction of the dexamethasone dose in PAd is envisioned to decrease toxicity, especially infections, compared to PAD. Within this trial the non-inferiority of VCD induction therapy compared to PAd induction therapy with respect to response rate shall be demonstrated.

Consolidation/Maintenance comprises the use of the IMiD derivate lenalidomide, not being cross resistant with bortezomib. The use of lenalidomide is included to i) allow a longer administration of maintenance treatment in a higher percentage of patients, and ii) because of an expected higher efficacy of lenalidomide compared to thalidomide. In this context, one of the research questions of the trial will be the comparison of two lenalidomide maintenance treatments. Lenalidomide maintenance until CR (arm B1 and B2) should be compared to lenalidomide maintenance for 2 years (arm A1 and A2) in terms of PFS and depth of response (rate of CR, sCR, mCR, VGPR).

First evidence for an increase of remission rates and depth of remission by a sequential use of PAD induction treatment, intensification and lenalidomid consolidation/maintenance treatment is given by data presented from Palumbo et al. at the ASH meeting 2008[32]. On the basis of the observed CR-rates of up to 73% (analyzed “as treated”) (figure 1), it is likely that a certain percentage of patients achieves a stringent or even molecular complete remission, i.e., 20% envisioned within this trial, as basis for a prolonged disease free survival.

Within this trial the best of four treatment strategies with respect to the progression-free survival shall be determined. The four treatment strategies differ in the induction therapy (PAd vs. VCD) and the duration of the lenalidomide based maintenance treatment (2 years vs. lenalidomide until CR).

1.9 Benefit Risk Assessment

Treatment

The therapy concept performed in the MM5 trial - including induction therapy, intensification therapy, consolidation and/or maintenance treatment - is standard of care in the first line treatment for patients suffering from multiple myeloma and being eligible for intensified chemotherapy up to the age of 65-70 years.

Within the trial, the “new compounds” bortezomib and lenalidomide are included in the therapy regimen. Both drugs have been shown to be effective in the treatment of multiple myeloma and have been approved by the EMEA for relapsed multiple myeloma.

Both bortezomib based induction regimens performed in the trial (PAd and VCD) as well as lenalidomide have previously been evaluated in phase II/III trials in multiple myeloma. Bortezomib and lenalidomide both have a well-defined safety profile and a favourable benefit–risk ratio in the treatment of multiple myeloma.

The most common side effects reported with bortezomib include fatigue, gastrointestinal adverse events, transient thrombocytopenia and neuropathy. The side effects most commonly

reported with lenalidomide are transient hematological toxicities (anemia, neutropenia, thrombocytopenia), fatigue, gastrointestinal and dermatological side effects. Lenalidomide is structurally related to thalidomide. Thalidomide is a known human teratogenic active substance that causes severe life-threatening birth defects. A teratogenic effect of lenalidomide cannot be ruled out.

It has been shown that response rates after bortezomib-based induction therapy are superior to those after former standard induction regimen VAD (vincristin, adriamycin, dexamethasone). Additionally there is first evidence for an increase of remission rates and depth of remission by a sequential use of bortezomib-based induction treatment, intensified therapy and lenalidomide consolidation/maintenance.

Treatment within the trial will be initiated and monitored under the supervision of physicians experienced in the treatment of multiple myeloma. Instructions are defined in the protocol to ensure the safety of the patients. If treatment related toxicities occur during the study, dose modifications of bortezomib and lenalidomide will be performed. A strict pregnancy prevention programme is included in the protocol, considering the use of lenalidomide in the trial and its potential teratogenic effects.

In conclusion, given the possible benefits of the treatment regarding improved response rate and survival and calculating the potential risks, the conduct of the study treatment is regarded as justifiable and there is no indication that patients are exposed to a non-justifiable risk associated with study participation.

Collection of blood and bone marrow samples

It is planned to collect additional blood and bone marrow samples within the trial. Dependent on the course for the individual patients up to 310 ml blood and 220 ml bone marrow aspirate will be collected during routinely performed blood samplings and bone marrow punctures and up to two additional bone marrow punctures during the whole study period, i.e. within a period of up to 3.25 years. The patients will be asked separately for their consent for the additional blood/bone marrow samples and also may participate in the trial if they refuse these scientific investigations.

Potential risks of blood and bone marrow samplings are well predictable and include rare and mostly mild complications such as vascular injury, reversible nerve irritation and/or bleeding. Considering a potential gain of relevant information about “depth” of response to the treatment and about a “molecular profiling” of multiple myeloma including prognostic factors for clinical outcome in patients with multiple myeloma, blood and bone marrow sample collection during the trial is considered to be highly justifiable.

1.10 Reference Committees

Data Safety Monitoring Board (DSMB)

An independent DSMB will be assembled. The DSMB will be composed of two independent experts in the field of myeloma and one biostatistician, assessing the progress, safety data and the interim analysis. The mission of the DSMB will be to ensure the ethical conduct of the trial and to protect the safety interests of patients in this trial.

The DSMB will meet (by teleconference) on a regular basis at least once a year. Based on its review the DSMB will provide the sponsor with recommendations regarding trial modification, continuation or termination.

The members of the DSMB are:

- **Professor Gösta Gahrton**, Department of Medicine, Karolinska University Hospital, Huddinge, Stockholm, Sweden

- **Professor Michel Attal**, Hématologie, Hôpital Purpan, Toulouse, France

- **Dr. Lutz Edler**, Biostatistician, Germany

The contact data of the members of the DSMB are listed on page 3 of this protocol. The responsibilities of the DSMB, frequency and format of meetings, communication procedures, etc. are described more detailed in an extra document ("MM5 - working procedures DSMB").

GMMG Advisory Board

The design, the objectives and the feasibility of the MM5 trial have been discussed within the GMMG advisory board before implementation. The members of the GMMG advisory board have received the protocol for review previous to submission to the ethics committee and regulatory authorities. Advice of the board will be sought in case of substantial amendments, if appropriate. The members of this board are listed on pages 3 and 4 of this protocol.

2 Trial Objectives and Endpoints

2.1 Primary Objectives and Primary Endpoints

Primary Objectives

The MM5 trial is designed to address two independent primary objectives. The primary objectives of the study are

1.) Demonstration of non-inferiority of VCD induction therapy compared to PAd induction therapy with respect to response rate (very good partial remission or better; response criteria of the International Myeloma Working Group, IMWG).

2.) Determination of the best of four treatment strategies with respect to progression-free survival (PFS). The four treatment strategies are defined by PAd vs. VCD induction treatment, standard intensification therapy, lenalidomide consolidation and maintenance treatment with lenalidomide for 2 years vs. lenalidomide until CR.

Primary Endpoints

1.) Response to treatment (very good partial remission or better) after induction therapy

2.) Progression-free survival (i.e., time from randomisation to progression or death from any cause whichever occurs first).

2.2 Secondary Objectives and Secondary Endpoints

Secondary Objectives

The secondary objectives of this trial are to determine and compare treatment arms with respect to

- overall survival rates (OS)
- response rates after lenalidomide consolidation treatment
- best response rates
- toxicity during induction treatment, lenalidomide consolidation and maintenance treatment with respect to adverse events of CTCAE grade $\geq$ 3

Secondary Endpoints

- Overall survival defined as time from randomisation to death from any cause. Patients still alive or lost to follow up are censored at the date they were last known to be alive.
- Response rates (response rates will be assessed using the following subcategories: SD, MR, PR, VGPR (with subgroup nCR), CR, sCR, mCR)
- Toxicity ((serious) adverse events CTC grade 3 and grade 4, CTC-AE v4.0)

2.3 Additional Analyses

In additional analyses within this trial, the percentage of patients reaching a stringent (sCR) or molecular complete remission (mCR) and the prognostic value of depth of remission will be assessed (see section 3.1). As part of a "total molecular profiling" of myeloma (see section 3.2), the prognostic impact and interdependence on the respective treatment arm of chromosomal aberrations and gene expression profiles on PFS, OS, and on response to treatment will be evaluated.

As described in chapter 12.6 and 12.7 the route of administration for Bortezomib was changed from intravenous to subcutaneous after implementation of a protocol amendment and additional 100 patients should be included. The additional recruitment will not affect the initially planned analysis of the primary and secondary endpoints of the trial. The trial will be analyzed as defined in the protocol based on the data of n=504 patients. After the data of n=604 patients are available, there will be an additional descriptive analysis of the safety profile and an additional exploratory analysis of the primary/secondary endpoints.

3 Scientific Programme

Overview. The scientific programme for the MM5 trial comprises three **main research areas**: 1) assessment of depth of remission, 2) "total molecular profiling" of myeloma, and 3) the assessment of myeloma bone disease and changes within the bone marrow microenvironment. Of these, assessment of depth of remission (minimal residual disease, MRD) and part of the molecular profiling (fluorescence-in situ hybridisation, gene expression profiling, free light and heavy chain test) are intergrated in the treatment protocol.

3.1 Depth of Remission – on the Way to an Operational Cure?

Scientific Question: Increasing Depth of Remission – the Way to an Operational Cure? Basic aim of the MM5 treatment schedule is to achieve a deeper remission tentatively leading to a longer PFS, and eventually being the prerequisite for a long term remission or operational cure. Thus, it is essential to assess accurately the remission state of all patients in accordance with the IMWG-response criteria[1] (see appendix II), as well as the presence of residual myeloma cells within the bone marrow (MRD-diagnostic, see algorithm in section 9.3.3).

Bone Marrow Aspiration for the Assessment of MRD. Reasoning. The underlying scientific questions is to what percentage the different arms within a sequential treatment with bortezomib based induction, followed by intensification treatment followed by lenalidomide maintenance, induce stringent (sCR) and molecular complete remissions (mCR). sCR is included in the new international response criteria[1] and can be assessed by flow cytometry[70]. Molecular complete remission (mCR) can be assessed by either flow cytometry (mCR^{FACS}) or ASO-PCR (mCR^{PCR}). The sensitivity is reported[71] to be between 1 malignant cell in 10^4 (flow cytometry) to $1:10^5$. All three parameters (sCR, mCR^{FACS}, mCR^{PCR}) will be prospectively evaluated in terms of being a prognostic factor for PFS and OS.

Assessment. MRD is assessed in a sequential approach if the patient achieves a complete remission with a normal κ/λ-ratio in the free light chain assay. First, 15 ml of whole bone marrow are subjected to the determination of κ/λ-ratio (for the determination of sCR) and mCR^{FACS} (assessment of percentage of malignant plasma cell using their individually determined phenotype (at diagnosis) by flow-cytometry including CD138/38/56/20/19/27/κ/λ-□staining□.[72;73] Second, if a patient reaches mCR^{FACS}, mCR^{PCR} will be performed using quantitative ASO-PCR.[74-77]

For the Algorithm of timepoints and criteria for MRD-assessment see section 9.3.2.1 and figure 2.

3.2 Total Molecular Profiling of Multiple Myeloma - Risk Assessment and Pathogenetic Mechanisms

To further delineate pathogenesis of myeloma, it is essential to concomitantly assess molecular changes in myeloma cells by a wide range of techniques. This "total molecular profiling" embedded and associated with this trial comprises: i) interphase fluorescence in situ hybridisation (iFISH) of CD138-purified myeloma cells, ii) array based comparative genomic hybridisation (aCGH), iii) gene expression profiling (GEP), iv) miRNA-profiling, v) kinome profiling, and vi) multicolour cytometry. Interphase-FISH, GEP and multicolour cytometry will be done within the trial for all patients as described below ("embedded investigations"). The further mentioned scientific investigations for the molecular profiling of myeloma (aCGH, miRNA-profiling, kinome profiling) as well as the investigation of single nucleotide polymorphisms, assessment of myeloma bone disease and treatment associated changes within the bone marrow are planned, but the actual realization (extent/timeframe) of these investigations is not yet definite and depends on the availability of aliquots of the existing samples and on further resources. For these potential "associated investigations" no additional samples will be taken from the patient. Additionally, patients are asked for their consent to keep remaining aliquots of the samples for potential future scientific investigations with respect to myeloma.

3.2.1 iFISH

Interphase FISH (iFISH) will be performed on CD138-purified myeloma cells using a comprehensive panel of probes, i.e., 1q21, 5p15, 5q31, 8p21, 9q34, 11q13, 11q23, 14q32, 15q22, 17p13, 19q13, IgH-breakeapart, as well as for the translocations t(4;14)(p16.3;q32.3), t(11;14)(q13;q32,3) and t(14;16). All iFISH results will be stored in a central database at the GMMG-study secretariat. As iFISH diagnostic is part of the routine diagnostic in myeloma, participating German centres are kindly requested to supply a referral form ("Überweisungsschein") when sending the BM-sample, and will receive a written report about the findings within 4 weeks. If no referral form is supplied, the iFISH results necessary for risk stratification (i.e., t(4;14) and del17p13) are available from the GMMG study office (contact see page 2).

3.2.2 Gene Expression Profiling

Gene expression profiling (GEP) will be performed for CD138-purified myeloma cells (using 100 ng of RNA), selected samples of the whole bone marrow microenvironment as well as in vitro differentiated osteoblast and osteoclasts by using Affymetrix U133 2.0 DNA-microarrays. GEP of $CD138^+$ myeloma cells allows molecular classification and risk stratification in terms of high-risk scores[57;58] and gene expression based assessment of proliferation[57;64;65]. GEP will be performed at inclusion in the trial, if a disease progression occurs during the trial, and at the end of the maintenance treatment phase. The first investigation is embedded in the trial. Prospective

evaluation of gene expression based risk assessment will be tested within this multicenter-trial. Aliquots and subfractions of the whole bone marrow will be stored for further analysis.

3.2.3 Multicolour Flow Cytometry

Multicolour flow cytometry is performed at diagnosis to assess the phenotype of the malignant plasma cell population. The respective phenotype is used for MRD-diagnostics (embedded, see above).

3.2.4 Free Light and Heavy Chain Test

Free Light Chain (FLC) Test (Freelite®). Immunoglobulin molecules consist of two identical heavy chains (A, D, E, G or M) which define the immunoglobulin class and two identical light chains (κ or λ). Each light chain is covalently linked to a heavy chain and the two heavy chains are linked covalently at the hinge region. In healthy individuals, the majority of light chain in serum exists in this form, bound to heavy chain. However, low levels of FLC are found in serum of normal individuals.

Freelite is a highly sensitive nephelometric or turbidimetric assay for quantification of immunoglobulin free light chains (FLC) in serum [78]. FLC assays are recommended for plasma cell dyscrasias at diagnosis in recent international guidelines and reports[79;80] and to assess stringent CR[1]. (See above.). An increase in FLC has been reported to be an early harbinger of relapse in multiple myeloma, especially in cases of *light chain escape*, a phenomenon observed in patients relapsing after intensive treatment[81]. In the MM5 trial, the FLC-assay is used to: i) assess sCR, ii) within the stepwise assessment of molecular complete remission (see section 3.1, and iii) to assess the ability of the FLC as indicator for early relapse and light chain escape.

Heavy chain (HLC) test (Hevylite®). Monoclonal immunoglobulins (see above) are currently identified and quantified from bands on electrophoretic gels. Several difficulties exist in obtaining precise measurements by electrophoretic methods, e.g., when an M-protein overlays a typical serum component,[82] and the method is to a certain degree investigator-dependant, of special hindrance in multicenter-trials. This is especially the case for the assessment of monoclonal IgA by SE, triggering many centres not to use the monoclonal but the total IgA as surrogate marker for disease activity. Furthermore, changes in blood volume, hematocrit and variable metabolism (e. g. IgG recycling via Fc receptors (FcRn)) may affect these measurements.

With the hevylite-test, M-protein can potentially be determined and quantified by measuring the separate light chain types of each immunoglobulin (Ig) class: Intact Ig molecules contain unique junctional epitopes between the heavy chain and light chain constant regions. These are the target of Hevylite (HLC) antibodies. Hence, they can separately identify the different light chain types of each immunoglobulin class, i.e., IgGκ, IgGλ, IgAκ, IgAλ, IgMκ and IgMλ. These molecules are then measured in pairs, e.g., IgGκ/IgGλ [78]. In terms of sensitivity, the HLC assays has shown a slightly lower sensitivity to detect monoclonal IgG but equal or higher sensitivity for IgA and IgM M-proteins compared to SPEP[83]. Direct measurement of the HLC fractions could thus allow detecting and measuring M-protein more reproducibly[84]. In theory, HLC κ/λ ratios should be unaffected by several of the variables influencing the immunglobulin-measurements stated above. In the assessment of residual disease, the HLC might be able to provide quantitative results for patients only being immunofixation positive. Additionally, HLC κ/λ ratios provide information about the tumour selective killing rates versus non-malignant plasma cell kill rates[83], which might also allow assessment of immunosuppression.

In the MM5 trial, the HLC-assay is assessed for the ability to: i) reproducibly identify the presence of and potentially quantify monoclonal immunoglobulins (especially IgA), ii) yield quantitative results in patients with VGPR/nCR and iii) assess possible differences in the killing of non-malignant plasma cells within the four treatment arms.

4 Trial Design

Prospective, multicentre, randomised, parallel group, open, phase III clinical trial.

There will be no blinding in this trial due to differences in the patient management in the treatment arms (different length of the induction cycles, differences in the frequency of intravenous drug applications).

5 Trial Duration and Schedule

The duration of the trial for each subject is expected to be 35-38 months (induction and intensification treatment: 6-9 months, 3 months rest between intensification and start of consolidation, consolidation 2 months, maintenance phase 24 months)

The overall duration of the trial is expected to be approximately 8 years including preparatory phase. Recruitment of subjects started in Q3 2010. The actual overall duration or recruitment may vary.

Total trial duration:	[96 months]
Duration of the clinical phase:	[74 months]
Beginning of the preparatory phase:	[Q4 2008]
FPI (First Patient In):	[Q3 2010]
LPI (Last Patient In):	[Q1 2014]
Analysis and publication of the first primary endpoint	[Q3 2014]
LPO (Last Patient Out):	[Q1 2017]
DBL (Data Base Lock):	[Q3 2017]
Statistical analyses completed:	[Q4 2017]
Trial report completed:	[Q1 2018]

6 Lenalidomide Pregnancy Prevention Programme (PPP)

6.1 Pregnancy Warning

Lenalidomide is structurally related to thalidomide. Thalidomide is a known human teratogenic active substance that causes severe life-threatening birth defects. Lenalidomide induced in monkeys malformations similar to those described with thalidomide. If lenalidomide is taken during pregnancy, a teratogenic effect of lenalidomide in humans is expected.

The conditions of the Pregnancy Prevention Programme must be fulfilled for all patients unless there is reliable evidence that the patient does not have childbearing potential.

6.2 Criteria for Women of Non-childbearing Potential

A female patient or a female partner of a male patient is considered to have childbearing potential unless she meets at least one of the following criteria:

- Age ≥ 50 years and naturally amenorrhoeic for ≥ 1 year. Amenorrhoea following cancer therapy does not rule out childbearing potential.

- Premature ovarian failure confirmed by a specialist gynaecologist
- Previous bilateral salpingo-oophorectomy, or hysterectomy
- XY genotype, Turner syndrome, uterine agenesis.

6.3 Inclusion Criteria regarding Contraception

Patients must meet all of the following criteria with respect to contraception to be eligible for enrollment into the study (complete inclusion and exclusion criteria for enrollment into the study see chapter 7.3 and 7.4):

- Female patients of **childbearing potential** must:
  - Understand the study drug lenalidomide is expected to have a teratogenic risk
  - Agree to use, and be able to comply with, effective contraception without interruption, 4 weeks before starting study drug lenalidomide, throughout the entire duration of study drug therapy (including dose interruptions) and for 4 weeks after the end of study drug therapy, even if she has amenorrhoea. This applies unless the subject commits to absolute and continued abstinence confirmed on a monthly basis. The following are effective methods of contraception:
    - Implant
    - Levonorgestrel-releasing intrauterine system (IUS)
    - Medroxyprogesterone acetate depot
    - Tubal sterilisation
    - Sexual intercourse with a vasectomised male partner only; vasectomy must be confirmed by two negative semen analyses
    - Ovulation inhibitory progesterone-only pills (i.e., desogestrel)

    If not established on effective contraception, the female subject must be referred to an appropriately trained health care professional for contraceptive advice in order that contraception can be initiated.

    Because of the increased risk of venous thromboembolism in patients with multiple myeloma taking lenalidomide and dexamethasone, combined oral contraceptive pills are not recommended. If a female subject is currently using combined oral contraception the patient should switch to one of the effective method listed above before starting lenalidomide treatment. The risk of venous thromboembolism continues for 4−6 weeks after discontinuing combined oral contraception. The efficacy of contraceptive steroids may be reduced during co-treatment with dexamethasone.

    Implants and levonorgestrel-releasing intrauterine systems are associated with an increased risk of infection at the time of insertion and irregular vaginal bleeding. Prophylactic antibiotics should be considered particularly in patients with neutropenia.

    Copper-releasing intrauterine devices are generally not recommended due to the potential risks of infection at the time of insertion and menstrual blood loss which may compromise patients with neutropenia or thrombocytopenia.
  - Understand that even if she has amenorrhea, she must follow all the advice on effective contraception.

- Understand the potential consequences of pregnancy and the need to rapidly consult the investigator if there is a risk of pregnancy.
- Agree to have a medically supervised pregnancy test with a minimum sensitivity of 25 mIU/ml on the day of the study visit or in the 3 days prior to the study visit once the subject has been on effective contraception for at least 4 weeks. This requirement also applies to women of childbearing potential who practice complete and continued abstinence. The test should ensure the subject is not pregnant when she starts treatment.
- Agree to have a medically supervised pregnancy test every 4 weeks including 4 weeks after the end of study treatment. These pregnancy tests should be performed on the day of the study visit or in the 3 days prior to the study visit. This requirement also applies to women of childbearing potential who practice complete and continued abstinence.

- **Male patients must**
  - Agree to use condoms throughout study drug therapy, during any dose interruption and for one week after cessation of study drug therapy if their partner is of childbearing potential.
  - Agree not to donate semen during study drug therapy and for one week after end of study drug therapy.

6.4 Pregnancies Reporting

Pregnancies and suspected pregnancies (including a positive pregnancy test regardless of age or disease state) of a female subject or the female partner of a male subject occurring while the subject is on study drug lenalidomide, or within 28 days of the subject's last dose of study drug lenalidomide, are considered events to be reported immediately to Sponsor and Celgene. If the subject is on study drug lenalidomide, study drug is to be discontinued immediately and the subject instructed to return any unused portion of the study drug to the investigator(s). The pregnancy, suspected pregnancy, or positive pregnancy test must be reported to Sponsor (fax to the KKS Heidelberg, fax-number: +49-(0)6221-56-33725) and to Celgene GmbH, Arzneimittelsicherheit, drugsafety-germany@celgene.com, Phone: (+49-(0)89-451 519 360) or Fax +49-(0)89-451 519-023 immediately by phone and facsimile using the SAE Reporting Form.

The exposure of any pregnant female (e.g., caregiver or pharmacist) to lenalidomide is also an immediately reportable event.

The female should be referred to an obstetrician/gynecologist experienced in reproductive toxicity for further evaluation and counseling.

The investigator(s) will follow the female subject until completion of the pregnancy, and must notify the Sponsor and Celgene GmbH, Arzneimittelsicherheit, drugsafety-germany@celgene.com or Fax +49-(0)89-451 519-023 of the outcome of the pregnancy (including notification of false-positive tests) within 24 hours of having knowledge of the event as a follow-up to the initial report.

If the outcome of the pregnancy meets the criteria for immediate classification as a SAE (i.e., spontaneous or therapeutic abortion [any congenital anomaly detected in an aborted fetus is to be documented], stillbirth, neonatal death, or congenital anomaly [including that in an aborted fetus]), the investigator(s) should follow the procedures for reporting SAEs (i.e., report the event to KKS Heidelberg, by Fax +49-(0)6221-56-33725 within 24 hours of the investigator's knowledge of the event).

All neonatal deaths that occur within 28 days of birth should be reported, without regard to causality, as SAEs. In addition, any infant death after 28 days that the Investigator(s) suspects is related to the in utero exposure to the study drug lenalidomide should also be reported to Sponsor within 24 hours of the Investigators' knowledge of the event.

If the female is found not to be pregnant, any determination regarding the subject's continued participation in the study will be determined by the investigator(s).

Male Subject

Female partners of males taking investigational product should be advised to call their healthcare provider immediately if they get pregnant. The male subject should notify the investigator of his partner's pregnancy and her healthcare provider information. If a pregnancy related event is reported in a female partner of a male subject, the investigator should ask if the female partner is willing to share information with Celgene Drug Safety and allow the pregnancy related event to be followed up to completion.The investigator will then provide this information to the Sponsor and Celgene for follow-up as necessary.

7 Selection of Subjects

7.1 Number of Subjects

As calculated in section 12.1 Sample Size Calculation, 504 subjects should be enrolled in the clinical trial. With the implementation of amendment 2 (protocol version 3.0) a further 100 patients are to be allocated for additional descriptive and explorative analyses. Recruitment and treatment of subjects should be performed in up to 40 main trial centers. Defined treatment periods may be performed in associated trials sites.

7.2 General Criteria for Subjects' Selection

Adult male or female patients up to the age of 70 years inclusive with newly diagnosed, symptomatic multiple myeloma will be randomised into the study.

Trial population will consist of both genders. Gender distribution in the trial is supposed to reflect the distribution in the real patient's population (approx. 60% male and 40% female patients), i.e. there will be no prior defined quantitative ratio between females and males.

7.3 Inclusion Criteria

Subjects meeting all of the following criteria will be considered for admission to the trial:

- Confirmed diagnosis of multiple myeloma requiring systemic therapy *(diagnostic criteria ("CRAB") see appendix I. For some patients systemic therapy may be required though CRAB criteria are not fulfilled. In this case the GMMG study office has to be consulted prior to inclusion.)*
- Measurable disease, defined as any quantifiable monoclonal protein value, defined by at least one of the following three measurements[1]:
  - Serum M-protein ≥ 10g/l
  - Urine light-chain (M-protein) of ≥ 200 mg/24 hours
  - Serum FLC assay: involved FLC level ≥ 10 mg/dl provided sFLC ratio is abnormal
- Age 18 - 70 years inclusive

- WHO performance status 0-3 (WHO=3 is allowed only when caused by MM and not by co-morbid conditions) *(see appendix IIIA)*
- Negative pregnancy test at inclusion (women of childbearing potential)
- For all men and women of childbearing potential: patients must be willing and capable to use adequate contraception during the complete therapy. Patients must agree on the requirements regarding the lenalidomide pregnancy prevention programme described in chapter 6.
- All patients must
  - agree to abstain from donating blood while taking lenalidomide and for one week following discontinuation of lenalidomide therapy.
  - agree not to share study drug lenalidomide with another person and to return all unused study drug to the investigator or pharmacist.
- Ability of subject to understand character and individual consequences of clinical trial
- Written informed consent (must be available before enrollment in the trial)

7.4 Exclusion Criteria

Subjects presenting with any of the following criteria will not be included in the trial:

- Patient has known hypersensitivity to bortezomib, lenalidomide, dexamethasone, adriamycin and/or cyclophosphamide or to any of the constituent compounds (incl. boron and mannitol).
- Systemic AL amyloidosis (except for AL amyloidosis of the skin or the bone marrow)
- Previous chemotherapy or radiotherapy during the past 5 years except local radiotherapy in case of local myeloma progression. *(Note: patients may have received a cumulative dose of up to 160 mg of dexamethasone or equivalent as emergency therapy within 3 weeks prior to study entry.)*
- Severe cardiac dysfunction (NYHA classification III-IV*, see appendix IIIB*)
- Significant hepatic dysfunction (serum bilirubin ≥ 1,8mg/dl or ASAT and/or ALAT ≥ 2.5 times normal level), unless related to myeloma
- Patients known to be HIV-positive
- Patients with active, uncontrolled infections
- Patients with peripheral neuropathy or neuropathic pain, CTC grade 2 or higher (as defined by the NCI Common Terminology Criteria for Adverse Events (NCI CTCAE) Version 4.0, *see appendix V*)
- Patients with a history of active malignancy during the past 5 years with the exception of basal carcinoma of the skin or stage 0 cervical carcinoma
- Patients with acute diffuse infiltrative pulmonary and pericardial disease
- Autoimmune hemolytic anemia with positive Coombs test or immune thrombocytopenia
- Platelet count < 50 x 10^9/l (transfusion support within 14 days before the test is not allowed), unless related to myeloma
- Haemoglobin ≤ 7.5g/dl, unless related to myeloma
- Absolute neutrophil count (ANC) < 0.75 x 10^9/l (the use of colony stimulating factors within 14 days before the test is not allowed), unless related to myeloma
- Pregnancy and lactation

- Participation in other clinical trials. This does not include long-term follow-up periods without active drug treatment of previous studies during the last 6 months.

No subject will be allowed to enrol in this trial more than once.

7.5 Randomisation and Stratification

The patient has to be registered before the start of chemotherapy. Patients need to be registered at the GMMG-Studiensekretariat by sending Case Report Form 1 ("Registration and Randomisation") by fax (Fax No.: +49-(0)6221-56-1957).

The following lab results already are necessary at registration, in addition to information regarding the eligibility criteria and the investigational site:

- Serum beta-2 microglobulin value
- Serum albumin value
- Serum M-protein (concentration of monoclonal protein in serum)
- Urine M-protein (Bence Jones)

All eligibility criteria will be checked with a checklist. ISS stage will be calculated from the provided serum beta-2 microglobulin value and serum albumin value. If the patient needs to be registered before the requested lab results are available, the GMMG study office has to be consulted in order that the patient can be included. The necessary laboratory investigations have to be initiated before start of treatment and the results have to be submitted to the GMMG study office as soon as possible.

Each patient will be given a unique patient study number ("randomisation number"). Patients will be randomised using block randomisation stratified by ISS stage in order to achieve a balance of treatment groups with respect to this prognostic covariate. There will be no additional stratification by center. Influence of this covariate is considered to be less because of the long trial experience of most centers within the GMMG.

The probability for assignment in each of the four treatment arms (A1, A2, B1, B2) is 25%, the relation of treatment arms is 1:1:1:1.

Patient study number and result of randomisation will be sent to the investigator by fax.

7.6 Criteria for Withdrawal

Withdrawal of Subjects

A subject may/ will be withdrawn from the trial <u>treatment</u> for the following reasons:

- at their own request or at request of the legal representative. Patients can withdraw from the study treatment at any time without having to give reasons for their decision. This will not result in any disadvantages for the patient.
- if, in the investigator's opinion, continuation of the trial would be detrimental to the subject's well-being
- for women, if it becomes known that the subject is pregnant
- major protocol violations
- intercurrent illness
- non-compliance of the patient
- confirmed progressive disease (PD) (after intensification, consolidation or during maintenance)

- non-eligibility for lenalidomide consolidation
- non-eligibility for maintenance treatment

It is recommended that the investigator contacts the Coordinating Investigator in order to reach a decision about withdrawal of subjects from trial treatment in case of doubt regarding any of the criteria mentioned.

In all cases, the reason for withdrawal must be recorded in the CRF and in the subject's medical records. In case of withdrawal of a subject at his/ her own request, the reason should be determined and documented. All examinations scheduled for the last trial day will be performed and documented as far as possible subject to the consent of the patient. These subjects will enter the regular follow up of the trial.

All treated subjects, even if they have discontinued treatment early, should enter the long term follow up period, unless the subject has withdrawn his/her consent to any further study-related procedure.

A subject may / will be withdrawn from all trial-related procedures (including follow-up visits) for the following reasons:

- at his/her own request or at request of his legal representative. Patients can withdraw from the study treatment at any time without having to give reasons for their decision. This will not result in any disadvantages for the patient.
- non-adherence to the trial-related requirements, which may (have) influence(d) the validity of the trial data

Replacement of Subjects

Subjects will not be replaced.

Premature Closure of the Clinical Trial

The whole study or one of the treatment arms can be interrupted or stopped by the principal investigator after considering benefits and risks. Reasons may be: unexpected serious adverse effects of treatment, excessive treatment-related mortality, new information from other studies or publications, inadequate recruitment, or excessive number of deviations from the protocol.

Should any of these occur, the principal investigator will notify the protocol committee of the trial, which will decide in due time on recommendation of interruption or termination of the study or of treatment arms. Before the decision a communication and an agreement with the DSMB will be sought.

Alternatively, the DSMB can recommend interruption or termination of the study or of treatment arms based on the results of the intermittent SAE evaluation or of accumulating information on the above mentioned reasons.

The ethics committee (EC) and the competent regulatory authorities must be informed about the premature closure of the trial or one of the treatment arms. Furthermore, the ethics committee(s) and competent regulatory authorities themselves may decide to stop or suspend the trial.

All involved investigators have to be informed immediately about a cessation / suspension of the trial. The decision is binding to all trial centers and investigators.

7.7 Prior and Concomitant Illnesses

Relevant additional illnesses present at the time of informed consent are regarded as concomitant illnesses and will be documented on the appropriate pages of the case report form (CRF). Included are conditions that are seasonal, cyclic, or intermittent (e.g., seasonal allergies; intermittent headache).

Abnormalities which appear for the first time or worsen (intensity, frequency) during the trial are adverse events (AEs) and must be documented on the appropriate pages of the CRF, if applicable (for AE documentation and exceptions see chapter 11).

7.8 Prior and Concomitant Treatments

The “study treatment” comprises induction chemotherapy (PAd and VCD), lenalidomide consolidation and lenalidomide maintenance treatment. Antineoplastic intensification therapy previous to the consolidation treatment should be done according to local standard protocols. This treatment has to be recorded in the CRF.

Concomitant medication, i.e., non-antineoplastic medication administered to the subjects on entry to the trial or at any time during the trial in addition to the study treatment, only is asked for in the CRF if the medication is recommended or mandatory according to the protocol (e.g., antibiotic prophylaxis, thrombosis prophylaxis, bisphosphonates) or in case of a serious adverse event (SAE). In case of an SAE the relevant concomitant medication at onset of the SAE has to be provided.

Additional concomitant medication (e.g., permanent medication with antihypertensive drugs, antiemetic drugs during chemotherapy) does not have to be recorded in the CRF.

Relevant additional myeloma-related treatments (e.g., radiotherapy) on entry to the trial or at any time during the trial are regarded as concomitant treatments and must be documented on the appropriate pages of the CRF.

Excluded Medication

The following concomitant treatments are not permitted during the trial:

- the use of steroids as permanent medication, other than < 10 mg/d prednisone or equivalent, is not allowed during the study (with the exception of dexamethasone being part of the protocol treatment).

The sponsor must be notified in advance (or as soon as possible thereafter) of any instances on which prohibited medications are administered.

Permitted Medications

Besides the “excluded medication” (see above) all medications and supportive therapies are permitted during the study according to the investigator’s assessment. In case of emergency treatment all necessary medications and procedures are permitted and no restrictions will be given.

Note: Localised radiation therapy is allowed, but the increased risk of leukocytopenia, erythrocytopenia and thrombocytopenia based on the combination of a polychemotherapy and radiation therapy has to be considered. In case of radiation therapy during induction treatment a close monitoring of the patients has to be assured.

8 Investigational Medicinal Product

8.1 General Information about Study Medication Bortezomib for Injection

8.1.1 Study Drug, Formulation, Strength, Quantity

The study drug bortezomib is commercially available and will be prescribed by the treating investigator in accordance with clinical practice and a socio-medical expertise of the Medical Services of the German Statutory Sickness Insurance (MDK) of 2009. Bortezomib for injection is a sterile lyophilized powder for reconstitution and is supplied in vials containing bortezomib and mannitol at a 1:10 ratio. For example, vials containing 3.5 mg of bortezomib contain 35 mg of mannitol.

The commercially available vials are sterile, single use vials containing 3.5 mg of bortezomib.

Since implementation of protocol version 2.0 (30.11.2011), bortezomib has to be administred subcutaneously to patients (see chapter 9.1.2Bortezomib is approved for intravenous as well as for subcutaneous administration. The differences regarding reconstitution for iv and sc use have to be noted, as described in the following.

Due to volume restrictions when administering products via the sc route, the volume of diluent required for reconstitution is lower than that used for IV use and the concentration of the diluted drug is higher (see below).

Each vial of bortezomib should be reconstituted under a laminar air flow biological cabinet (hood) within 24 hours before dosing with normal (0.9%) saline. Details for reconstitution volumes and the final concentration are shown in tables 1 and 2:

Table 1. Reconstitution of 3.5 mg bortezomib solution for SC injection

Route of administration	Pack size	Reconstitution volume	Final concentration
Subcutaneous use only	3.5 mg	1.4 ml	2.5 mg/ml

Dissolution is completed in approximately 10 seconds. The reconstituted solution is clear and colourless, with a final pH of 5 to 6. Parenteral drug products should be inspected visually for particulate matter and discoloration prior to administration whenever solution and container permit. If any articulate matter or discoloration is observed, the reconstituted product should not be used. Reconstituted bortezomib should be administered promptly and in no case more than 24 hours after reconstitution.

Bortezomib contains no antimicrobial preservative. When reconstituted as directed bortezomib may be stored at 25ºC. The reconstituted material may be stored up to 24 hours in the original vial or in a syringe. The total storage time for the reconstituted material must not exceed the 24 hours when exposed to normal indoor lighting.

Unopened vials may be stored at room controlled temperature of 25ºC, excursions permitted from 15º to 30ºC. Vials should be retained in the original package to protect from light. To date, stability data indicate that the lyophilized drug product is stable for at least 36 months when stored under the recommended conditions. Stability studies are ongoing, and the sponsor will notify the investigator should this information be revised during the conduct of the study.

Unopened vials of bortezomib are stable until the date indicated on the package if stored in the original package protected from light.

Bortezomib is a cytotoxic anticancer drug and, as with other potentially toxic compounds, caution should be exercised during handling and preparation. Proper aseptic technique should be used. Use of gloves and other protective clothing to prevent skin contact is recommended.

In case of skin contact, wash the affected area immediately and thoroughly with soap and water and diluted hydrogen peroxide. Remove contaminated clothing and dispose of according to standard procedures. In case of contact with mucous membranes, flush thoroughly with water. Always contact a physician after any form of body contact. All materials that have been used for preparation should be disposed of according to standard practices. A log must be kept of all disposed materials.

Please refer to the current investigator's brochure and the current SmPC for detailed information regarding the safety profile of bortezomib.

8.1.2 Dosage, Administration

Bortezomib will be administered only to eligible subjects. Bortezomib should be dispensed under the supervision of the investigator or a qualified physician. Subjects should be treated on an outpatient basis, if possible.

All patients receive bortezomib in a dose of 1.3 mg/m^2 body surface area (BSA) as described in chapter 9.1.2.

Bortezomib will be prepared under aseptic conditions. The actual dose (in mg) of bortezomib to be administered will be determined based on body surface area. BSA is to be calculated based on body weight and height using a standard nomogram or a formula. The dose should be calculated on day 1 of each cycle; the dose administered should remain the same throughout each cycle. If a subject experiences a notable change in weight the BSA and dose should be recalculated at that time. The height recorded during screening will be used for all BSA calculations.

Since implementation of protocol version 2.0 (30.11.2011), bortezomib has to be administred subcutaneously to patients (see chapter 9.1.2).

Subcutaneous Administration

Recommended subcutaneous injection sites are the thighs (proximal and distal sites) or abdomen (upper and lower quadrants); sites should be rotated for successive injections. Injections at the same site within a cycle should be avoided. Alternation between right and left abdomen, upper and lower quadrant, or right and left thigh, proximal and distal sites, are recommended.

8.1.3 Labeling

There is no specific labelling as an authorized and commercially available product is used in accordance with the accepted standard treatment.

8.2 General Information about Study Medication Lenalidomide

Revlimid® (lenalidomide), a thalidomide analogue, is an immunomodulatory agent with anti-angiogenic properties. The chemical name is 3-(4-amino-1-oxo 1,3-dihydro -2*H*-isoindol-2-yl) piperidine-2,6-dione. The empirical formula for lenalidomide is $C_{13}H_{13}N_3O_3$, and the gram molecular weight is 259.3.

Lenalidomide is an off-white to pale-yellow solid powder. It is soluble in organic solvent/water mixtures, and buffered aqueous solvents. Lenalidomide is more soluble in organic solvents and low pH solutions. Solubility was significantly lower in less acidic buffers, ranging from about 0.4 to 0.5 mg/ml. Lenalidomide has an asymmetric carbon atom and can exist as the optically active forms S(-) and R(+), and is produced as a racemic mixture with a net optical rotation of zero.

Revlimid® (lenalidomide) is available in 5 mg, 10 mg, 15 mg and 25 mg capsules for oral administration. Each capsule contains lenalidomide as the active ingredient and the following inactive ingredients: lactose anhydrous, microcrystalline cellulose, croscarmellose sodium, and magnesium stearate.

8.2.1 Clinical Pharmacology

Mechanism of Action

The mechanism of action of lenalidomide remains to be fully characterized. Lenalidomide possesses immunomodulatory and antiangiogenic properties. Lenalidomide inhibited the secretion of pro-inflammatory cytokines and increased the secretion of anti-inflammatory cytokines from peripheral blood mononuclear cells. Lenalidomide inhibited cell proliferation with varying effectiveness (IC50s) in some but not all cell lines. Of cell lines tested, lenalidomide was effective in inhibiting growth of Namalwa cells (a human B cell lymphoma cell line with a deletion of one chromosome 5) but was much less effective in inhibiting growth of KG-1 cells (human myeloblastic cell line, also with a deletion of one chromosome 5) and other cell lines without chromosome 5 deletions. Lenalidomide inhibited the expression of cyclooxygenase-2 (COX-2) but not COX-1 in vitro.

Pharmacokinetics and Drug Metabolism

Absorption

Lenalidomide, in healthy volunteers, is rapidly absorbed following oral administration with maximum plasma concentrations occurring between 0.625 and 1.5 hours post-dose. Co-administration with food does not alter the extent of absorption (AUC) but does reduce the maximal plasma concentration (Cmax) by 36%. The pharmacokinetic disposition of lenalidomide is linear. Cmax and AUC increase proportionately with increases in dose. Multiple dosing at the recommended dose-regimen does not result in drug accumulation.

Pharmacokinetic sampling in myelodysplastic syndrome (MDS) patients was not performed. In multiple myeloma patients maximum plasma concentrations occurred between 0.5 and 4.0 hours post-dose both on Days 1 and 28. AUC and Cmax values increase proportionally with dose following single and multiple doses. Exposure (AUC) in multiple myeloma patients is 57% higher than in healthy male volunteers.

Pharmacokinetic Parameters:

Distribution:

In vitro (^{14}C)-lenalidomide binding to plasma proteins is approximately 30%.

Metabolism and Excretion:

The metabolic profile of lenalidomide in humans has not been studied. In healthy volunteers, approximately two-thirds of lenalidomide is eliminated unchanged through urinary excretion. The process exceeds the glomerular filtration rate and therefore is partially or entirely active. Half-life of elimination is approximately 3 hours.

8.2.2 Supplier(s)

Celgene will supply Revlimid®, lenalidomide for the duration of this trial.

8.2.3 Dosage Form

Lenalidomide will be supplied as 5 mg, 10 mg, 15 mg and 25 mg capsules for oral administration.

8.2.4 Packaging

Drug will be shipped by InPhaSol (manufacturing site of the university hospital Heidelberg) to the investigator at the study site in individual wallets. Lenalidomide has to be ordered for the individual patient by the investigator who has to be registered at the GMMG study office. At least the principal investigators and deputy investigators of the German trial sites have to be registered in the "T-Register" at the BfArM. Wallets will contain a sufficient number of capsules to last for 21 days of dosing (for consolidation treatment) or for 28 days (for maintenance treatment), respectively. A maximum supply for 12 weeks - or for 4 weeks if the patient is a woman of childbearing potential - will be provided. Study drug must be dispensed in the original packaging with the label clearly visible. The central pharmacy at the site of the principle investigator ("Herstellbetrieb des Universitätsklinikums Heidelberg") is responsible for an accountable distribution of the study drug to other participating sites.

8.2.5 Labeling

Lenalidomide investigational supplies are dispensed to the patients in individual wallets of capsules. Each wallet will be labeled according to legal requirements. Samples of the labels are filed in the trial master file (TMF).

8.2.6 Receipt of Study Drug

The investigator or designee is responsible for taking an inventory of each shipment of study drug received, and comparing it with the accompanying study drug accountability form. The investigator will verify the accuracy of the information on the form, sign and date it, retain a copy in the study file, and return a copy to InPhaSol (manufacturing site of the university hospital Heidelberg).

8.2.7 Storage

At the study site, lenalidomide has to be stored in a locked, safe area to prevent unauthorized access.

The study drug should be stored at room temperature below 25°C and away from direct sunlight and protected from excessive heat and cold.

8.2.8 Unused Study Drug Supplies

Patients will be instructed to return wallets with unused capsules as well as empty wallets.

Unused capsules and empty wallets will be sent to the central pharmacy of the university hospital Heidelberg for disposition.

The sponsor is responsible for destroying any unopened wallets of drug, any partially used wallets of drug and empty wallets of drug.

The sponsor should provide Celgene Germany with a certificate of destruction in which the amount corresponds to the drug accountability log.

If any study drug is lost or damaged, its disposition should be documented in the source documents. Information should be forwarded to the GMMG study office immediately after awareness of loss.

8.2.9 Compliance

Lenalidomide will be dispensed to the subjects by the investigator. Subjects will be instructed to make entries in the trial diary for the lenalidomide intake. Compliance will be assessed by the entries in the patient's trial diary and by count of returned capsules by the "Herstellbetrieb des Universitätsklinikums Heidelberg" previous to destruction. Details will be recorded by the investigator in the CRF and the drug accountability log.

8.3 Known Side Effects of Bortezomib and Lenalidomide

For known side effects of bortezomib and lenalidomide reference is made to the current SmPC ("Fachinformation").

8.4 Dosage Schedule of Bortezomib and Lenalidomide

The dosage schedules of bortezomib and lenalidomide are described in chapter 9.

8.5 Treatment Assignment

The trial medication will be administered only to subjects included in this trial. It will be administered following the procedures set out in section 9 of the trial protocol.

Subjects withdrawn from the trial retain their identification codes (randomisation number). New subjects must always be allocated a new identification code.

9 Trial Methods

9.1 Treatment schedule

9.1.1 General Aspects for all Patients

- All men with female partners of childbearing potential and all women of child-bearing potential have to use contraception during the study. Please note that there are special instructions for contraception during lenalidomide consolidation and maintenance treatment (see section 6, lenalidomide pregnancy prevention programme). Sperm from men with child wish should be frozen before start of treatment.
- It is **mandatory** to give anti-viral and anti-bacterial prophylactic treatment to all patients:

  Recommended anti-viral prophylaxis:

  Aciclovir 2 x 400 mg/d p.o., starting on day 1 of PAd or VCD treatment and continuing until 4 weeks after end of PAd or VCD (i.e. 4 weeks after last dose of dexamethasone) for prophylaxis during induction treatment. Continuation of aciclovir prophylaxis is recommended for further 3 months.

Recommended anti-bacterial prophylaxis:

Cotrimoxazol 2 x 960 mg/d p.o., starting on day 1 of PAd or VCD treatment and continuing until 6 weeks after end of induction treatment.

Antif-fungal prophylaxis:

Anti-fungal prophylaxis may be performed according to local policy.

- Dosing of chemotherapy in obese patients: There is **no** general capping of the chemotherapy dose in obese patients recommended (e.g., with a maximum BSA of 2m²).

9.1.2 Induction Treatment

9.1.2.1 Induction Treatment with PAd (Arms A1 and B1)

Agent	Dose/day	Route	Days
Bortezomib	1.3 mg/m^2	subcutaneous	all cycles: days 1, 4, 8, 11
Adriamycin (Doxorubicin)	9 mg/m^2	i.v. rapid infusion	all cycles: days 1 – 4
Dexamethasone	20 mg	p.o.	all cycles: days 1 - 4, 9 - 12, 17 – 20

Cycle 2 will start at day 29, cycle 3 will start at day 57 (duration of each cycle = 28d).

9.1.2.2 Induction Treatment with VCD (Arms A2 and B2)

Agent	Dose/day	Route	Days
Bortezomib	1.3 mg/m^2	subcutaneous	all cycles: days 1, 4, 8, 11
Cyclophosphamide	900 mg/m^2	i.v.	all cycles: days 1
Dexamethasone	40 mg	p.o.	all cycles: days 1-2, 4-5, 8-9, 11-12

Cycle 2 will start at day 22, cycle 3 will start at day 43 (duration of each cycle = 21d).
In case of renal insufficiency cyclophosphamide should be reduced according to the creatinine clearance as follows:

Creatinine clearance	> 45 ml/min	45 – 10 ml/min	< 10 ml/min
Cyclophosphamide	100%	75%	50%

9.1.2.3 Special Management in Conjunction with Bortezomib Therapy (All Patients)

Patients may be treated on an outpatient basis.

Subcutaneous Administration Recommended subcutaneous injection sites are the thighs (proximal and distal sites) or abdomen (upper and lower quadrants); sites should be rotated for successive injections. Injections at the same site within a cycle should be avoided. Alternation between right and left abdomen, upper and lower quadrant, or right and left thigh, proximal and distal sites, are recommended.

Vials are for single use administration. The patient should be considered clinically stable by their physician before discharge.

Dose Adjustment of Bortezomib

Before each bortezomib dose, the patient will be evaluated for possible toxicities that may have occurred after the previous dose(s). All previously established or new toxicities observed any time, with the exception of neuropathic pain and peripheral sensory neuropathy for which separate guidelines are defined in appendix VI, are to be managed as follows:

Bortezomib doses should be withheld if the following events occur and are thought to be related to bortezomib:

- febrile neutropenia;
- grade 4 hematological toxicity;
- grade ≥ 3 non-hematological toxicity (excluding neuropathy, see below and appendix VI)

Febrile neutropenia

Bortezomib should be withheld until resolution of this condition, according to the judgement of the treating physician. After resolution, bortezomib may be restarted at a 25% reduced dose (at the discretion of the investigator)

Hematological toxicities

For grade 4 hematological toxicities, bortezomib is to be withheld for up to 2 weeks until the following grade 2 toxicity values are reached: hemoglobin >8.0 g/dl, ANC > 1.0 x 10^9/l, **and** platelet count > 50 x 10^9/l. Once cytopenias have resolved to at most grade 2, bortezomib should be restarted at a 25% reduced dose. Dose interruption or treatment discontinuation is not required for lymphopenia of any grade.

Non-hematological toxicities

For any grade ≥ 3 non-hematological toxicities, bortezomib is to be withheld for up to 4 weeks until the toxicity returns to at least grade 2. Bortezomib may then be restarted at a 25% reduced dose. If the toxicity does not resolve after dosing has been withheld for four weeks, the patient must be discontinued from treatment.

Dose adjustments after withholding bortezomib dosing for toxicities

If withholding the bortezomib dosing results in resolution of the toxicity, bortezomib may be restarted at a dose reduced by 25%, as follows:

- If the patient was receiving 1.3 mg/m^2, reduce the dose to 1.0 mg/m^2.
- If the patient was receiving 1.0 mg/m^2, reduce the dose to 0.7 mg/m^2.
- If the patient was receiving 0.7 mg/m^2, bortezomib must be discontinued.

It is at the discretion of the investigator if bortezomib will be restarted at full dose despite a previous toxicity grade ≥ 3, e.g. if the toxicity is manageable by supportive measures (e.g. in case of previous nausea and vomiting grade 3 that will be manageable by antiemetic treatment).

Neuropathic pain and/or peripheral sensory neuropathy

Patients who experience bortezomib related neuropathic pain and/or peripheral sensory neuropathy are to be managed as presented in the table in Appendix VI.

Dose Adjustment of Adriamycin, Cyclophosphamide and Dexamethasone

Dose reduction or withholding of cyclophosphamide, adriamycin and dexamethasone in case of toxicities during induction treatment is at the investigator's discretion according to standard medical care. After resolution of the toxicity therapy may be continued at the initial dose or a reduced dose of cyclophosphamide, adriamycin or dexamethasone, respectively.

9.1.2.4 Continuation after PAd 3 or VCD 3

Assessment of response after cycle 3 is described in chapter 9.3.2 and appendix II.

For all patients the concentration of beta-2 microglobulin (B2M) and albumin (ISS score)[99] will be assessed at inclusion and a FISH analysis is performed during the first 4 weeks of therapy. Patients are considered having "high risk" if the following criteria are fulfilled[99]:

presence of

- del 17p and/or
- t(4;14) and/or
- gain of 1q21 (>3 copies)

and (in combination with at least one of the above mentioned cytogenetic aberrations)

- ISS II/III

or patients suffering from plasma cell leukemia (independent of the combined FISH/ISS score)

These patients may go off protocol after induction therapy (after response evaluation) and be included in an experimental phase II trial e.g. evaluating allogeneic transplantation.

Patients with standard risk or patients with high risk opting not to leave the trial will continue with start of the intensification regime according to local protocols. This also holds for patients with progressive disease after PAd or VCD. Patients with new or progressive end-organ damage as a result of progressive myeloma disease should be treated with salvage therapy outside the MM5-trial.

For patients who do not meet the local inclusion criteria for the intensified therapy but who achieved a CR, VGPR, PR, MR or SD, it is strongly recommended to continue the treatment with further cycles PAd or VCD according to their randomisation arm. The PAd/VCD treatment should be continued until 2 cycles after a CR was reached or to a maximum of 8 cycles. Subsequently, the lenalidomide consolidation and maintenance treatment should be started according to the randomisation arm.

9.1.3 Intensification regimen

All eligible patients will be given an intensified therapy regime according to local standard protocols. For example, a commonly used regimen for intensified therapy is CAD followed by melphalan. Subsequent to the induction therapy, next treatment cycle should start

- between d28 and d42 after start of the third PAd cycle (arms A1 and B1) or
- between d21 and d35 after start of the third VCD cycle (arms A2 and B2).

Eligibility has to be assessed according to local standards. Recommended criteria are

- WHO performance 0-2
- Absence of severe pulmonary, neurologic, or psychiatric disease

- Bilirubin and transaminases of less than 2.5 times the upper limit of normal values

Anti-bacterial and anti-fungal prophylaxis may be performed according to local policy.

Assessment of response for the intensification cycles should be done as described in chapter 9.3.2 and appendix II.

9.1.4 Lenalidomide Consolidation (R)

All eligible patients will be given 2 cycles lenalidomide (R) starting 3 months after the start of the last cycle of intensification therapy.

9.1.4.1 Eligibility Criteria for R Consolidation

- All men with female partners of childbearing potential and all women of childbearing potential have to use adequate contraception 4 weeks before start of lenalidomide treatment, during lenalidomide treatment and four weeks thereafter (see section 6, lenalidomide pregnancy prevention programme)

For a new course of treatment beginning on the scheduled day 1 of the first or second cycle the following conditions must be met:

- Hematological recovery (ANC ≥ 1.0 x 10^9/l, platelets > 75 x 10^9/l)
- Before each cycle the creatinine clearance (CLcr) needs to be obtained by using the MDRD formula (needed parameter: serum creatinine, age, sex, ethnicity). The dose should be modified according to the table in 9.1.4.3.
- Any allergic reaction/hypersensitivity or sinus bradycardia/ other cardiac arrhythmia adverse event that may have occurred and was assessed as related to any of the study drugs has resolved to ≤ grade 1 severity;
- Any other adverse event that may have occurred and was assessed as related to any of the study drugs has resolved to ≤ grade 2 severity.
- If none of the criteria for permanent stopping of treatment have occurred (see recommendations regarding dose adjustment in case of toxicities in 9.1.4.3).
- If these conditions are not met on day 1 of a new cycle, the subject will be evaluated weekly and a new cycle will not be initiated until the toxicity has resolved as described above. If dosing was halted during the previous cycle and was restarted with a one-level dose reduction without requiring an interruption for the remainder of the cycle, then that reduced dose level will be initiated on day 1 of the new cycle. **If dosing was omitted for the remainder of the previous cycle or if the new cycle is delayed due to toxicity newly encountered on the scheduled day 1,** then the new cycle will be started with a one-level dose reduction for the study drug which has possibly caused the event.

9.1.4.2 R Consolidation

Agent	Dose/day	Route	Days
Lenalidomide	25 mg/d	p.o.	day 1-21

Cycle 2 will start at day 29.

Assessment of response after the 2nd course of R consolidation is described in chapter 9.3.1.1 and appendix II.

9.1.4.3 Special Management Orders in Conjunction with R

Venous Thromboembolism Prophylaxis

A venous thromboembolism (VTE) prophylaxis in conjunction with R consolidation is not recommended in general but has to be considered at investigator’s discretion. As Lenalidomide increases the risk of thrombotic events in patients who are at high risk or with a history of thrombosis, for these patients consideration should be given to the requirement or optional use of aspirin (100 mg) or some other form of prophylaxis as deemed appropriate.

Use in Patients with Impaired Renal Function

Lenalidomide is substantially excreted by the kidney, therefore care should be taken in dose selection and monitoring of renal function is advised.

No dose adjustments are required for patients with mild renal impairment. The following dose adjustments are recommended at the start of therapy for patients with moderate or severe impaired renal function or end stage renal disease.

Renal Function (CLcr)	Dose Adjustment
Moderate renal impairment<br>(30 ≤ CLcr < 50 ml/min)	10 mg once daily
Severe renal impairment<br>(CLcr < 30 ml/min, not requiring dialysis)	15 mg every other day*
End Stage Renal Disease (ESRD)<br>(CLcr < 30 ml/min, requiring dialysis)	5 mg once daily. On dialysis days, the dose should be administered following dialysis.

* The dose may be escalated to 10 mg once daily if the patient is tolerating the treatment

Recommended Dose Adjustments during Treatment and Restart of Treatment

Dose adjustments, as summarised below, are recommended to manage grade 3 or 4 neutropenia or thrombocytopenia, or other grade 3 or 4 toxicity judged to be related to lenalidomide.

- *Dose Reduction Steps*

Starting dose	25 mg
Dose level 1	15 mg
Dose level 2	10 mg
Dose level 3	5 mg

- *Absolute Neutrophil Counts (ANC), Neutropenia*

When neutrophils	Recommended Course
First fall to < 0.5 x 10^9/l<br><br>Return to ≥ 0.5 x 10^9/l when neutropenia is the only observed toxicity	Interrupt lenalidomide treatment, give G-CSF (lenograstim 150 µg/m²/d)<br>Resume lenalidomide at starting dose once daily
Return to ≥ 0.5 x 10^9/l when dose-dependent hematological toxicities other than neutropenia are observed	Resume lenalidomide at dose level 1 once daily
For each subsequent drop below < 0.5 x 10^9/l<br><br>Return to ≥ 0.5 x 10^9/l	Interrupt lenalidomide treatment, give G-CSF (lenograstim 150 µg/m²/d)<br>Resume lenalidomide at next lower dose level (dose level 1,2 or 3) once daily. Do not dose below 5 mg once daily.

- *Platelet Counts, Thrombocytopenia*

When platelets	Recommended Course
First fall to < 25 x 10^9/l<br>Return to ≥ 25 x 10^9/l	Interrupt lenalidomide treatment<br>Resume lenalidomide at dose level 1
For each subsequent drop below 25 x 10^9/l<br>Return to ≥ 25 x 10^9/l	Interrupt lenalidomide treatment<br><br>Resume lenalidomide at next lower dose level (dose level 2 or 3) once daily. Do not dose below 5 mg once daily.

- *Non-hematological Toxicities*

see next page

- Non-hematological Toxicities

CTC Toxicity Grade	Day 2-14 of Cycle	≥Day 15 of Cycle
**Non-blistering rash Grade 3**	• If grade 3 hold (interrupt) dose. Follow weekly.<br>• If the toxicity resolves to ≤ grade 1 prior to d 21 restart at next lower dose level and continue the cycle until d 21.	• Omit lenalidomide for remainder of cycle.
**Grade 4**	• Discontinue lenalidomide study drug.	• Discontinue lenalidomide study drug.
**Desquamating (blistering) rash- any Grade**	• Discontinue lenalidomide study drug.	• Discontinue lenalidomide study drug.
**Erythema multi-forme ≥ Grade 3**	• Discontinue lenalidomide study drug.	• Discontinue lenalidomide study drug.
**Sinus bradycardia/ other cardiac arrhythmia Grade 2**	• Hold (interrupt) dose. Follow at least weekly.<br>• If the toxicity resolves to ≤ grade 1 prior to d 21 restart at next lower dose level and continue the cycle until d 21.	• Omit lenalidomide for the remainder of the cycle.
**≥ Grade 3**	• Discontinue lenalidomide study drug.	• Discontinue lenalidomide study drug.
**Neuropathy Grade 3**	• Interrupt lenalidomid treatment.<br>• If the toxicity resolves to ≤ grade 1 prior to d 21 restart at next lower dose level and continue the cycle until d 21.	• Omit lenalidomide for the remainder of the cycle.
**Grade 4**	• Discontinue lenalidomide study drug.	• Discontinue lenalidomide study drug.
**Allergic reaction or hypersensitivity Grade 2-3**	• Hold (interrupt) dose. Follow at least weekly.<br>• If the toxicity resolves to ≤ grade 1 prior to d 21 restart at next lower dose level and continue the cycle until d 21.	• Omit lenalidomide for the remainder of the cycle.
**Grade 4**	• Discontinue lenalidomide study drug.	• Discontinue lenalidomide study drug
**Venous thrombosis/ embolism ≥ Grade 3**	• Hold (interrupt) dose and start anticoagulation; restart at investigator's discretion (maintain dose level).	• Omit lenalidomide for remainder of cycle.
**Hyperthyroidism or hypothyroidism**	• Omit lenalidomide for remainder of cycle, evaluate etiology, and initiate appropriate therapy. Restart lenalidomide next cycle (decrease dose by one dose level).	• Omit lenalidomide for remainder of cycle, evaluate etiology, and initiate appropriate therapy. Restart lenalidomide next cycle (decrease dose by one dose level).
**Other non-hematologic toxicity assessed as Lenalidomide-related ≥ Grade 3**	• Hold (interrupt) dose. Follow at least weekly.<br>• If the toxicity resolves to ≤ grade 2 prior to d 21 restart at next lower dose level and continue the cycle until d 21.	• Omit lenalidomide for remainder of cycle.

9.1.5 Lenalidomide Maintenance Treatment

9.1.5.1 Eligibility Criteria for Lenalidomide Maintenance

- All men and all women of childbearing potential have to use adequate contraception 4 weeks before start of lenalidomide treatment, during lenalidomide treatment and four weeks thereafter (see section 6, lenalidomide pregnancy prevention programme)

For beginning of maintenance treatment and thereafter for continuation of the treatment (i.e. beginning of a new maintenance “period” after the regular study visits) the following conditions must be met:

- Hematological recovery (ANC ≥ 1.0 x 10^9/l, platelets > 75 x 10^9/l)
- At the study visits the creatinine clearance (Cr_{CL}) needs to be obtained by using the MDRD formula (needed parameter: serum creatinine, age, sex, ethnicity). The dose should be modified according to the table in 9.1.5.4.
- Any allergic reaction/hypersensitivity or sinus bradycardia/ other cardiac arrhythmia adverse event that may have occurred and was assessed as related to any of the study drugs has resolved to ≤ grade 1 severity;
- Any other adverse event that may have occurred and was assessed as related to the study drugs has resolved to ≤ grade 2 severity.
- If none of the criteria for permanent stopping of treatment have occurred (see recommendations regarding dose adjustment in case of toxicities in 9.1.5.4).
- If these conditions are not met on the study visit, the subject will be evaluated weekly and resumption of maintenance will not be initiated until the toxicity has resolved as described above. If dosing was halted during the previous maintenance period and was restarted with a one-level dose reduction without requiring an interruption for the remainder of the period, then that reduced dose level will be initiated on day 1 of the new period. **If dosing was omitted for the remainder of the previous period or if the new period is delayed due to toxicity newly encountered on the scheduled day 1,** then the new period will be started with a one-level dose reduction for the study drug which has possibly caused the event.

9.1.5.2 Continous Maintenance Treatment with Lenalidomide (Arms A1 or A2)

Patients randomised to arm A1 or A2 will continue with lenalidomide after the last course of R consolidation, independent of the response (not in case of progressive disease). The dose of maintenance lenalidomide is 10 mg/d within the first 3 months of maintenance treatment. Subsequently, if the above mentioned eligibility criteria for lenalidomide maintenance are fulfilled, the lenalidomide dose should be increased to 15mg/d. In arms A1 and A2 lenalidomide maintenance will be continued for 2 years or until disease progression.

Repeated assessment of response during lenalidomide maintenance is described in chapter 9.3.2 and appendix II.

9.1.5.3 Maintenance Treatment with Lenalidomide until CR (Arms B1 or B2)

Patients randomised to arm B1 or B2 will continue with lenalidomide after the last course of R consolidation, in case they have not achieved a CR. The dose of maintenance lenalidomide is 10 mg/d within the first 3 months of maintenance treatment. Subsequently if the above mentioned eligibility criteria for lenalidomide maintenance are fulfilled the lenalidomide dose should be increased to 15mg/d.

In arms B1 and B2 lenalidomide maintenance will be continued until the patient achieves a CR (lenalidomide is given until confirmation of CR. Confirmation of CR should be performed at a time of 4-6 weeks.) In case no CR will be achieved, lenalidomide will be given for 2 years or until disease progression.

Repeated assessment of response during lenalidomide maintenance is described in chapter 9.3.2 and appendix II.

9.1.5.4 Special Management Orders in Conjunction with Lenalidomide Maintenance (all patients)

Venous Thromboembolism Prophylaxis

During maintenance treatment with lenalidomide no general venous thromboembolism (VTE) prophylaxis is recommended.

As Lenalidomide increases the risk of thrombotic events in patients who are at high risk or with a history of thrombosis, for these patients consideration should be given to the requirement or optional use of aspirin (100 mg) or some other form of prophylaxis as deemed appropriate

Lenalidomide Maintenance Treatment in Patients with impaired Renal Function

No dose adjustments are required for patients with mild renal impairment. The following dose adjustments are recommended at the start of therapy for patients with moderate or severe impaired renal function or end stage renal disease.

Renal Function (CLcr)	Dose Adjustment
Moderate renal impairment<br>(30 ≤ CLcr < 50 ml/min)	5 mg once daily*
Severe renal impairment<br>(CLcr < 30 ml/min, not requiring dialysis)	5 mg every other day**
End Stage Renal Disease (ESRD)<br>(CLcr < 30 ml/min, requiring dialysis)	5 mg three times weekly.<br>On dialysis days, the dose should be administered following dialysis.

* The dose may be escalated to 10 mg once daily if the patient is tolerating the treatment (no hematological toxicity)

** The dose may be escalated to 5 mg once daily if the patient is tolerating the treatment (no hematological toxicity)

Recommended Dose Adjustments during Treatment and Restart of Treatment

Dose adjustments, as summarised below, are recommended to manage grade 4 neutropenia or thrombocytopenia, or other grade 3 or 4 toxicity judged to be related to lenalidomide.

- Dose Reduction Steps

Starting dose	10 mg<br>(first 3 months)	15 mg<br>(4th month et seq.)
Dose level 1	5 mg	10 mg
Dose level 2	-	5 mg

- Absolute Neutrophil Counts (ANC), Neutropenia

**Current dose level 15 mg**	
**When neutrophils**	**Recommended Course**
Fall to < 0.5 x 10^9/l (CTC grade 4)	Interrupt lenalidomide treatment, give G-CSF (lenograstim 150 µg/m²/d)
Return to ≥ 0.5 x 10^9/l	Resume lenalidomide at a dose of 10 mg/d
**Current dose level 10 mg**	
**When neutrophils**	**Recommended Course**
Fall to < 0.5 x 10^9/l	Interrupt lenalidomide treatment, give G-CSF (lenograstim 150 µg/m²/d)
Return to ≥ 0.5 x 10^9/l	Resume lenalidomide at a dose of 5 mg/d
**Current dose level 5mg**	
**When neutrophils**	**Recommended Course**
Fall to < 0.5 x 10^9/l	Interrupt lenalidomide treatment, give G-CSF (lenograstim 150 µg/m²/d)
Return to ≥ 0.5 x 10^9/l	Resume lenalidomide at a dose of 5 mg/d
For a subsequent drop below 0.5 x 10^9/l	Discontinue lenalidomide study drug.

- *Platelet Counts, Thrombocytopenia*

**Current dose level 15 mg**	
**When platelets**	**Recommended Course**
Fall to < 25 x 10^9/l	Interrupt lenalidomide treatment
Return to ≥ 25 x 10^9/l	Resume lenalidomide at a dose of 10 mg/d
**Current dose level 10 mg**	
**When platelets**	**Recommended Course**
Fall to < 25 x 10^9/l	Interrupt lenalidomide treatment
Return to ≥25 x 10^9/l	Resume lenalidomide at a dose of 5 mg/d
**Current dose level 5mg**	
**When platelets**	**Recommended Course**
Fall to < 25 x 10^9/l	Interrupt lenalidomide treatment
Return to ≥ 25 x 10^9/l	Resume lenalidomide at a dose of 5 mg/d
For a subsequent drop below 30 x 10^9/l	Discontinue lenalidomide study drug.

- Non-hematological toxicities

CTC Toxicity Grade	Recommended course
**Non-blistering rash Grade 3**	• Interrupt lenalidomide treatment. Restart at next lower dose level after the toxicity has resolved to ≤ grade 1
**Grade 4**	• Discontinue lenalidomide study drug.
**Desquamating (blistering) rash- any Grade**	• Discontinue lenalidomide study drug.
**Sinus bradycardia/ other cardiac arrhythmia Grade 2**	• Interrupt lenalidomide treatment. Restart at next lower dose level after the toxicity has resolved to ≤ grade 1
**≥ Grade 3**	• Discontinue lenalidomide study drug.
**Neuropathy Grade 3**	• Interrupt lenalidomide treatment. Restart at next lower dose level after the toxicity has resolved to ≤ grade 1
**Grade 4**	• Discontinue lenalidomide study drug.
**Allergic reaction or hypersensitivity Grade 2**	• Interrupt lenalidomide treatment. Restart at next lower dose level after the toxicity has resolved to ≤ grade 1
**Grade 3-4**	• Discontinue lenalidomide study drug.
**Constipation (≥ Grade 3)**	• Interrupt lenalidomide treatment. Initiate appropriate treatment and restart at next lower dose level after the toxicity has resolved to ≤ grade 2
**Venous thrombosis/ embolism ≥ Grade 3**	• Interrupt dose and start anticoagulation; restart at investigator's discretion (maintain dose level).
**Hyperthyroidism or hypothyroidism**	• Interrupt lenalidomide, evaluate etiology, and initiate appropriate therapy. Restart lenalidomide at investigator's discretion (maintain dose level)
**Erythema multi- forme ≥ Grade 3**	• Discontinue lenalidomide study drug.
**Other non-hematologic toxicity assessed as Lenalidomide-related ≥ Grade 3**	• Interrupt treatment. If the toxicity resolves to ≤ grade 2 lenalidomide can be restarted at next lower dose level

9.1.6 Bisphosphonates

It is strongly recommended to start treatment with i.v. bisphosphonates at diagnosis and to continue this treatment every 4 weeks for at least 2 years. A commonly used regimen consists of zoledronate 4 mg or ibandronate 6 mg i.v. once every 4 weeks.

9.1.7 Platelet and Red Cell Transfusions

9.1.7.1 Guidelines for Platelet Transfusions

Thrombocytopenia can occur as a consequence of bone marrow infiltration by myeloma cells or may be related to study drug administration. The clinical significance of thrombocytopenia experienced by a patient should be assessed in light of its etiology (bortezomib, lenalidomide or disease or both), the state of the underlying myeloma (stable versus worsening disease), and whether the patient is bleeding or being prepared for a surgical procedure.

The use of any platelet product should be considered in the following circumstances:

• As preparation for an invasive surgical procedure, transfuse in order to maintain a platelet count > 50 x 10^9/l to prevent bleeding.

• If the patient has an active infection, high fever, rapid decrease in platelet count to ≤ 20 x 10^9/l and/or coagulopathy, transfuse to maintain a platelet count to > 20 x 10^9/l as prophylaxis for spontaneous bleeding.

• If the patient is actively bleeding or has a platelet count below 10 x 10^9/l, transfuse in order to maintain a platelet count > 10 x 10^9/l.

9.1.7.2 Guidelines for Red Cell Transfusions

The use of any red cell product should be considered in the following circumstances:

• If the patient has a hemoglobin < 7 g/dl, transfuse to maintain a hemoglobin > 8.0 g/dl in order to reduce the risk of inadequate oxygenation.

• If the patient is asymptomatic and has a hemoglobin between ≥ 7 g/dl and ≤ 8 g/dl, the investigator may consider transfusion on a per-patient basis in order to maintain a hemoglobin > 8 g/dl.

• If the patient is actively bleeding or has symptomatic cardiac or pulmonary disease or other extenuating circumstances where oxygenation is impaired, the investigator may elect to transfuse on a per-patient basis. In these instances, the trigger hemoglobin value may be > 8 g/dl.

9.2 Methods of Data Collection

In section 9.3 the required investigations are described in detail.

The required investigations are consistent with those recommended for myeloma patients in routine medical care, except for some additional investigations of the scientific programme (see below).

Some of the listed investigations are not mandatory for all patients (e.g. pregnancy test only for women of childbearing potential) or only have to be performed under specific conditions (specified below and in appendix IV).

Some investigations or findings are listed in the protocol in order to provide information about standard care in multiple myeloma even though the corresponding data are not collected in this trial. Therefore some investigations or findings won't be asked for in the CRF directly. However, in case the results of the investigations fulfil the criteria for a reportable adverse event (see chapter 11), this has to be documented. Furthermore, consequences of pathologic results such as resulting dose reductions are asked for.

Investigations or findings that are not asked for in the CRF directly are shown in italics (*cursive*).

In general the laboratory investigations (efficacy and safety parameter) will be performed locally at the site.

Samples to be shipped for central investigations are (also see appendix IV. Forms for shipment incl. instructions see additional study documents in the ISF):

- at study entry (and at relapse)

- bone marrow aspirate (80 ml, heparinized) for iFISH diagnostics and gene expression profiling
- peripheral blood (20 ml),
- serum (2 x 7.5 ml),

- during treatment

- serum (2 x 7.5 ml) (at each evaluation of response)
- peripheral blood (20 ml) after induction therapy, previous to consolidation treatment and previous to maintenance treament
- Bone marrow sample (15 ml, heparinized) for MRD diagnostic (FACS, ASO-PCR if applicable) at specific timepoints (see section 9.3.2)

Regarding the central diagnostic only the results of the freelite test in serum have to be documented in the CRF. The results of iFISH, MRD diagnostic and the scientific programme will be added to the database centrally, if applicable.

9.3 Required Investigations at Entry, during Treatment and during Follow Up

The frequency and timing of required investigations is summarized in the schedule in appendix IV. Details are described in chapter 9.3.1 and 9.3.2. Investigations or findings that are not asked for in the CRF directly are shown in italics (*cursive*).

For documentation of adverse events and serious adverse events please see chapter 11.

9.3.1 Clinical Investigations at Entry

Aim of the clinical evaluation prior to the study treatment is to determine the indication for systemic therapy (symptomatic MM), the eligibility for the treatment, to know in which stage of disease according to Salmon & Durie the patients are classified and to determine the presence of adverse prognostic factors. Additionally, the investigations are essential to establish a baseline for response evaluations after treatment.

At start of the study, prior to the treatment, the following investigations have to be performed. These investigations are consistent with the routine medical care for myeloma patients at diagnosis and prior to treatment. If routine data of patients are available which, in the investigator's opinion, are still representative, these data should be used and need not to be repeated, if they have been performed up to 3 weeks prior to enrolment (exceptions and/or special instructions see details below).

- Standard medical history, with special attention for:
    - WHO performance status (see appendix IIIA)
    - polyneuropathy
    - prior and present other diseases
    - antecedent hematological or oncological diseases
    - previous chemotherapy or radiotherapy
    - female patients: childbearing potential
- Standard physical examination including body weight and height, with special attention for:
    - polyneuropathy or other neurologic symptoms

- infections

- Hematology
  - hemoglobin
  - leukocyte count, differential count
  - platelets

- Blood chemistry / urine chemistry
  - total proteins
  - albumin
  - creatinine
  - ASAT (sGOT), ALAT (sGPT), γ-GT
  - urea
  - total bilirubin
  - alkaline phosphatase
  - LDH
  - CRP
  - calcium, natrium (sodium), kalium (potassium)
  - uric acid
  - total proteins in 24h urine (please note: for this analysis no dipsticks should be used)

- Immunochemistry

Please note that the investigations for quantification of Serum and urine M-Protein have to be performed at trial site and no results of previous investigations done outside the site should be used (to ensure comparability of later investigations for response evaluation)

  - Serum protein electrophoresis (SPEP)
    (for quantification of the monoclonal protein in Serum (Serum M-Protein)).
  - Quantitative light chain excretion in 24h urine
    (monoclonal protein in urine (Urine M-Protein)).Please note that quantification of light chain excretion in urine should not be performed by sFLC assay.
  - Immunofixation serum
  - Immunofixation urine
  - IgG, IgA, IgM and, in case of IgD myeloma, IgD concentration in serum

- Bone marrow
  - Bone marrow biopsy (not mandatory, according to local policy)
  - Bone marrow aspirate at entry for:
    - determination of plasma cell infiltration (local investigation at site)
    - FISH-Analysis (central investigation, see 9.3.2)
    - Molecular analysis (DNA microarray analysis, central investigation, see 9.3.2)

- Specific investigations
  - Serum beta-2 microglobulin
  - Creatinine clearance
  - *x-ray thorax* (not required in case of whole body CT),
  - *ECG*
  - *Cardiac ejection by cardiac echo; it is advised to determine the left ventricular ejection fraction (LVEF) in all patients at entry (i.e. prior to start of treatment).*
  - hCG pregnancy test (for females of childbearing potential)

- Imaging for determination of myeloma-related bone disease, bone marrow and soft tissue involvement
    - for initial work-up a whole body low dose CT-scan and a whole body MRI, if available, is recommended. Application of skeletal survey (x-ray) is allowed if CT is not available. In case of results of unclear significance a central second assessment can be performed. For central diagnostic CDs with CT-findings in DICOM-format can be sent to Prof. Dr. M. Horger (address see page 6) and CDs with MR-images to Prof. Dr. S. Delorme (address see page 6). Please enclose an accompanying form for shipment (see ISF).
    - If whole body MRI is not available in a study center, whole body low dose CT in combination with spinal MRI should be performed

  If imaging results as described above are available that are still representative in the investigator's opinion, it is not needed to repeat these procedures at entry (performed up to 6 weeks prior to randomisation).

9.3.2 Clinical Investigations during Treatment

9.3.2.1 Clinical Evaluations for Response Assessment

Aim of the clinical evaluation during treatment and follow up is to determine response, toxicities and eligibility for further treatment.

Response to treatment will be evaluated according to the IMWG criteria (see appendix II).

Timepoints of Clinical Evaluations

The following table shows the recommended timepoints of response evaluation. A non-essential deviation of these timepoints (e.g. for logistic reasons) is accepted, but it is important that the reponse to a treatment period will be assessed previous to the start of the subsequent period.

Treatment period	Timepoint of response evaluation
after Induction treatment - arms A1 and B1	d21 - d28 after start of 3rd cycle PAd
after Induction treatment - arms A2 and B2	d21 - d28 after start of 3rd cycle VCD
After first chemotherapy cycle of intensification regime	d23 – d33 after start of this cycle
After subsequent chemotherapy cycles of intensification regime	According to local policy: d60 - d90 after start of intensification cycle recommended (previous to next cycle)
After R consolidation	d23 - d33 after start of 2nd cycle R
During maintenance	every 3 months
During follow up within 2 years after start of maintenance (until 1st PD)	every 3 months
Follow up, after the first 2 years after start of maintenance (until 1st PD)	every 6 months

During treatment the following investigations have to be performed. These investigations are consistent with the routine medical care for myeloma patients. Please note that the investigations for **response assessment** have to be performed at main trial site.

- Standard medical history, with special attention for:
  - WHO performance status (see appendix IIIA)
  - infections
  - polyneuropathy
  - *hospitalisations* (please check if an SAE report is necessary)
- Standard physical examination including body weight and height, with special attention for:
  - polyneuropathy or other neurologic symptoms
  - infections
- Hematology
  - Hemoglobin
  - Leukocyte count, differential count
  - Platelets
- Blood chemistry / urine chemistry
  - Total proteins
  - Albumin
  - Creatinine
  - *ASAT (sGOT),* ALAT (sGPT), *γ-GT*
  - *Urea*
  - *Total bilirubin*
  - *Alkaline phosphatase*
  - LDH
  - CRP
  - Calcium, *natrium (sodium), kalium (potassium)*
  - *Uric acid*
  - Total proteins in 24h urine (please note: for this analysis no dipsticks should be used)
- Immunochemistry
  - Serum protein electrophoresis (SPEP) for quantification of the monoclonal protein in Serum (serum M-protein)
  - Quantitative light chain excretion in 24h urine (monoclonal protein in urine (urine M-protein)). Please note that quantification of light chain excretion in urine should not be performed by sFLC assay.
  - Immunofixation serum (for confirmation of CR, only necessary if no M-peak in SPEP)
  - Immunofixation urine (for confirmation of CR, if at the actual measurement light chains are within normal ranges)
  - IgG, IgA, IgM and, in case of IgD myeloma, IgD concentration in serum
- Bone marrow
  - Bone marrow aspirate (plasma cell infiltration), if a CR can be considered

    Please note that according to the IMWG and the EBMT response criteria a bone marrow aspirate is necessary to determine if the patient has reached a CR. This is a

standard investigation in response assessment for patients with multiple myeloma that has to be done if no more monoclonal protein can be found in serum and urine (immunofixation serum and urine negative). In case the bone marrow puncture is not be done at a visit (as the investigator wants to wait for the results of SPEP and immunofixation first), there has to be an additional visit as soon as possible for the bone marrow puncture to complete the response evaluation.

- Specific investigations
  - *Creatinine clearance* (on clinical indication)
  - *x-ray thorax* (prior to intensification)
  - *ECG* (after induction; in case of normal findings prior to treatment and after induction, thereafter only on clinical indication)
  - *Cardiac ejection* (LVEF) by cardiac echo (after induction; in case of normal findings prior to treatment and after induction therapy , thereafter only on clinical indication).
  - *hCG pregnancy test* (for females of childbearing potential - before and during lenalidomide treatment (every 4 weeks), 4 weeks after lenalidomide treatment)
- Imaging for determination of myeloma-related bone disease, bone marrow and soft tissue involvement
  - In general follow-up imaging should be performed on the discretion of the investigator based on clinical aspects and previous findings.
  - follow-up imaging is recommended as follows (for the individual patient please always use the same imaging technique initially and in follow up to ensure comparability):
    - whole body low dose CT-scan or skeletal survey prior to consolidation therapy, preferably in combination with a whole body MRI.
    - during further treatment or follow up imaging is mandatory only if symptoms occur or serological progression is present
  - In case of results of unclear significance a central second assessment can be performed. For central diagnostic CDs with CT-findings in DICOM-format can be sent to Prof. Dr. M. Horger (address see page 6) and CDs with MR-images to Prof. Dr. S. Delorme (address see page 6). Please enclose an accompanying form for shipment (see ISF).

9.3.2.2 Additional Investigations during Treatment (Safety Parameter)

Before start of each treatment cycle and during the cycles, routine investigations like blood cell count and renal function will be performed according to local policy.

Safety Parameter during Lenalidomide Treatment

- during the first 2 months: *hematology (blood count) once weekly*
- subsequently: *hematology (blood count) once monthly*

9.3.3 Central Diagnostic and Scientific Programme

For an overview of samples investigated centrally (including peripheral blood, serum, bone marrow aspirates, bone marrow biopsies), see appendix IV or section 9.2. A more detailed description about samples for molecular profiling and MRD-diagnostics can be found below.

Molecular Profiling of Myeloma (Cytogenetic Analysis (interphase FISH), Gene Expression Profiling, Flow Cytometry, Free light chain and Heavy chain Test)

FISH analysis, gene expression profiling and the assessment of the malignant phenotype (by flow cytometry) will be performed in all patients at diagnosis (central diagnostic), see section 3

for details. For these iFISH analyses, a bone marrow sample (80 ml of heparinized bone marrow, i.e., 4x18 ml BM plus 2ml of Heparin in each syringe, aspirated with three bone marrow punctures within one anesthetised region) has to be sent in previous to start of chemotherapy.

All iFISH results will be stored in a central database at the GMMG-study office. As iFISH diagnostic is part of the routine diagnostic in myeloma, participating German centres are kindly requested to supply a referral form ("Überweisungsschein") when sending the BM-sample, and will receive a written report about the findings within 4 weeks. If no referral form is supplied, the iFISH results necessary for risk stratification (i.e., t(4;14) and del17p13) are available from the GMMG-study office (contact see page 2).

The free light chain ratio in serum will be assessed centrally at inclusion and each response evaluation. An aliquot of the serum sample will be stored for later analyses using the hevylite test (to be performed on a subset of patients within the scope of minimal residual disease analyses). For these analyses, a serum sample (2 serum monovettes, i.e 2x7.5 ml) has to be sent.

Assessment of Minimal Residual Disease
For all patients in CR according to the IMWG or EBMT criteria, presence of stringent complete remission and molecular complete remission will be assessed in a stepwise approach (see also section 3.1). For MRD-analysis, a bone marrow sample (20 ml, heparinized) will be sent in. Thereby, the following **algorithm** will be used (see also figure 2):

a) **For Patients not known to be in CR**.

a1) If a bone marrow aspiration is performed to assess CR at a time where the actual κ/λ-chain ratio or immunofixation in urine / serum still have to be determined: An additional sample of 15 ml BM will be drawn and sent for central MRD-assessment (address see page 5 of the study protocol). The sample will not be analyzed for MRD (mCR and sCR) if in the meantime any data are available excluding an mCR (e.g., abnormal κ/λ--ratio in free light chain assay or positive immunofixation in either serum or urine).

a2) If a bone marrow aspiration is performed to assess CR, and at that time any data are available excluding an mCR (e.g., abnormal κ/λ--ratio in free light chain assay or positive immunofixation in either serum or urine), no sample for MRD diagnostics will be drawn.

a3) In case of doubt (e.g., difficulties to interpret the free κ/λ--ratio) the sample of 15 ml BM will be assessed for MRD.

b) **For Patients known to be in CR.**

b1) A MRD assessment will be performed for all patients in CR with normal κ/λ--ratio in free light chain assay at the next scheduled visit before next treatment block.

b2) If after BMA for MRD any data become available excluding sCR or mCR (e.g., abnormal κ/λ-ratio at this subsequent visit), no MRD diagnostic is performed at this visit.

b3) For patients in CR who underwent BMA for MRD diagnostic, MRD assessment will be repeated once after 6 months.

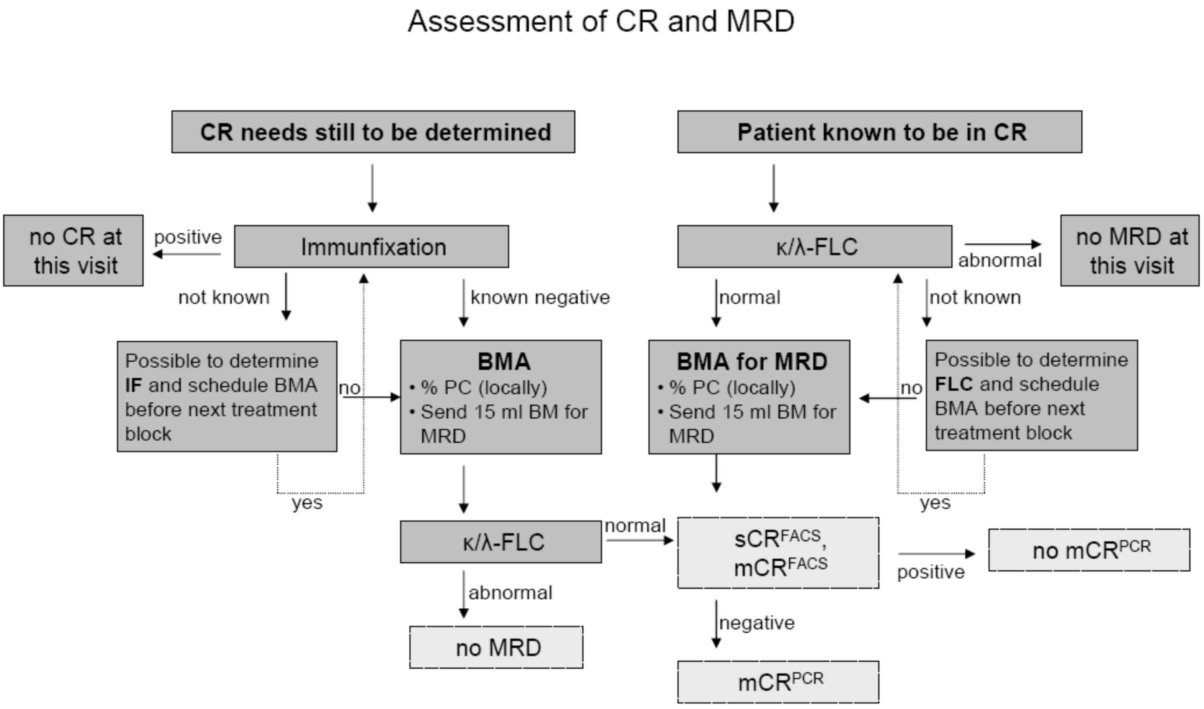

Figure 2. Algorithm for MRD-assessment (CR= complete response; MRD= minimal residual disease; FLC= free light chain ratio, IF=immunofixation; BM(A)= bone marrow (aspiration); PC= plasma cells; sCR and mCR $^{FACS/PCR}$ see text)

9.4 Efficacy Parameters

- monoclonal protein in serum
- monoclonal protein in urine
- FLC in serum
- plasma cell infiltration
- bone disease
- soft tissue plasmocytoma
- survival

9.5 Safety Parameters

- (serious) adverse events (see section 11)
- laboratory findings (hematology, creatinine, blood chemistry incl. ASAT, ALAT, γ-GT, urea, bilirubin, etc., hCG for women of childbearing potential)
- physical examination
- medical history
- ECG and cardiac echo

10 Plan for Treatment or Care after the Trial

Treatment after regular Completion of the Study Treatment

In the study arms A1 and A2 lenalidomide maintenance treatment will be performed for 2 years, thereafter the patient goes "off study" and the lenalidomide therapy should be discontinued.

Currently long term maintenance treatment with lenalidomide is evaluated in clinical trials. By October 2012 (i.e., date first patient will complete regular study treatment) long term maintenance data with lenalidomide will be available from the IFM trial (IFM 2005-02[11]) and other prospective trials. According to these data the PI and the GMMG advisory board will decide if the recommendation to stop maintenance treatment after two years will be kept.

Treatment after Stop of Study Treatment due to Progressive Disease/Relapse

In patients with relapsed myeloma (reason for going off protocol), the treatment options depend on the compounds used in prior therapy, the duration of response, performance status, type of relapse, and previous toxicity. The decision about relapse treatment for a patient of the MM5 trial should be made by the investigator considering these issues. During follow up of the MM5 trial basic data concerning the relapse treatment will be collected. It is recommended that relapse treatment will be performed within a clinical trial.

11 Adverse Events

11.1 Definitions

11.1.1 Adverse Event

According to GCP, an adverse event (AE) is defined as follows: Any untoward medical occurence in a subject administered a pharmaceutical product and which does not necessarily have a causal relationship with this treatment. An AE can therefore be any unfavourable and unintended sign (including an abnormal laboratory finding), symptom, or disease temporally associated with the use of a medicinal (investigational) product, whether or not related to the medicinal (investigational) product.

An AE may be:

- New symptoms/ medical conditions
- New diagnosis
- Changes of laboratory parameters
- Intercurrent diseases and accidents
- Worsening of medical conditions/ diseases existing before clinical trial start
- Recurrence of disease
- Increase of frequency or intensity of episodical diseases.

A pre-existing disease or symptom will not be considered an adverse event unless there will be an untoward change in its intensity, frequency or quality. This change will be documented by an investigator.

Surgical procedures themselves are not AEs; they are therapeutic measures for conditions that require surgery. The condition for which the surgery is required may be an AE. Planned surgical measures permitted by the clinical trial protocol and the condition(s) leading to these measures are not AEs, if the condition leading to the measure was present prior to inclusion into the trial.

AEs are classified as "non-serious" or "serious".

11.1.2 Serious Adverse Event

A serious adverse event (SAE) is one that at any dose:

- Results in death
- Is life-threatening (the term life-threatening refers to an event in which the subject was at risk of death at the time of event and not to an event which hypothetically might have caused death if it was more severe)
- Requires hospitalisation or prolongation of existing hospitalisation
- Results in persistent or significant disability/ incapacity or
- Is a congenital anomaly/ birth defect
- is a malignant neoplasm
- Is otherwise medically relevant

Medical and scientific judgement should be exercised in deciding whether expedited reporting is appropriate in other situations - such as important medical events that may not be immediately life threatening or result in death or hospitalisation but may jeopardize the patient or may require intervention to prevent one of the other outcomes listed above. *These should also usually be considered serious*. (examples of such events are intensive treatment in an emergency room or at home for allegic bronchospasm; blood dyscrasias or convulsions that do not result in hospitalisation; or development of drug dependency or drug abuse).

Second primary malignancies will be monitored as events of interest and must be reported as serious adverse events regardless of the treatment arm the subject is in. This includes any second primary malignancy, regardless of causal relationship to study drugs, occurring at any time for the duration of the study, from the time of randomisation up to the end of study participation (including the “2 year maintenance period”, regardless if study drug was taken; also see 11.2). Events of second primary malignancy are to be reported using the SAE report form and must be considered an “Important Medical Event” even if no other serious criteria apply; these events must also be documented in the appropriate page(s) of the CRF and subject’s source documents. Documentation on the diagnosis of the second primary malignancy must be provided at the time of reporting as a serious adverse event (e.g., any confirmatory histology or cytology results, X-rays, CT scans, etc.). During the follow up period (after end of study) second primary malignancies have to be reported on the appropriate pages of the eCRF.

In addition, any second primary malignancy (SPM) occurring in a patient during long-term follow up periods shall also be considered a serious adverse event and be reported to Celgene GmbH, Arzneimittelsicherheit (drugsafety-germany@celgene.com or Fax +49-(0)89-451 519-023). For the purposes of this provision, “long-term follow up” shall mean a period of at least three years after last dose of study product.

11.1.3 Adverse Drug Reaction

All noxious and unintended responses to a medicinal product related to any dose should be considered adverse drug reactions (ADR).

11.1.4 Expectedness

An ‘unexpected’ adverse event is one the nature or severity of which is not consistent with the applicable product information, e.g., Investigator’s Brochure (IB) or Summary of Product Characteristics (SmPC). Furthermore, reports which add significant information on specificity or severity of a known adverse reaction constitute ‘unexpected’ events.

11.1.5 Suspected Unexpected Serious Adverse Reaction (SUSAR)

SAEs that are both suspected, i.e., possibly related to the study drug/Investigational medicinal product (IMP) and 'unexpected', i.e., the nature and/ or severity of which is not consistent with the applicable product information are to be classified as Suspected Unexpected Serious Adverse Reactions (SUSARs).

In case, either the investigator who primary reported the SAE or the second assessor classify the SAE as 'suspected' (i.e., either as definitely or probable or possible related to IMP or "not assessable") and the SAE is unexpected it will be categorized as a SUSAR.

All SUSARs are subject to an expedited reporting to the responsible ethics committee(s), the competent authority and to all participating investigators. For details see Safety Manual.

11.1.6 Grading of AEs

The grading of AEs in this trial will be carried out on the basis of the 5-grade scale defined in the CTCAE v4.0:

Grade 1: mild AE

Grade 2: moderate AE

Grade 3: severe AE

Grade 4: life-threatening AE or AE causing disablement

Grade 5: death related to AE

The grading of all AEs listed in the CTCAE v4.0 will be based on the information contained therein. The grading of all other AEs, i.e., those which are not listed in the CTCAE v4.0 will be performed by a responsible investigator, based on definitions given above.

11.1.7 Relationship and Outcome of AEs

The investigator will evaluate each AE that occurred after administration of investigational medicinal product regarding the coherency with the administration of the investigational medicinal product possibly:

related: There is a reasonable possibility that the event may have been caused by IMP. A certain event has a **strong temporal relationship** and an alternative cause is unlikely.

probable: An AE that has a reasonable possibility that the event is likely to have been caused by IMP. The AE has a **timely relationship** and **follows a known pattern of response**, but a potential alternative cause may be present.

possible: An AE that has a reasonable possibility that the event may have been caused by IMP. The AE has a **timely relationship** to the IMP; **however, the pattern of response is untypical**, and an alternative cause seems more likely, or there is significant uncertainty about the cause of the event.

unlikely: Only a remote connection exists between the IMP and the reported adverse event. Other conditions including concurrent illness, progression or expression of the disease state or reaction of the concomitant medication appear to explain the reported adverse event.

not related: An AE that does not follow a reasonable temporal sequence related to IMP and is likely to have been produced by the subject's clinical state,

other modes of therapy or other known etiology.

not assessable: There is insufficient or incomplete evidence to make a clinical judgement of the causal relationship.

All subjects who have reportable AEs, whether considered associated with the use of the trial medication or not, must be monitored to determine the outcome. The clinical course of the AE will be followed up until resolution or normalization of changed laboratory parameters or until it has changed to a stable condition.This also holds for ongoing AEs/SAEs of withdrawn subjects.

The outcome of an AE at the time of the last observation will be classified as:

'Recovered/ resolved':
all signs and symptoms of an AE disappeared without any sequels at the time of the last interrogation.

'Recovering/ resolving':
the intensity of signs and symptoms has been diminishing and / or their clinical pattern has been changing up to the time of the last interrogation in a way typical for its resolution.

'Not recovered/ not resolved':
signs and symptoms of an AE are mostly unchanged or worsened at the time of the last interrogation.

'Recovered/ resolved with sequel':
actual signs and symptoms of an AE disappeared but there are sequels related to the AE.

'Fatal': resulting in death. If there are more than one adverse event only the adverse event leading to death (possibly related) will be characterized as 'fatal'.

'Unknown': the outcome is unknown or implausible and the information cannot be supplemented or verified.

The action taken with IMP will be assigned to one of the following categories:

'Dose not changed': no change in the dose of IMP.
'Dose reduced': reduction in the dose of IMP.
'Drug withdrawn': discontinuation of IMP.
'Unknown': the information is unknown or implausible and it cannot be supplemented or verified.
'Not applicable': the question is implausible (e.g., the subject is dead).

The term 'countermeasures' refers to the specific actions taken to treat or alleviate adverse events or to avoid their sequels. Following categories will be used to categorize the countermeasures to adverse events:

None: no action taken
Drug treatment: newly-prescribed medication or change in dose of a medication
Others: other countermeasures, e.g., an operative procedure

11.2 Period of Observation and Documentation

AEs reported by the subject or detected by the investigator will be collected during the trial and must be documented on the appropriate pages of the CRF if the criteria described below are fulfilled. In general AEs should be documented in the subject's medical records.

Untoward medical occurrences that are neither critical to safety evaluations in the trial nor relevant for the final data analyses of the trial should not be reportable as adverse events.[85-87] Thus in this trial the requirements for AE reporting (regarding extent and type of AE data to be collected) are differentiated according to severity of the event and/or the treatment phase, as described below.

The study drugs bortezomib and lenalidomide are approved drugs for use in patients with multiple myeloma. Broad safety data already are available.

Secondary objective of this trial is the evaluation of toxicity CTC grade $\geq$ 3 during the treatment phases including bortezomib or lenalidomide, respectively. Thus, in general only AEs CTCAE grade 3, 4 and 5 in these treatment phases (and a defined subsequent period) have to be recorded for the trial. Since some specific occurrences will be evaluated in respect of their frequency in consolidation and maintenance treatment, they will additionally be documented. So the following AEs of CTCAE grade 2 have to be recorded and will be asked for in the CRF for the induction, consolidation and maintenance treatment:

- polyneuropathy
- thromboembolic events (not including superficial thrombophlebitis or anal thrombosis)
- infections
- cardiac events

As broad safety data for bortezomib and lenalidomide are available for the benefit/risk assessment and non-serious adverse events classified as ‘mild' and ‘moderate' (CTC AE grade 1 and 2) do not influence the benefit/risk profile in the study population they will not be documented as AE.

Subsequent to the induction therapy, the standard intensification treatment for myeloma patients will be performed according to local policy as it is also performed outside of this trial as routine medical care in the same manner. There is no difference in the procedures in the four trial arms and the study drugs are not administered in this trial phase. The toxicity of intensification in myeloma patients already has been analysed in several trials and registries including more than 30.000 patients, thus the toxicity is known. Trial-specific adverse events are not expected. So it is no objective of this trial to evaluate toxicity of standard intensification treatment and thus non-serious AEs in this phase do not have to be recorded for the trial.

Serious adverse events have to be reported in all treatment phases regardless of the CTC AE grade. Even though there are no trial-specific AEs expected during the “intensification phase” serious adverse events remain reportable during the whole study period for safety reasons. All SAEs and their relevance for the benefit/risk assessment for the study will be evaluated continuously during the study and for the final report.

After start of consolidation and during maintenance period all untoward medical occurrences CTC grade $\geq$ 3 (and for specific AEs also CTC grade 2, see below) should be reported for a period of two years after start of maintenance as AEs even if the lenalidomide treatment has been stopped (prematurely or according to protocol after achievement of a CR in the arms B1 and B2). This is necessary for the assessment of the total number of AEs (CTC grade $\geq$ 3) during lenalidomide maintenance and the comparison of the four arms.

In Summary and Detail AEs in this Trial have to be reported as follows (Serious adverse events have to be reported during the whole study period, regardless the CTC AE grade) :

Period of induction treatment (“Induction”)

After start of study treatment, during induction treatment and subsequent 30 days **all AEs CTCAE grade 3, 4 and 5** have to be reported (AEs occurring up to 30 days after end of induction treatment or until start of the following cycle of intensification chemotherapy, whichever comes first). Additionally, the specific AEs polyneuropathy, thromboembolic events, cardiac events and infections already have to be reported from CTCAE grade 2.

Period of Intensification therapy (“Intensification”)

During the phase of intensification therapy according to local standard protocols **only serious adverse events** have to be reported (except for a period of 30 days after the last day of induction treatment, see above).

Period of R consolidation and maintenance treatment (“Post-Intensification”)

After start of R consolidation treatment until 30 days after end of the “2 year-maintenance-period” (i.e., the period of 2 years after start of maintenance treatment, regardless if lenalidomide was taken) **all AEs CTCAE grade 3, 4 and 5** have to be reported. Additionally, the specific AEs polyneuropathy, thromboembolic events, cardiac events and infections already have to be reported from CTCAE grade 2.

In general SAEs occurring up to 30 days after last study visit or until start of a subsequent chemotherapy (e.g. relapse treatment), whichever comes first, shall be reported.

11.3 Reporting of Serious Adverse Events by Investigator

All SAEs must be reported by the investigator to the responsible Safety Officer at the KKS Heidelberg within 24 hours after the SAE becomes known using the "Serious Adverse Event" form. The initial report must be as complete as possible including details of the current illness and (serious) adverse event and an assessment of the causal relationship between the event and the trial medication.

The following events need not to be reported as SAE:

- aplasia during standard intensified treatment regime (leukopenia/neutropenia/thrombocytopenia CTC °4)

Any complication from the aplasia, that itself fulfils the SAE criteria - including (prolongation of) hospitalisation or unexpected duration - remains reportable as a Serious Adverse Event.

Hospitalisations as a result of the following causes need not to be reported as SAE. Any complications from the events, that itself fulfil the SAE criteria - including (prolongation of) hospitalisation or unexpected duration - remain reportable as a Serious Adverse Events:

- drug application (e.g., chemotherapy, study medication)
- administration of blood or platelet transfusion
- test procedure required in the protocol
- technical, practical, or social reasons, in absence of an adverse event
- surgical intervention or other measures and the condition(s) leading to these measures are not AEs, if the condition leading to the measure was present prior to inclusion into the trial.
- stay at rehabilitation clinic
- progression of multiple myeloma

11.4 Expedited Reporting

SUSARs are to be reported to the ethics committee(s), competent authority and to all participating investigators within defined timelines, i.e., they are subject to an expedited reporting.

Investigators participating in this trial will report all SAEs to a responsible safety officer at the KKS Heidelberg as soon as possible but not later then 24 hours after their notification. The reporting will be performed by faxing of a completed 'SAE Form' to the KKS Heidelberg, fax-number: +49-(0)6221-56-33725

All SAE will be subject to a second assessment by the principal investigator or a designated person.

The second assessor will fill out a 'Second Assessment Form' for each SAE and send it back per fax to the responsible person at the KKS Heidelberg within 48 hours.

The 'Second Assessment Form' will contain the following information:

I) assessment of relationship between SAE and IMP
II) assessment of expectedness of SAE (derived from IB or SmPC)
III) statement if the benefit/ risk assessment for the trial did change as a result of SAE.

The expedited reporting will be carried out by a responsible safety officer at KKS Heidelberg. Only SUSARs occurring after administration of IMP will undergo expedited reporting.

11.5 Emergency Treatment

During and following a subject's participation in the trial, the investigator should ensure that adequate medical care is provided to a subject for any AE including clinically significant laboratory values. The investigator should inform a subject when medical care is needed for intercurrent illness(es) of which the investigator becomes aware.

12 Statistical Procedures

12.1 Sample Size Calculation

The MM5 trial is designed to address two independent primary objectives. In order to guarantee a family-wise error rate of 5%, each primary objective will be tested at the two-sided alpha level of 2.5%.

The first primary objective is to compare the VGPR$^+$ (at least very good partial response) rate between the two induction regimens in a parallel two-arm design with a non-inferiority hypothesis. VGPR$^+$ is defined as achieving very good partial response or better after induction therapy. According to the ongoing GMMG-HD4 trial, a VGPR$^+$ rate of 42% for the standard arm (PAd: A1+B1) is assumed. Assuming a VGPR$^+$ of 46% for the VCD arm (A2+B2)[88], a total of 478 patients are required to demonstrate non-inferiority at a non-inferiority margin of 10% difference with 80% power at an one-sided significance level of $\alpha=0.0125$. After accounting for 5% patients not being eligible for per-protocol population, 504 patients need to be enrolled. This sample size calculation is based on the method of Farrington and Manning [89].

The second primary objective is to determine the best of the four treatment arms with regard to progression-free survival (PFS). Assuming 3 years of recruitment, 3 years minimal follow-up time, a total of 10% drop-outs and 5% high risk patients leaving the study prematurely after induction therapy, inclusion of 504 patients allows for rejecting the global null hypothesis of no difference between the four arms at the two-sided significance level of 0.025 with a power of

80%, if the arms achieve PFS rates of 75%, 65%, 65% and 55% after 3 years. This corresponds to hazard ratios relative to the best arm of 1.5, 1.5 and 2.1. The PFS rates of the intermediate arms are conservatively chosen representing the least favourable distribution w.r.t. to power. No assumption about the order of treatment arms is made. Further treatment comparisons will be realized within a closed testing procedure. The sample size calculation is based on the method of Barthel *et al.* [90] for multi-arm survival trials.

An interim analysis with respect to response rates after induction treatment will be conducted to rule out lack of effect. Both induction treatments will be assessed separately for efficacy according to an optimal two-stage design after 75 patients in each group have been enrolled. If more than 23 patients (30.7%) have responded, enrolment in this group will be continued. An optimal two-stage phase II design [91] with $\alpha=\beta=0.1$, an unacceptable $VGPR^+$ rate of 30% and an desirable $VGPR^+$ rate of 40% was used to determine the interim analysis of response rates within each induction therapy arm. This interim analysis will be performed to establish efficacy of both induction regimens.

12.2 Analysis Variables

Primary Analysis Variables

There are separate analysis variables for both primary analyses. The first primary analysis compares response to VCD and PAd induction treatment in a parallel two-arm design (PAd:A1+B1 vs. VCD:A2+B2) for non-inferiority. The analysis will be based on the $VGPR^+$ rate which is the proportion of patients with at least very good partial response to treatment after induction therapy in each arm.

The second primary analysis is to compare all four treatment strategies (A1, A2, B1, B2) in a parallel four-arm design in progression-free survival (PFS). Progression-free survival (PFS) is defined as time from randomisation to progression, relapse or death, whichever occurs first. Patients without progress or relapse and still being alive at the time of the analysis will be censored at the date of the last response assessment. Patients without any response assessment after randomisation will be censored at the date of randomisation. High risk patients leaving the study and receiving an allogeneic transplantation will be censored at the date of transplantation.

For both primary analysis variables, response to treatment will be evaluated according to IMWG criteria (see appendix II) at the scheduled time points as specified in section 9.3.

Secondary Analysis Variables

Secondary analysis variables include

- toxicity during induction therapy, lenalidomide consolidation and maintenance therapy, respectively, measured by CTC-AE (v4.0)
- overall survival defined as time from randomisation to time of death from any cause. Patients still being alive at the time of the analysis will be censored at the date last known to be alive. High risk patients leaving the study and receiving an allogeneic transplantation will be censored at the date of transplantation.
- complete response rates after consolidation therapy
- best response during the study (including mCR)

12.3 Definition of Trial Population to be Analyzed

Three analysis populations will be defined. The first primary objective will be analysed for the per-protocol (PP) population and intent-to-treat (ITT) population. The second primary objective will analysed using the intent-to-treat population.

Secondary efficacy analysis will be based on ITT. All safety analysis will be performed for the safety population (SAFE).

The ITT population is defined according to the intent-to-treat principle and consists of all patients randomised. Patients will be analysed as randomised. The per-protocol population is defined as those patients in the ITT population who completed the 3 cycles induction therapy including the subsequent response assessment without major protocol violations. PP patients will be analysed as treated. The safety population comprises all patients who received at least one dose of study medication. Patients will be analysed as treated.

Not included in ITT and PP are patients who withdraw informed consent before start of treatment or about whom it becomes known, that major in/exclusion criteria were violated which would have excluded them from study treatment when known at start of treatment.

12.4 Statistical Methods

A detailed statistical analysis plan (SAP) will be finalized before the first interim analysis and the closure of the database, respectively which has to be authorized by the biometrician, the sponsor, and the LKP.

Analyses of both primary endpoints are confirmatory. All remaining analyses are exploratory and will be carried out at a two-sided significance level of 0.05 unless noted otherwise.

Primary Endpoints

The **first primary endpoint** is the difference in response rates (RR) after induction therapy between induction regimens (PAd:A1+B1 vs. VCD:A2+B2). The first primary analysis needs to be confirmed for both ITT and PP population as this is a non-inferiority objective. The one-sided null hypothesis tested is that the VCD arm achieves a lower response rate than the PAd arm with a difference in RR of at least 10%.The alternative is that this difference in RR does not exceed 10%:

H_0: $RR_{A2+B2} \leq RR_{A1+B1} - 10\%$ vs. H_A: $RR_{A2+B2} > RR_{A1+B1} - 10\%$

The null hypothesis H_0 will be rejected, thus non-inferiority established, if the lower limit of the two-sided 97.5% confidence interval for this difference $RR_{A2+B2} - RR_{A1+B1}$ is above the margin of -10%. The confidence interval will be calculated using Newcombe's score interval method [92]. If the lower limit of the confidence interval is above zero, a test on superiority with the null hypothesis of no difference will be performed. No adjustment for multiplicity is required since this corresponds to a simple closed testing procedure [93].

The **second primary endpoint** is progression-free survival in all four treatment arms. The second primary analysis will be based on the ITT population as this is a superiority objective. Treatment arms will be compared in a closed testing procedure as introduced by Marcus, Peritz and Gabriel [94]. This hierarchical step-down approach controls the family-wise error rate in a multi-comparison setting if all null hypotheses are tested in a pre-defined hierarchical order at a the same significance level starting with the global null and alternative hypotheses

$H_0^{1,2,3,4}: \lambda_{A1}(t) = \lambda_{A2}(t) = \lambda_{B1}(t) = \lambda_{B2}(t)$ vs.

$H_A^{1,2,3,4}: \lambda_i(t) = \theta\lambda_j(t)$, for at least one pair (*i,j*) of arms, $i, j \in \{A1, A2, B1, B2\}$

Here $\lambda_i(t)$ represents the failure rate in arm *i* at time *t* and θ is the constant of proportionality of the rates in two arms.
A hypothesis of the closed family can only be rejected if the corresponding test is significant at a given significance level and any other hypothesis in the family implying that hypothesis has also been rejected at the respective significance level. The closure principle will be followed by calculating for every hypothesis the adjusted p-value as the maximum of the p-values of all hypotheses implying that hypothesis. The four arm all-pair testing scheme comprises the following family of null hypotheses:

1. Four arm homogeneity (global) null hypothesis:
   $H_0^{1,2,3,4} : \lambda_{A1}(t) = \lambda_{A2}(t) = \lambda_{B1}(t) = \lambda_{B2}(t)$
2. Three arm homogeneity (intersection) null hypotheses:
   $H_0^{1,2,3} : \lambda_{A1}(t) = \lambda_{A2}(t) = \lambda_{B1}(t)$, $H_0^{1,2,4} : \lambda_{A1}(t) = \lambda_{A2}(t) = \lambda_{B2}(t)$
   $H_0^{2,3,4} : \lambda_{A2}(t) = \lambda_{B1}(t) = \lambda_{B2}(t)$, $H_0^{1,3,4} : \lambda_{A1}(t) = \lambda_{B1}(t) = \lambda_{B2}(t)$
   Subset intersection (partition) null hypotheses:
   $H_0^{(1,2)\cap(3,4)} : \lambda_{A1}(t) = \lambda_{A2}(t)\, and\, \lambda_{B1}(t) = \lambda_{B2}(t)$
   $H_0^{(1,3)\cap(2,4)} : \lambda_{A1}(t) = \lambda_{B1}(t)\, and\, \lambda_{A2}(t) = \lambda_{B2}(t)$
   $H_0^{(1,4)\cap(2,3)} : \lambda_{A1}(t) = \lambda_{B2}(t)\, and\, \lambda_{A2}(t) = \lambda_{B1}(t)$
3. Original pairwise homogeneity (elementary) null hypotheses:
   $H_0^{1,2} : \lambda_{A1}(t) = \lambda_{A2}(t)$, $H_0^{1,3} : \lambda_{A1}(t) = \lambda_{B1}(t)$, $H_0^{1,4} : \lambda_{A1}(t) = \lambda_{B2}(t)$,
   $H_0^{2,3} : \lambda_{A2}(t) = \lambda_{B1}(t)$, $H_0^{2,4} : \lambda_{A2}(t) = \lambda_{B2}(t)$, $H_0^{3,4} : \lambda_{B1}(t) = \lambda_{B2}(t)$

All alternative hypotheses are formally defined analogous to the global alternative hypothesis. The hypotheses will be tested in the order as depicted above, starting at the global level, followed by the partition/intersection level and finally testing the pairwise hypotheses. All null hypotheses will be tested confirmatory at the two-sided 2.5% significance level using the log-rank test stratified by ISS stage. Statistically significant prolonged PFS of a treatment arm with respect to a comparator arm will be concluded if the adjusted p-value of the elementary hypothesis is below 0.025.

The two primary objectives will be evaluated in separate analysis to allow for early reporting of the first primary objective. The final analysis of the first primary endpoint will be realized once all patients completed induction therapy and their data has been cleaned. A first biometrical report will be provided summarizing the first primary endpoint and toxicity during induction therapy. The scope of the first analysis does not include any preliminary analysis of the second primary objective PFS, i.e., it does not constitute a formal interim analysis and consequently no adjustment for multiplicity is necessary. All remaining analyses including the second primary analysis are performed after database closure and will be presented in the final biometrical report.

Secondary Endpoints

For time-to-event endpoints (OS) Kaplan-Meier estimates will be provided and a log-rank test performed. Response rates will be tabulated and Fisher's Exact test, Cochran-Armitage's trend test (for response categories) as well as Pearson-Clopper two-sided confidence intervals will be calculated. Multivariate analysis will be carried out using Cox regression (PFS, OS) and logistic regression (response). All secondary and exploratory analyses are not subject to adjustment of the significance level for multiplicity.

Incidence rates of adverse events will be tabulated and summarized by treatment arm, therapy cycle, body system and CTC-AE-grade, and analysed by Fisher's Exact Test, χ2-test, and the Cochran-Armitage trend test (for AE grades). (Serious) Adverse events after allogeneic transplantation (i.e. after having left the MM5 trial) only will be included in the safety analysis if considered to be related to the MM5 study treatment.

Additional Analysis

The association between gene expression profiles and efficacy endpoints (OS, PFS, response to treatment) will be analysed by developing prognostic models for high-dimensional data (e.g. L_1-penalized regression models) for binary, ordinal and survival endpoints.

12.5 Interim Analyses

One interim analysis is planned to rule out lack of efficacy for the induction regimens. Results of the interim analysis will be presented confidentially to an independent data and safety monitoring board (DSMB). The interim analysis of response rates as defined for the first primary endpoint will be conducted after the first 75 patients in each induction regimen (A1+B1, A2+B2) are evaluable. Recruitment will be continued if the $VGPR^+$ rate exceeds 30%, i.e., if at least 23 of the first 75 randomised patients in each arm will reach a VGPR or better.

At the same time, an interim analysis of the first primary endpoint will be carried out. The two-sided significance level of the first primary endpoint will be split into $\alpha=0.001$ for the interim and $\alpha=0.024$ for the final analysis.

An interim analysis of the second primary endpoint (PFS) will be performed at the same time the final analysis of the first primary endpoint is done which is approximately 42 months after start of recruitment. The two-sided significance level of the second primary endpoint will be split into $\alpha=0.001$ for the interim and $\alpha=0.024$ for the final analysis.

The study will be monitored closely based on the reported SAE's. Safety results will be provided to the DSMB and the manufacturers of the investigational products on an annual basis as part of an interim safety report. One interim safety report will be provided at the same time as the interim analysis of the first primary endpoint.

As there are data showing a potentially increased risk for secondary primary malignancies (SPM) especially after alkylating agents (like Melphalan 200 or MPR) for patients during and after long term lenalidomide treatment[100,101,102], an additional close safety monitoring for secondary malignancies will be done. Any malignancy newly diagnosed during study treatment, has to be reported as serious adverse event, i.e. within 24h after awareness (see Chapter 11). In addition to the expedited SAE reporting, any newly diagnosed malignancy during study treatment and in the follow up period has to be documented in the eCRF (throughout the entire term of the study). A continuous safety monitoring with regard to SPMs will be described in an addendum to the statistical analysis plan. The monitoring will allow to stop the study early if the SPM rates exceed specific boundaries.

12.6 Protocol Amendment No. 1 (protocol version 2.0)

Due to an expected improvement in the safety profile, the route of administration for Bortezomib will be changed from intravenous to subcutaneous as of date of implementation of this protocol version 2.0 (30.11.2011) for all patients newly randomized. Based on results from a non-inferiority randomized phase III trial[98], response rates are comparable for both administration routes, i.e. the efficacy assumptions of the ongoing trial are not affected. The modification also has no implication on the randomized group comparisons as both induction regimens switch to s.c. administration and at the same time. The analysis of all primary and secondary endpoints

remains unchanged. Additional analysis will include separate toxicity analyses for both administration routes and exploratory analyses of efficacy endpoints adjusting for effect of route of administration.

12.7 Protocol Amendment No. 2 (protocol version 3.0)

In February 2012 we changed the route of administration for Bortezomib from intravenous to subcutaneous (implementation of Amendment No. 1, protocol version 2.0, 30.11.2011). Reason for this change of application was an expected reduction of side effects of Bortezomib treatment. As described in chapter 12.6, this modification has no implication on the randomized group comparisons as both induction regimens switch to s.c. administration and at the same time. The analysis of all primary and secondary endpoints remains unchanged. Additional analysis will include separate toxicity analyses for both administration routes and exploratory analyses of efficacy endpoints adjusting for effect of route of administration.

When amendment No. 1 could have been implemented, about 300 patients had been included, so more patients received Bortezomib i.v. compared to the number of patients with s.c administration. Now, further 100 patients should be treated according to the MM5 protocol in particular to test the expected improvement in the safety profile of s.c. administration compared to i.v. administration in a comparable number of patients for descriptive statistics. The additional 100 patients should also be treated in the second part of the MM5 trial with Lenalidomide for 2 years versus Lenalidomide until CR. According to the current state of knowledge, the randomized inclusion into and treatment within the MM5 trial doesn’t lead to any drawback for the patient, but would enable the patients to receive a potent and innovative therapy within a clinical trial. Three independent prospective, randomized trials have recently shown a significant improvement of progression-free survival as a result of Lenalidomide maintenance treatment [100, 101, 102]. Based on the recruitment rate of the MM5 trial, the additional recruitment is expected to take place within 6-9 months. In case in this time there would be new information that would suggest a significant benefit of one of the treatment arms within the MM5 trial compared to the other arms or that would suggest an adverse benefit-risk-assessment for the patients due to the treatment within the MM5 trial, the treatment would be changed to the superior arm or the inclusion of additional patients would be stopped, respectively. The development of SAEs will be analyzed closely, with special focus on secondary malignancies.

The additional recruitment will not affect the initially planned analysis of the primary and secondary endpoints of the trial. The trial will be analyzed as defined in the protocol based on the date of n=504 patients. After the data of n=604 patients are available, there will be an additional descriptive analysis of the safety profile and an additional exploratory analysis of the primary/secondary endpoints.

13 Data Management

13.1 Data Collection

All study related findings including clinical and laboratory data will be documented in the subject's medical record and in the electronic CRF (e-CRF). The investigator is responsible for ensuring that all sections of the CRF are completed correctly and that entries can be verified against source data.

The system MACRO (InferMed Ltd, 2008; current version 3.082) is used for data entry and handling. Data entry is only permitted to dedicated persons who are requested to authenticate themselves by a personal password. Erroneous entries can be corrected by the same person. The system uses an audit trail to ensure that all data editing steps are logged. Every attempt to

change a previous entry triggers a request by the system for the reason to change this value. The study database is located on a central server at KKS in Mainz. Only qualified staff has physical access to the server.

Only a terminal client program is used on the site's computer. This way, no data are recorded at the site. Instead, every action by the user is transmitted to the server. All transfers are encrypted to ensure confidentiality and integrity.

13.2 Data Handling

Data entries will undergo an automatical online check for plausibility and consistency. In case of implausibilities, 'warnings' will be produced automatically. A responsible investigator (or sub-investigator) will be obliged either to correct the implausible data or to confirm its authenticity and to give appropriate explanation. If not corrected, the data will be flagged, rendering a check of all questionable entries conveniently possible. A responsible monitor will check all flagged data and will generate questions that will be sent back to the responsible investigator. Additionally, a central review of the entries regarding the response assessment will be performed by a physician authorised by the principal investigator. The investigator will have to resolve all 'discrepancies' placed by the monitor in case of implausible data

Further checks for plausibility, consistency, and completeness of the data will be performed during and after completion of the study. Queries will be generated on the basis of these checks combined with a visual control by a responsible monitor/data manager.

All data management activities will be performed according to the current Standard Operating Procedures (SOPs) of the KKS. Additionally, user guidances for the clinical data system will be used.

13.3 Storage and Archiving of Data

The investigator will archive all trial data (subject identification code list, source data and investigator's file) and relevant correspondence in the Investigator Site File (ISF). All source data and all documents itemized in section 8 of the ICH Consolidated Guideline on GCP will be archived after finalization of the trial according to the legal regulations at the investigators' sites or at the sponsor, where appropriate.

The responsibility for the archiving of the Trial Master File (TMF) including the completed e-CRFs and the final report will be with the coordinating investigator and sponsor of the trial. The KKS will provide methods to store archival copies of the eCRF along with information on times and originator of alterations made to the data base.

14 Ethical and Legal Aspects

14.1 Good Clinical Practice

The procedures set out in this trial protocol, pertaining to the conduct, evaluation, and documentation of this trial, are designed to ensure that all persons involved in the trial abide by the Good Clinical Practice Guidelines of the International Conference on Harmonisation (ICH-GCP) and the ethical principles described in the applicable version of the Declaration of Helsinki (filed in the Investigator Site File, ISF). The trial will be carried out in keeping with local legal and regulatory requirements.

14.2 Subject Information and Informed Consent

Before being admitted to the clinical trial, the subject must consent to participate after the nature, scope, and possible consequences of the clinical trial have been explained in a form

understandable to him or her. The subject must give consent in writing. The signed Informed Consent Form will be filed by the investigator.

A copy of the signed informed consent document must be given to the subject. The documents must be in a language understandable to the subject and must specify who informed the subject.

The subjects will be informed as soon as possible if new information may influence his/her decision to participate in the trial. The communication of this information should be documented.

14.3 Confidentiality

The data obtained in the course of the trial will be treated pursuant to the legal requirements (i.e., in Germany to the Federal Data Protection Law (Bundesdatenschutzgesetz)).

During the clinical trial, subjects will be identified solely by means of an individual identification code (randomisation number). Trial findings stored on a computer will be stored in accordance with local data protection law and will be handled in strictest confidence. For protection of these data, organizational procedures are implemented to prevent distribution of data to unauthorized persons. The appropriate regulations of local data legislation will be fulfilled in its entirety.

The subject consents in writing to release the investigator from his/her professional discretion in so far as to allow inspection of original data for monitoring purposes by health authorities and authorized persons (inspectors, monitors, auditors). Authorized persons (clinical monitors, auditors, inspectors) may inspect the subject-related data collected during the trial ensuring the appropriate effective data protection law.

The investigator will maintain a subject identification list (randomisation numbers with the corresponding subject names) to enable records to be identified.

Subjects who did not consent to circulate their pseudonymized data will not be included into the trial.

14.4 Responsibilities of Investigator

The investigator should ensure that all persons assisting with the trial are adequately informed about the protocol, any amendments to the protocol, the trial treatments, and their trial-related duties and functions.

The investigator should maintain a list of subinvestigators and other appropriately qualified persons to whom he or she has delegated significant trial-related duties.

14.5 Approval of Trial Protocol and Amendments

Before the start of the trial, the trial protocol, informed consent document, and any other appropriate documents will be submitted to the independent ethics committee (EC) as well as to the competent authority. A written favourable vote of the EC and an (implicit) approval by the competent higher federal authority are a prerequisite for initiation of this clinical trial. The statement of EC should contain the title of the trial, the trial code, the trial site, and a list of reviewed documents. It must mention the date on which the decision was made and must be officially signed by a committee member. This documentation must also include a list of members of the EC present on the applicable EC meeting and a GCP compliance statement.

Before the first subject is enrolled in the trial, all ethical and legal requirements must be met. All planned substantial changes (see §10, (1) of German GCP-Regulation) will be submitted to EC and the competent higher federal authority in writing as protocol amendments. They have to be approved by the EC and the competent higher federal authority.

The investigator and the KKS Heidelberg will keep a record of all communication with the EC and the regulatory authorities.

14.6 Continuous Information to Independent Ethics Committee

Persuant to the German Drug Law (AMG) and the GCP Ordinance, the EC and the competent higher federal authority will be informed of all suspected serious unexpected adverse reactions (SUSARs) and all AEs resulting in death or being live-threatening occurring during the trial. Both institutions will be informed in case the risk/ benefit assessment did change or any others new and significant hazards for subjects' safety or welfare did occur. Furthermore, a report on all observed serious adverse events (SAEs) will be submitted once a year – Annual Safety Report.

The sponsor will provide Celgene with a copy of the annual periodic safety report at the time of submission to the regulatory authority and ethics committee.

The EC and the regulatory authorities must be informed of the end of the trial. They will be provided with a summary of trial results within one year after the end of clinical phase (LPO).

14.7 Notification of Regulatory Authorities

The local regulatory authorities responsible for each particular investigator will be informed before the beginning, during and at the end of the trial according to the applicable regulations. Each investigator is obliged to notify his/ her local regulatory authority. This responsibility has been delegated to the GMMG-Studiensekretariat.

14.8 Registration of the Trial

Prior to the beginning of the clinical phase (FPI) the coordinating investigator will register the trial at Current Controlled Trials (http://www.controlled-trials.com/). Thus the trial will be given a unique ISRCTN, which is a prerequisite for a publication in a peer-review paper.

14.9 Insurance

According to § 40 AMG, the sponsor has to subscribe to an insurance policy covering, in its terms and provisions, its legal liability for injuries caused to participating persons and arising out of this research performed strictly in accordance with the scientific protocol as well as with applicable law and professional standards. The insurance was taken out at "HDI-Gerling Industrie Versicherung AG" (insurance number:57 010310 03018, maximum limit: € 500.000,-- per participating person). This insurance – as part of the insurance program of the university hospital Heidelberg - covers all patients from participating centers in Germany. For participating centers in France a separate insurance will be contracted. The sponsor will ensure that insurance is in place for all participating sites.

Any impairment of health which might occur in consequence of trial participation must be notified to the insurance company. The subject is responsible for notification. The insured person will agree with all appropriate measures serving for clarification of the cause and the extent of damage as well as the reduction of damage.

During the conduct of the trial, the subject must not undergo other clinical treatment except for cases of emergency. The subject is bound to inform the investigator immediately about any adverse events and additionally drugs taken. The terms and conditions of the insurance should be delivered to the subject.

The insurance company has to be informed about all amendments that could affect subjects' safety.

15 Quality Assurance

15.1 Monitoring

Monitoring will be done by personal visits from a clinical monitor according to SOPs of the KKS. The monitor will review the entries into the CRFs on the basis of source documents (source data verification). The investigator must allow the monitor to verify all essential documents and must provide support at all times to the monitor.

By frequent communications (letters, telephone, fax), the site monitor will ensure that the trial is conducted according to the protocol and regulatory requirements.

Monitoring details will be specified in a monitoring manual.

15.2 Inspections/ Audits

Regulatory authorities and an auditor authorized by the sponsor may request access to all source documents, CRF, and other trial documentation. Direct access to these documents must be guaranteed by the investigator who must provide support at all times for these activities.

16 Agreements

16.1 Financing of the Trial

The MM5 trial is a non-commercial, investigator-initiiated trial. The trial will be co-financed using funds of Celgene, OrthoBiotech, Chugai. The study drug lenalidomide will be provided by Celgene. The study drug bortezomib was provided by Ortho Biotech for the first 504 patients included into the trial. The Binding Site will provide kits in order to perform Freelite® and Hevylite®-Tests.

16.2 Financial Disclosure

Before the start of the trial, the investigator will disclose to the sponsor any proprietary or financial interests he or she might hold in a funding company, in the investigational product(s) or any commercial organisation being involved in the clinical trial. The investigator has also to confirm that he/she has not entered into any financial arrangement, whereby the value of compensation paid could affect the outcome of the clinical trial.

The investigator agrees to update this information in case of significant changes.

16.3 Reports

The Division of Biostatistics, German Cancer Research Center, already prepared a report for the interim analyses in Q1 2012. A biometrical report with respect to the first primary endpoint will be prepared in Q3 2013 and the final biometrical report within 3 months after closure of data base (approximately in Q4 2017), the principal investigator will prepare the final trial report approximately in Q1 2018.

16.4 Publication

All information concerning the trial is confidential before publication.

The final publications of the trial results will be coordinated by the principal investigator on the basis of the statistical analysis performed by the Division of Biostatistics, German Cancer Research Center. A draft manuscript will be submitted to all co-authors and representatives from companies providing grants for the conduct of the trial for review. After revision by the co-authors and the representatives from the companies within a maximum of 60 days, the manuscript will be sent to a peer reviewed scientific journal.

Authors of the manuscript will include the trial coordinator, investigators who have included more than 5% of the evaluable patients in the trial (by order of inclusion), the statistician(s) and the datamanager in charge of the trial and others who have made significant scientific contributions.

Interim publications or presentations of the study may include demographic data, overall results and prognostic factor analyses, but no comparisons between randomised treatment arms may be made publicly available before recruitment is completed or discontinued.

Any publication, abstract or presentation based on patients included in this study must be approved by the principal investigator. This is applicable to any individual patient randomised in the trial, or any subgroup of the trial patients. Such a publication cannot include any comparisons between randomised treatment arms nor an analysis of any of the study end-points unless the final results of the trial have already been published.

17 Signatures

The present trial protocol was subject to critical review and has been approved in the present version by the persons undersigned. The information contained is consistent with:

- the current risk-benefit assessment of the investigational medicinal product
- the moral, ethical, and scientific principles governing clinical research as set out in the applicalble version of Declaration of Helsinki and the principles of GCP.

The investigator will be supplied with details of any significant or new finding including AEs relating to treatment with the investigational medicinal product.

Date: 7. 6. 13 Signature: [signature]
Name (block letters): Prof. Dr. med. Hartmut Goldschmidt
Function: Principal Investigator and Sponsor's representative

Date: 07.06.2013 Signature: [signature]
Name (block letters): Dr. Christina Kunz
Function: Biometrician

Date: 06.06.2013 Signature: [signature]
Name (block letters): Dr. med. Uta Bertsch
Function: Trial Coordinator

Date: 07.06.2013 Signature: [signature]
Name (block letters) : Dr. med. Steffen Luntz
Function: Head of KKS Heidelberg

18 Declaration of Investigator

I have read the above trial protocol and confirm that it contains all information to conduct the clinical trial. I pledge to conduct the clinical trial according to the protocol.

I will enroll the first subject only after all ethical and regulatory requirements are fulfilled. I pledge to obtain written consent for trial participation from all subjects.

I know the requirements for accurate notification of serious adverse events and I pledge to document and notify such events as described in the protocol.

I pledge to retain all trial-related documents and source data as described. I will provide a Curriculum Vitae (CV) before trial start. I agree that the CV may be submitted to the responsible regulatory authorities. I agree that the notification to the competent authorities (according to §67 AMG and §12 (1) of the GCP Ordinance) will be done by the sponsor or on behalf of the sponsor's representative.

Date ____________ Signature: ________________________________

Name (block letters): ________________________________

Function: Investigator

Trial Center (address): ________________________________

____

____

19 Appendices

Appendix I - Diagnostic Criteria for Multiple Myeloma

Appendix II - Response Criteria (3 pages)

Appendix III - A - WHO Performance Status /
B - NYHA Functional Classification

Appendix IV - Required Investigations (Schedule)

Appendix V - CTC Grading of Polyneuropathy according to CTCAE v4.0

Appendix VI - Management of Patients with Bortezomib (Velcade®)-related Neuropathic Pain and/or Peripheral Sensory Neuropathy

Appendix VII References

Further relevant documents can be found in the Investigator Site File (ISF):

- Declaration of Helsinki
- Accompanying forms and instructions for sample shipment, etc. see Investigator Site File (ISF).

APPENDIX I: Diagnostic Criteria

Diagnostic Criteria for Multiple Myeloma Requiring Systemic Therapy[1]

Criteria
Presence of an M-component[a] in serum and/or urine plus clonal plasma cells in the bone marrow and/or a documented clonal plasmacytoma<br>**PLUS** one or more of the following:[b]<br>**C**alcium elevation (>11.5 mg/dl) [>2.65 mmol/l]<br>**R**enal insufficiency (creatinine >2 mg/dl) [177 µmol/l or more]<br>**A**nemia (hemoglobin <10 g/dl or 2 g/dl <normal)<br>(haemoglobin <6,21 mmol/l[c] or 1.24mmol/l < normal)<br>**B**one disease (lytic lesions or myeloma-related osteopenia/osteoporosis)

[a] In patients with no detectable M-component, an abnormal serum FLC ratio on the serum FLC assay can substitute and satisfy this criterion.
For patients, with no serum or urine M-component and normal serum FLC ratio, the baseline bone marrow must have ≥ 10% clonal plasma cells; these patients are referred to as having ‘non-secretory myeloma’ **(please note: these patients with non-secretory myeloma are NOT eligible for this trial)**.

[b] Must be attributable to the underlying plasma cell disorder.

[c] *Note*: Hemoglobin of 10 g/dl is 6,21 mmol/l [or 100 g/l].

APPENDIX II: Response Criteria

Response will be assessed according to the International Myeloma Working Group Uniform (IMWG) Response Criteria[1]. In addition and modification to the IMWG criteria "minimal response" (MR) as defined in the EBMT criteria[95] , "near CR" (nCR) and "molecular CR" (mCR) have been added.

Appendix II – table 1: Response Subcategories

Response subcategory	Response criteria
*mCR^{PCR} **	*mCR^{PCR} is defined as mCR^{FACS} plus absence of detectable clonal plasma cells in ASO-PCR. Note: categorization as mCR will not be performed by the investigator*
*mCR^{FACS} **	*mCR^{FACS} is defined as sCR plus absence of a detectable malignant plasma cell population in flow cytometric investigation of whole bone marrow. Note: categorization as mCR will not be performed by the investigator.*
sCR	CR as defined below plus<br>Normal FLC ratio and<br>Absence of clonal cells in bone marrow[b] by immunohistochemistry or immunofluorescence[c]
CR	Negative immunofixation on the serum and urine and<br>Disappearance of any soft tissue plasmacytomas and<br>< 5% plasma cells in bone marrow
*nCR**	*subcategory of VGPR defined as absence of serum and urine M-Protein on standard electrophoresis and/or standard 24h urinary measurement with a positive immunofixation status in serum and/or urine*<br>*(categorization as nCR does not need to be done by investigator since subdivision of VGPR is self-evident based on the entries for M-Protein and immunofixation and will be done centrally).*
VGPR	Serum and urine M-protein detectable by immunofixation but not on electrophoresis or 90% or greater reduction in serum M-protein plus urine M-protein level < 100mg per 24h
PR	≥ 50% reduction of serum M-protein and reduction in 24-h urinary M-protein by ≥ 90% or to ≤ 200mg per 24 h<br>If the serum and urine M-protein are unmeasurable,[d] a ≥ 50% decrease in the difference between involved and uninvolved FLC levels is required in place of the M-protein criteria<br>In addition to the above listed criteria, if present at baseline, a ≥ 50% reduction in the size of soft tissue plasmacytomas is also required
MR*	MR requires all of the following:<br>25-49% reduction in the level of the serum monoclonal paraprotein<br>50-89% reduction in 24h urinary light chain excretion, which still exceeds 200mg/24h<br>25-49% reduction in the size of soft tissue plasmocytoma (by radiography or clinical examination)
SD	Not meeting criteria for CR, VGPR, PR, MR or progressive disease

Abbreviations: CR, complete response; FLC, free light chain; PR, partial response; mCR, molecular complete response; MR, minimal response; nCR, near complete response; SD, stable disease; sCR, stringent complete response; VGPR, very good partial response.

[a]All categories require no known evidence of progressive or new bone lesions if radiographic studies were performed. Radiographic studies are not required to satisfy these response requirements.

*Consecutive assessments of response before the next treatment phase of the protocol are not required (due to the short treatment intervals e.g., after induction treatment)**

[b]Confirmation with repeat bone marrow biopsy not needed.

[c]Presence/absence of clonal cells is based upon the k/l ratio. An abnormal k/l ratio by immunohistochemistry and/or immunofluorescence requires a minimum of 100 plasma cells for analysis. An abnormal ratio reflecting presence of an abnormal clone is k/l of > 4:1 or < 1:2.

[d]definitions of measurable disease see Appendix II, table 3

* modification to the IMWG criteria[1]

Appendix II – table 2: Definition of Progressive Disease

Relapse subcategory	Relapse criteria
Progressive Disease [a]	Progressive Disease: requires any one or more of the following:<br>Increase of ≥ 25% from lowest response level in<br>Serum M-component and/or (the absolute increase must be ≥ 5 g/l)[b]<br>Urine M-component and/or (the absolute increase must be ≥ 200 mg/24 h<br>Only in patients without measurable serum and urine M-protein levels: the difference between involved and uninvolved FLC levels. The absolute increase must be > 10 mg/dl.<br>Bone marrow plasma cell percentage: the absolute % must be ≥ 10%[c]<br>Definite development of new bone lesions or soft tissue plasmacytomas or definite increase in the size of existing bone lesions or soft tissue plasmacytomas<br>Development of hypercalcemia (corrected serum calcium > 11.5 mg/dl or 2.65 mmol/l) that can be attributed solely to the plasma cell proliferative disorder
Clinical relapse	Clinical relapse requires one or more of:<br>Direct indicators of increasing disease and/or end organ dysfunction (CRAB features)[b]. It is not used in calculation of time to progression or progression-free survival but is listed here as as something that can be reported optionally or for use in clinical practice<br>1. Development of new soft tissue plasmacytomas or bone lesions<br>2. Definite increase in the size of existing plasmacytomas or bone lesions. A definite increase is defined as a 50% (and at least 1 cm) increase as measured serially by the sum of the products of the cross-diameters of the measurable lesion<br>3. Hypercalcemia (>11.5 mg/dl) [2.65 mmol/l]<br>4. Decrease in hemoglobin of ≥ 2 g/dl [1.25 mmol/l] (see appendix I for further details)<br>5. Rise in serum creatinine by 2 mg/dl or more [177 µmol/l or more]
Relapse from CR[a] (<u>not</u> used for PFS calculation in this trial) [d]	Any one or more of the following:<br>- Reappearance of serum or urine M-protein by immunofixation or electrophoresis<br>- Development of ≥ 5% plasma cells in the bone marrowc<br>- Appearance of any other sign of progression (i.e., new plasmacytoma, lytic bone lesion, or hypercalcemia see below)

[a] All relapse categories require two consecutive assessments made at anytime before classification as relapse or disease progression and/or the institution of any new therapy.

[b] For progressive disease, serum M-component increases of ≥ 10g/l are sufficient to define relapse if starting M-component is ≥ 50 g/l.

[c] Relapse from CR has the 5% cutoff versus 10% for other categories of relapse.

[d] For purposes of calculating progression-free survival, CR patients should also be evaluated using criteria listed above for progressive disease.

Appendix II – table 3: Practical Details of Response Evaluation

**Practical details of response evaluation**
**Laboratory tests for measurement of M-protein**<br>Serum M-protein level is quantitated using densitometry on SPEP except in cases where the SPEP is felt to be unreliable such as in patients with IgA monoclonal proteins migrating in the beta region. If SPEP is not available or felt to be unreliable (e.g., in some cases of IgA myeloma) for routine M-protein quantitation during therapy, then quantitative immunoglobulin levels on nephelometry or turbidometry can be accepted. However, this must be explicitly reported, and only nephelometry can be used for that patient to assess response and SPEP and nephelometric values cannot be used interchangeably.<br>Urine M-protein measurement is estimated using 24-h urine only. *24h UPEP or 24 h urine tests measuring kappa and lambda light chain levels are permitted* (note: an adequate test for urine light chain levels must be used, tests for serum light chain measurement cannot be used for urine)*
**Definitions of measurable disease**<br>Response criteria for all categories and subcategories of response except CR are applicable only to patients who have 'measurable' disease defined by at least one of the following three measurements:<br>Serum M-protein ≥ 10 g/l<br>Urine M-protein ≥ 200 mg/24 h<br>Serum FLC assay: Involved FLC level ≥ 10 mg/dl (≥ 100 mg/l) provided serum FLC ratio is abnormal<br>Response criteria for CR are applicable for patients who have abnormalities on one of the three measurements.<br>Note that patients who do not meet any of the criteria for measurable disease as listed above cannot be included in the trial.*
**Follow-up to meet criteria for PR or SD**<br>Patients with 'measurable disease' as defined above need to be followed by both SPEP and UPEP for response assessment and categorization.<br>Except for assessment of CR, patients with measurable disease restricted to the SPEP will need to be followed only by SPEP; correspondingly, patients with measurable disease restricted to the UPEP will need to be followed only by UPEP[a]<br>Patients with measurable disease in either SPEP or UPEP or both will be assessed for response only based on these two tests and not by the FLC assay. FLC response criteria are only applicable to patients without measurable disease in the serum or urine, and to fulfill the requirements of the category of stringent CR<br>To be considered CR, both serum and urine immunofixation must be carried out and be negative regardless of the size of baseline M-protein in the serum or urine; patients with negative UPEP values pretreatment still require UPEP testing to confirm CR and exclude light chain or Bence–Jones escape

Abbreviations: CR, complete response; FLC, free light chain; PR, partial response; SD, stable disease; SPEP, serum protein electrophoresis; UPEP, urine protein electrophoresis.
[a]For good clinical practice patients should be periodically screened for light chain escape with UPEP or serum FLC assay.

* modification to the IMWG criteria[1]

APPENDIX III: WHO Performance Status and NYHA Classification

A - WHO Performance Status (ECOG[96]/WHO/Zubrod score)

Grade	Description
0	Asymptomatic<br>(Fully active, able to carry on all predisease activities without restriction)
1	Symptomatic but completely ambulatory<br>(Restricted in physically strenuous activity but ambulatory and able to carry out work of a light or sedentary nature, e.g., light house work, office work)
2	Symptomatic, <50% in bed during the day<br>(Ambulatory and capable of all self care but unable to carry out any work activities. Up and about more than 50% of waking hours)
3	Symptomatic, >50% in bed, but not bedbound<br>(Capable of only limited self-care, confined to bed or chair 50% or more of waking hours)
4	Bedbound<br>(Completely disabled. Cannot carry on any self-care. Totally confined to bed or chair)
5	Dead

B - New York Heart Association (NYHA) Functional Classification[97]

NYHA class	Functional capacity
I	Patients with cardiac disease but without resulting limitation of physical activity. Ordinary physical activity does not cause undue fatigue, palpitation, dyspnea, or anginal pain.
II	Patients with cardiac disease resulting in slight limitation of physical activity. They are comfortable at rest. Ordinary physical activity results in fatigue, palpitation, dyspnea, or anginal pain.
III	Patients with cardiac disease resulting in marked limitation of physical activity. They are comfortable at rest. Less than ordinary activity causes fatigue, palpitation, dyspnea, or anginal pain
IV	Patients with cardiac disease resulting in inability to carry on any physical activity without discomfort. Symptoms of heart failure or the anginal syndrome may be present even at rest. If any physical activity is undertaken, discomfort is increased.

APPENDIX IV: Required Investigations

Please note: before start of each treatment cycle and during the cycles, **routine investigations** like blood cell count and renal function have to be performed according to local policy

	on study	after Ind 3	IT*	prior to R	prior to MT	3 mo MT	6 mo MT	9 mo MT	12 mo MT	15 mo MT	18 mo MT	21 mo MT	24 mo MT	at relapse	comment
**to be performed locally**															
medical history**	X	X	X	X	X	X	X	X	X	X	X	X	X		
physical examination**	X	X	X	X	X	X	X	X	X	X	X	X	X		
hematology**	X	X	X	X	X	X	X	X	X	X	X	X	X		additionally: during lenalidomide weekly (first 8 weeks) / monthly (afterwards)
IgA, IgG, IgM, (IgD)	X	X	X	X	X	X	X	X	X	X	X	X	X		
SPEP	X	X	X	X	X	X	X	X	X	X	X	X	X		
Total proteins	X	X	X	X	X	X	X	X	X	X	X	X	X		
immunofixation serum	X	X	X	X	X	X	X	X	X	X	X	X	X		during treatment: mandatory if no M-peak in SPEP
immunofixation urine	X	X	X	X	X	X	X	X	X	X	X	X	X		mandatory if light chain excretion in 24h urine within normal ranges (or suspected unspecific inrease)
light chains in urine (24h urin)	X	X	X	X	X	X	X	X	X	X	X	X	X		
bone marrow	X	at any time to confirm a "CR" (as soon as a CR might be present***; if a CR is confirmed (PC < 5%), repetition necessary for MRD (see below))													
blood/urine chemistry**	X	X	X	X	X	X	X	X	X	X	X	X	X		
serum β2-microglobulin	X														
Creatinin clearance (24h urine)	X														further investigations on clinical indication
calculation of Creatinin clearance			(X)	X	X	X	X	X	X	X	X	X	X		MDRD formula (needed parameter: serum creatinine, age, sex, ethnicity)
x-ray thorax	X		X												
ECG	X	X													if "on study" and after "Ind 3" no pathological findings: further investigations only on clinical indication
Cardiac echo	X	X													
Imaging (recommended technique see 9.3)	X		(X)	X											further investigations only if symptoms occur or serological progression is present
HCG pregnancy test	X			before lenalidomide (len.), every 4 weeks during len., 4 weeks after len.											only for women of childbearing potential
**to be sent in (central diagnostic)**															
BM aspirate (80ml) for iFISH, GEP	X												X (only 20ml)	X	
Serum 2x7.5 ml (free-/hevylite)	X	X	X	X	X	X	X	X	X	X	X	X	X	X	
Peripheral Blood 20ml	X	X		X	X								X	X	
BM aspirate (15ml) for analyses for minimal residual disease (MRD)		timepoint depends on course and lab findings. Bone marrow aspirate (15 ml) für MRD diagnostic has to be sent in<br>1.) when a bone marrow puncture is performed routinely to confirm CR (aspirate has to be analysed locally, see above, + additional sample should be sent in)<br>2.) after FLC test has shown a normal FLC ratio (at next visit)<br>3.) 6 months after 2.)													

* "IT" = after each chemotherapy cycle of intensification regime; (X) only applicable "prior to R" (also see next column)

** details on required data (as also listed in chapter 9.3):

medical history: standard medical history, with special attention for WHO performance status, infections, polyneuropathy
("on study" additionally: prior and present other diseases, previous chemotherapy or radiotherapy, childbearing potential)

physical examination: standard physical examination incl. body weight and height, with special attention for polyneuropathy or other neurologic symptoms, infections

hematology: hemoglobin, leukocyte count (differential count), platelets

blood chemistry: total proteins, albumin, creatinine, *GOT,* GPT, *γ-GT*, *urea, total bilirubin, alkaline phosphatase,* LDH, CRP, calcium, *sodium, potassium, uric acid*

urine chemistry: total proteins in 24h urine

***CR might be present as soon as no more monoclonal protein is detectable by immunofixation (immunofixation serum and urine negative).
In this case a bone marrow puncture to confirm CR has to be done as soon as possible, before the next treatment phase starts

APPENDIX V: CTC Grading of Polyneuropathy according to CTCAE v4.0

Adverse event	Grade 1	Grade 2	Grade 3	Grade 4	Grade 5
**Paresthesia**	Mild symptoms	Moderate symptoms; limiting instrumental ADL*	severe symptoms; limiting self care ADL	-	-
Definition: A disorder characterized by functional disturbances of sensory neurons resulting in abnormal cutaneous sensations of tingling, numbness, pressure, cold, and warmth that are experienced in the absence of a stimulus.					
**Peripheral motor neuropathy**	Asymptomatic, clinical or diagnostic observations only; intervention not indicated	Moderate symptoms; limiting instrumental ADL	severe symptoms; limiting self care ADL; assistive device indicated	Life-threatening consequences; urgent intervention indicated	Death
Definition: A disorder characterized by inflammation or degeneration of the peripheral motor nerves.					
**Peripheral sensory neuropathy**	Asymptomatic; loss of deep tendon reflexes or paresthesia	Moderate symptoms; limiting instrumental ADL	severe symptoms; limiting self care ADL	Life-threatening consequences; urgent intervention indicated	Death
Definition: A disorder characterized by inflammation or degeneration of the peripheral sensory nerves					

***Activities of Daily Living (ADL)**

- Instrumental ADL refer to preparing meals, shopping for groceries or clothes, using the telephone, managing money, etc.

- Self care ADL refer to bathing, dressing and undressing, feeding self, using the toilet, taking medications, and not bedridden.

APPENDIX VI: Management of Patients with Bortezomib (Velcade®)-related Neuropathic Pain and/or Peripheral Sensory Neuropathy

			**Peripheral sensory neuropathy (CTCAE grade, v3.0)**				
			**0**	**1**	**2**	**3**	**4**
			**normal**	**Asymptomatic; loss of deep tendon reflexes or paresthesia (including tingling) but not interfering with function**	**Sensory alteration or paresthesia (including tingling), interfering with function, but not interfering with ADL**	**Sensory alteration or paresthesia interfering with ADL**	**Disabling**
**Neuropathic pain (CTCAE grade; v3.0)**	**0**	**None**	No action	No action	25% dose reduction	Hold, 50% dose reduction; schedule Δ required	Discontinue bortezomib
	**1**	**Mild pain, not interfering with function**	No action	No action	25% dose reduction	Hold, 50% dose reduction; schedule Δ required	Discontinue bortezomib
	**2**	**Moderate pain; pain or analgetics interfering with function, but not daily activities**	25% dose reduction	50% dose reduction	Hold; 50% dose reduction	Hold, 50% dose reduction; schedule Δ required	Discontinue bortezomib
	**3**	**Severe pain; pain or analgetics severly interfering with daily activities**	Hold, 50% dose reduction; schedule Δ required	Hold, 50% dose reduction; schedule Δ required	Hold, 50% dose reduction; schedule Δ required	Discontinue bortezomib	Discontinue bortezomib
	**4**	**Disabling**	Discontinue bortezomib	Discontinue bortezomib	Discontinue bortezomib	Discontinue bortezomib	Discontinue bortezomib

Key:

- Hold: Interrupt bortezomib for up to 2 weeks until the toxicity returns to Grade 1 or better.

- 25% Dose reduction: bortezomib dose reduction from 1.3 to 1.0 mg/m^2/dose.

- 50% Dose reduction: bortezomib dose reduction from 1.3 to 0.7 mg/m^2/dose.

- Schedule Δ Required: Schedule change from bortezomib twice per week (Days 1, 4, 8 and 11) to once per week (Days 1 and 8) required. If the patient is already on a once weekly schedule, then the drug will be given every other week (e.g., Day 1, Day 15).

Reference List

1. Durie BG, Harousseau JL, Miguel JS et al. International uniform response criteria for multiple myeloma. Leukemia. 2006;20:1467-1473.

2. Harousseau JL, Moreau P. Autologous hematopoietic stem-cell transplantation for multiple myeloma. N.Engl.J Med. 2009;360:2645-2654.

3. Palumbo A, Dimopoulos MA, Delforge M et al. A Phase III Study to Determine the Efficacy and Safety of Lenalidomide in Combination with Melphalan and Prednisone (MPR) in Elderly Patients with Newly Diagnosed Multiple Myeloma. ASH Annual Meeting Abstracts 2009;114:613.

4. Alexanian R, Barlogie B, Tucker S. VAD-based regimens as primary treatment for multiple myeloma. Am.J Hematol. 1990;33:86-89.

5. Goldschmidt H, Sonneveld P, Breitkreuz I et al. HOVON 50/GMMG-HD3-Trial: Phase III Study on the Effect of Thalidomide Combined with High Dose Melphalan in Myeloma Patients up to 65 Years. ASH Annual Meeting Abstracts 2005;106:424 (updated data, oral presentation at ASH).

6. Sonneveld P, Eljarari L, Salwender H et al. First Analysis of HOVON-65/GMMG-HD4 Randomized Phase III Trial Comparing Bortezomib, Doxorubicin, Dexamethasone (PAD) vs. VAD as Induction Treatment Prior to High-dose Melphalan (HDM) in Patients with Newly Diagnosed Multiple Myeloma (MM) [abstract]. Clinical Lymphoma & Myeloma 2009;9:S105 (abstract B152).

7. Cavo M, Zamagni E, Tosi P et al. Superiority of thalidomide and dexamethasone over vincristine-doxorubicindexamethasone (VAD) as primary therapy in preparation for autologous transplantation for multiple myeloma. Blood 2005;106:35-39.

8. Rajkumar SV, Rosinol L, Hussein M et al. Multicenter, randomized, double-blind, placebo-controlled study of thalidomide plus dexamethasone compared with dexamethasone as initial therapy for newly diagnosed multiple myeloma. J Clin Oncol 2008;26:2171-2177.

9. Goldschmidt H, Sonneveld P, Cremer FW et al. Joint HOVON-50/GMMG-HD3 randomized trial on the effect of thalidomide as part of a high-dose therapy regimen and as maintenance treatment for newly diagnosed myeloma patients. Ann.Hematol. 2003;82:654-659.

10. Lokhorst H, van der Holt B, Zweegman S et al. Final Analysis of HOVON-50 Randomized Phase III Study on the Effect of Thalidomide Combined with Adriamycine, Dexamethasone (AD) and High Dose Melphalan (HDM) in Patients with Multiple Myeloma (MM). ASH Annual Meeting Abstracts 2008;112:157.

11. Attal M. Current and future IFM trials - Oral Presentation, International Myeloma Workshop, Washington. 2009.
Ref Type: Slide

12. Sonneveld, P. First analysis of HOVON-65/GMMG-HD4 randomized phase III trial comparing Bortezomib, Adriamycine, Dexamethasone (PAD) vs VAD as induction treatment prior to High Dose Melphalan (HDM) in patients with multiple myeloma (MM). Oral Presentation, International Myeloma Workshop, Washington. 2009.
Ref Type: Slide

13. Davies FE, Wu P, Jenner M et al. The combination of cyclophosphamide, velcade and dexamethasone induces high response rates with comparable toxicity to velcade alone and velcade plus dexamethasone. Haematologica 2007;92:1149-1150.

14. Reeder CB, Reece DE, Kukreti V et al. Cyclophosphamide, bortezomib and dexamethasone induction for newly diagnosed multiple myeloma: high response rates in a phase II clinical trial. Leukemia 2009

15. Knop S, Liebisch P, Wandt H et al. Bortezomib, IV cyclophosphamide, and dexamethasone (VelCD) as induction therapy in newly diagnosed multiple myeloma: Results of an interim analysis of the German DSMM Xia trial. [abstract]. J Clin Oncol 2009;27:

16. Rajkumar SV, Jacobus S, Callander N et al. A Randomized Trial of Lenalidomide Plus High-Dose Dexamethasone (RD) Versus Lenalidomide Plus Low-Dose Dexamethasone (Rd) in Newly Diagnosed Multiple Myeloma (E4A03): A Trial Coordinated by the Eastern Cooperative Oncology Group. ASH Annual Meeting Abstracts 2007;110:74.

17. Rajkumar SV, Jacobus S, Callander NS et al. Lenalidomide plus high-dose dexamethasone versus lenalidomide plus low-dose dexamethasone as initial therapy for newly diagnosed multiple myeloma: an open-label randomised controlled trial. Lancet Oncol. 2009

18. Durie BG, Kyle RA, Belch A et al. Myeloma management guidelines: a consensus report from the Scientific Advisors of the International Myeloma Foundation. Hematol.J 2003;4:379-398.

19. Barosi G, Boccadoro M, Cavo M et al. Management of multiple myeloma and related-disorders: guidelines from the Italian Society of Hematology (SIE), Italian Society of Experimental Hematology (SIES) and Italian Group for Bone Marrow Transplantation (GITMO). Haematologica 2004;89:717-741.

20. Attal M, Harousseau JL, Leyvraz S et al. Maintenance therapy with thalidomide improves survival in patients with multiple myeloma. Blood 2006;108:3289-3294.

21. Spencer A, Prince HM, Roberts AW et al. Consolidation therapy with low-dose thalidomide and prednisolone prolongs the survival of multiple myeloma patients undergoing a single autologous stem-cell transplantation procedure. J Clin Oncol 2009;27:1788-1793.

22. Barlogie B, Tricot G, Anaissie E et al. Thalidomide and hematopoietic-cell transplantation for multiple myeloma. N.Engl.J Med. 2006;354:1021-1030.

23. Barlogie B, Pineda-Roman M, van RF et al. Thalidomide arm of Total Therapy 2 improves complete remission duration and survival in myeloma patients with metaphase cytogenetic abnormalities. Blood 2008;112:3115-3121.

24. Morgan GJ, Jackson GH, Davies FE et al. Maintenance Thalidomide May Improve Progression Free but Not Overall Survival; Results from the Myeloma IX Maintenance Randomisation. ASH Annual Meeting Abstracts 2008;112:656.

25. Owen RG, Child JA, Jackson GH et al. MRC Myeloma IX: Preliminary results from the intensive pathway study. Clinical Lymphoma & Myeloma 2009;9:S3-S95 (abstract 546).

26. Owen RG, Morgan GJ, Jackson H et al. MRC Myeloma IX: Preliminary results from the non-intensive study. Clinical Lymphoma & Myeloma 2009;9:S3-S95 (abstract 547).

27. Ludwig H, Hajek R, Tothova E et al. Thalidomide-dexamethasone compared with melphalan-prednisolone in elderly patients with multiple myeloma. Blood 2009;113:3435-3442.

28. Ludwig H, Zdenek A, Tothova E et al. Thalidomide/Interferon versus interferon maintenance after thal/dex versus MP. Clinical Lymphoma & Myeloma 2009;9:S3-S95 (abstract 147).

29. Palumbo A, Cavallo F, Pagliano G et al. Post transplant consolidation with bortezomib, thalidomide and dexamethasone induces a clinically significant shrinkage of residual tumor burden measured by real time PCR [abstract]. Haematologica 2009;94:431 (abstract 1071).

30. Benevolo G, Larocca A, Pregno P et al. Use of maintenance therapy with bortezomib and dexamethasone (VD) in advanced multiple myeloma [abstract]. Hematol.J 2009;94:

31. Attal M, Harousseau JL, Marit G et al. Lenalidomide After Autologous Transplantation for Myeloma: First Analysis of a Prospective, Randomized Study of the Intergroupe Francophone Du Myelome (IFM 2005 02). ASH Annual Meeting Abstracts 2009;114:529.

32. Palumbo A, Falco P, Gay F et al. Bortezomib-Doxorubicin-Dexamethasone as Induction Prior to Reduced Intensity Autologous Transplantation Followed by Lenalidomide as Consolidation/Maintenance in Elderly Untreated Myeloma Patients. ASH Annual Meeting Abstracts 2008;112:159.

33. Carbone PP, Kellerhouse LE, Gehan EA. Plasmacytic myeloma. A study of the relationship of survival to various clinical manifestations and anomalous protein type in 112 patients. Am.J Med. 1967;42:937-948.

34. Costa G, Engle RL, Taliente P. Criteria for defining risk groups and response to chemotherapy in multiple myeloma. abstract. Proceedings of the American Association of Cancer Research 1969;10:

35. Kyle RA, Elveback LR. Management and prognosis of multiple myeloma. Mayo Clin.Proc. 1976;51:751-760.

36. Peest D, Coldewey R, Deicher H et al. Prognostic value of clinical, laboratory, and histological characteristics in multiple myeloma: improved definition of risk groups. Eur.J Cancer. 1993;29A:978-983.

37. Andersen NF, Standal T, Nielsen JL et al. Syndecan-1 and angiogenic cytokines in multiple myeloma: correlation with bone marrow angiogenesis and survival. Br.J.Haematol. 2005;128:210-217.

38. Greipp PR, San Miguel J, Durie BG et al. International staging system for multiple myeloma. J.Clin.Oncol. 2005;23:3412-3420.

39. Child JA, Crawford SM, Norfolk DR et al. Evaluation of serum beta 2-microglobulin as a prognostic indicator in myelomatosis. Br.J.Cancer 1983;47:111-114.

40. Cuzick J, Cooper EH, MacLennan IC. The prognostic value of serum beta 2 microglobulin compared with other presentation features in myelomatosis. Br.J Cancer. 1985;52:1-6.

41. Cuzick J, De Stavola BL, Cooper EH, Chapman C, MacLennan IC. Long-term prognostic value of serum beta 2 microglobulin in myelomatosis. Br.J Haematol. 1990;75:506-510.

42. Barlogie B, Anaissie EJ, van Rhee F et al. Total therapy (TT) for myeloma (MM)--10% cure rate with TT1 suggested by >10yr continuous complete remission (CCR): Bortezomib in TT3 overcomes poor-risk associated with T(4;14) and DelTP53 in TT2. J Clin Oncol (Meeting Abstracts) 2008;26:8516.

43. Knop S, Gerecke C, Liebisch P et al. Lenalidomide, adriamycin, and dexamethasone (RAD) in patients with relapsed and refractory multiple myeloma: a report from the German Myeloma Study Group DSMM (Deutsche Studiengruppe Multiples Myelom). Blood 2009;113:4137-4143.

44. Chiecchio L, Protheroe RK, Ibrahim AH et al. Deletion of chromosome 13 detected by conventional cytogenetics is a critical prognostic factor in myeloma. Leukemia. 2006;20:1610-1617.

45. Avet-Loiseau H, Attal M, Moreau P et al. A Comprehensive Analysis of Cytogenetic Abnormalities in Myeloma: Results of the FISH Analysis of 1000 Patients Enrolled in the IFM99 Trials. abstract. Blood 2006;106:185a.

46. Keats JJ, Reiman T, Maxwell CA et al. In multiple myeloma, t(4;14)(p16;q32) is an adverse prognostic factor irrespective of FGFR3 expression. Blood 2003;101:1520-1529.

47. Fonseca R, Blood E, Rue M et al. Clinical and biologic implications of recurrent genomic aberrations in myeloma. Blood 2003;101:4569-4575.

48. Moreau P, Facon T, Leleu X et al. Recurrent 14q32 translocations determine the prognosis of multiple myeloma, especially in patients receiving intensive chemotherapy. Blood 2002;100:1579-1583.

49. Chang H, Sloan S, Li D et al. The t(4;14) is associated with poor prognosis in myeloma patients undergoing autologous stem cell transplant. Br.J Haematol. 2004;125:64-68.

50. Hanamura I, Stewart JP, Huang Y et al. Frequent gain of chromosome band 1q21 in plasma-cell dyscrasias detected by fluorescence in situ hybridization: incidence increases from MGUS to relapsed myeloma and is related to prognosis and disease progression following tandem stem-cell transplantation. Blood. 2006;108:1724-1732.

51. Avet-Loiseau H, Attal M, Moreau P et al. Genetic abnormalities and survival in multiple myeloma: the experience of the Intergroupe Francophone du Myelome. Blood. 2007;109:3489-3495.

52. Neben K, Jauch A, Hose D et al. High-Dose Therapy with Peripheral Blood Stem Cell Transplantation for Patients with Multiple Myeloma: Prognostic Impact of Chromosomal Aberrations and Correlation with the ISS-Score. Blood (ASH Annual Meeting Abstracts) 2008;112:2708.

53. Dimopoulos MA, Richardson P, Schlag R et al. Bortezomib-Melphalan-Prednisone (VMP) in Newly Diagnosed Multiple Myeloma Patients with Impaired Renal Function: Cohort Analysis of the Phase III VISTA Study. Blood (ASH Annual Meeting Abstracts) 2008;112:1727.

54. Bahlis NJ, Song KW, Fu T et al. The Impact of Cytogenetics on the Outcomes of Treatment with Lenalidomide Plus Dexamethasone in Relapsed or Refractory Multiple Myeloma. Blood (ASH Annual Meeting Abstracts) 2008;112:1731.

55. Carrasco DR, Tonon G, Huang Y et al. High-resolution genomic profiles define distinct clinico-pathogenetic subgroups of multiple myeloma patients. Cancer Cell. 2006;9:313-325.

56. Loiseau HA, Li C, Magrangeas F et al. In Myeloma, the Prognostic Impact of Hyperdiploidy Is Mainly Related to the Gain of Chromosome 5. Blood (ASH Annual Meeting Abstracts) 2008;112:632.

57. Shaughnessy JD, Jr., Zhan F, Burington BE et al. A validated gene expression model of high-risk multiple myeloma is defined by deregulated expression of genes mapping to chromosome 1. Blood. 2007;109:2276-2284.

58. Decaux O, Lode L, Magrangeas F et al. Prediction of survival in multiple myeloma based on gene expression profiles reveals cell cycle and chromosomal instability signatures in high-risk patients and hyperdiploid signatures in low-risk patients: a study of the Intergroupe Francophone du Myelome. J.Clin.Oncol. 2008;26:4798-4805.

59. Boccadoro M, Gavarotti P, Fossati G et al. Low plasma cell 3(H) thymidine incorporation in monoclonal gammopathy of undetermined significance (MGUS), smouldering myeloma and remission phase myeloma: a reliable indicator of patients not requiring therapy. Br.J Haematol. 1984;58:689-696.

60. Greipp PR, Katzmann JA, O'Fallon WM, Kyle RA. Value of beta 2-microglobulin level and plasma cell labeling indices as prognostic factors in patients with newly diagnosed myeloma. Blood. 1988;72:219-223.

61. San Miguel JF, Garcia-Sanz R, Gonzalez M et al. A new staging system for multiple myeloma based on the number of S-phase plasma cells. Blood. 1995;85:448-455.

62. Greipp PR, Lust JA, O'Fallon WM et al. Plasma cell labeling index and beta 2-microglobulin predict survival independent of thymidine kinase and C-reactive protein in multiple myeloma. Blood. 1993;81:3382-3387.

63. Gastinne T, Leleu X, Duhamel A et al. Plasma cell growth fraction using Ki-67 antigen expression identifies a subgroup of multiple myeloma patients displaying short survival within the ISS stage I. Eur.J.Haematol. 2007;79:297-304.

64. Zhan F, Huang Y, Colla S et al. The molecular classification of multiple myeloma. Blood. 2006;108:2020-2028.

65. Hose D, Reme T, Hielscher T et al. A Gene Expression Based Proliferation Index as Independent Prognostic Factor in Multiple Myeloma. Blood (ASH Annual Meeting Abstracts) 2008;112:1667.

66. Goldschmidt H, Sonneveld P, Cremer FW et al. Joint HOVON-50/GMMG-HD3 randomized trial on the effect of thalidomide as part of a high-dose therapy regimen and as maintenance treatment for newly diagnosed myeloma patients. Ann.Hematol. 2003;82:654-659.

67. Sonneveld P, van der Holt B, Schmidt-Wolf IGH et al. First Analysis of HOVON-65/GMMG-HD4 Randomized Phase III Trial Comparing Bortezomib, Adriamycine, Dexamethasone (PAD) Vs VAD as Induction Treatment Prior to High Dose Melphalan (HDM) in Patients with Newly Diagnosed Multiple Myeloma (MM). ASH Annual Meeting Abstracts 2008;112:653.

68. Harousseau JL, Attal M, Leleu X et al. Bortezomib plus dexamethasone as induction treatment prior to autologous stem cell transplantation in patients with newly diagnosed multiple myeloma: results of an IFM phase II study. Haematologica. 2006;91:1498-1505.

69. Barlogie B, Anaissie E, van RF et al. Incorporating bortezomib into upfront treatment for multiple myeloma: early results of total therapy 3. Br.J Haematol. 2007;138:176-185.

70. Rawstron AC, Orfao A, Beksac M et al. Report of the European Myeloma Network on multiparametric flow cytometry in multiple myeloma and related disorders. Haematologica. 2008;93:431-438.

71. Sarasquete ME, Garcia-Sanz R, Gonzalez D et al. Minimal residual disease monitoring in multiple myeloma: a comparison between allelic-specific oligonucleotide real-time quantitative polymerase chain reaction and flow cytometry. Haematologica. 2005;90:1365-1372.

72. Rawstron AC, Orfao A, Beksac M et al. Report of the European Myeloma Network on multiparametric flow cytometry in multiple myeloma and related disorders. Haematologica. 2008;93:431-438.

73. Sarasquete ME, Garcia-Sanz R, Gonzalez D et al. Minimal residual disease monitoring in multiple myeloma: a comparison between allelic-specific oligonucleotide real-time quantitative polymerase chain reaction and flow cytometry. Haematologica. 2005;90:1365-1372.

74. Bakkus MH, Bouko Y, Samson D et al. Post-transplantation tumour load in bone marrow, as assessed by quantitative ASO-PCR, is a prognostic parameter in multiple myeloma. Br.J Haematol. 2004;126:665-674.

75. Corradini P, Voena C, Tarella C et al. Molecular and clinical remissions in multiple myeloma: role of autologous and allogeneic transplantation of hematopoietic cells. J Clin.Oncol. 1999;17:208-215.

76. Cremer FW, Kiel K, Wallmeier M, Goldschmidt H, Moos M. A quantitative PCR assay for the detection of low amounts of malignant cells in multiple myeloma. Ann.Oncol 1997;8:633-636.

77. Lipinski E, Cremer FW, Ho AD, Goldschmidt H, Moos M. Molecular monitoring of the tumor load predicts progressive disease in patients with multiple myeloma after high-dose therapy with autologous peripheral blood stem cell transplantation. Bone Marrow Transplant. 2001;28:957-962.

78. Bradwell AR, Mead GP, Carr-Smith H. Serum free light chain analysis (plus Hevylite). Birmingham, UK: The Binding Site Ltd.; 2008.

79. Dispenzieri A, Kyle R, Merlini G et al. International Myeloma Working Group guidelines for serum-free light chain analysis in multiple myeloma and related disorders. Leukemia 2009;23:215-224.

80. Katzmann JA, Kyle RA, Benson J et al. Screening panels for detection of monoclonal gammopathies. Clin Chem. 2009;55:1517-1522.

81. Kuhnemund A, Liebisch P, Bauchmuller K et al. 'Light-chain escape-multiple myeloma'-an escape phenomenon from plateau phase: report of the largest patient series using LC-monitoring. J Cancer Res.Clin Oncol 2009;135:477-484.

82. Keren DF. Heavy/Light-Chain Analysis of Monoclonal Gammopathies. Clin Chem. 2009

83. Bradwell AR, Harding SJ, Fourrier NJ et al. Assessment of monoclonal gammopathies by nephelometric measurement of individual immunoglobulin kappa/lambda ratios. Clin.Chem. 2009;55:1646-1655.

84. Keren DF. Heavy/Light-Chain Analysis of Monoclonal Gammopathies. Clin Chem. 2009

85. Kompetenznetz Leukaemie. http://www.kompetenznetz-leukaemie.de/content/studieninfrastruktur/information/gcp/e20612/infoboxContent20614/GCP_Forbildung_Safety_Gkbuget_2009.pdf, Folie 23. 2009:

86. National Cancer Institute (NCI). http://ctep.cancer.gov/protocolDevelopment/electronic_applications/docs/resp_AE_rpt.ppt#298,10,Folie 10. 2009:

87. National Cancer Institute (NCI). https://webapps.ctep.nci.nih.gov/webobjs/ctc/webhelp/welcome_to_ctcae.htm - Reporting Adverse Events. 2009:

88. Personal Communication with G. Fingerle-Rowson, Ortho Biotech, May 2009. 2009. Ref Type: Personal Communication

89. Farrington CP, Manning G. Test statistics and sample size formulae for comparative binomial trials with null hypothesis of non-zero risk difference or non-unity relative risk. Stat.Med. 1990;9:1447-1454.

90. Barthel FM, Babiker A, Royston P, Parmar MK. Evaluation of sample size and power for multi-arm survival trials allowing for non-uniform accrual, non-proportional hazards, loss to follow-up and cross-over. Stat.Med. 2006;25:2521-2542.

91. Simon R. Optimal two-stage designs for phase II clinical trials. Control Clin.Trials 1989;10:1-10.

92. Newcombe RG. Interval estimation for the difference between independent proportions: comparison of eleven methods. Stat.Med. 1998;17:873-890.

93. Points to consider on switching between superiority and non-inferiority. Br.J.Clin.Pharmacol. 2001;52:223-228.

94. Marcus R, Peritz E, GABRIEL KR. On closed testing procedures with special reference to ordered analysis of variance. Biometrika 1976;63:655-660.

95. Blade J, Samson D, Reece D et al. Criteria for evaluating disease response and progression in patients with multiple myeloma treated by high-dose therapy and haemopoietic stem cell transplantation. Myeloma Subcommittee of the EBMT. European Group for Blood and Marrow Transplant. Br.J.Haematol. 1998;102:1115-1123.

96. Oken MM, Creech RH, Tormey DC et al. Toxicity and response criteria of the Eastern Cooperative Oncology Group. Am.J.Clin.Oncol. 1982;5:649-655.

97. The Criteria Committee of the New York Heart Association. *Nomenclature and Criteria for Diagnosis of Diseases of the Heart and Great Vessels*. 2009:

98. Moreau P, Pylypenko H, Grosicki S et al. Subcutaneous versus intravenous administration of bortezomib in patients with relapsed multiple myeloma: a randomised, phase 3, non-inferiority study. Lancet Oncol. 2011; 12: 431-40

99. Neben K, Lokhorst H, Jauch A, et al. Combining information regarding chromosomal aberrations t(4;14), del(17p13) and the copy number of 1q21 with the International Staging System classification allows stratification of myeloma patients undergoing autologous stem cell transplantation: results from the HOVON-65/GMMG HD4 trial. ASH Annual Meeting Abstracts 2011, Abstract No. 332 (oral presentation)

100 Attal M, Lauwers-Cances V, Marit G, et al. Lenalidomide Maintenance after Stem-Cell Transplantation for Multiple Myeloma. N Engl. J Med. 2012; 366: 1782-1791

101 Palumbo A, Hajek R, Delforge M, et al. Continuous Lenalidomide Treatment for Newly Diagnosed Multiple Myeloma. N Engl. J Med. 2012; 366: 1759-1769

102 McCarthy PL, Owzar K, Hofmeister CC, et al. Lenalidomide after stem-cell transplantation for multiple myeloma. N Engl. J Med. 2012; 366: 1770-1781
